# Supplementary material for: Nivolumab and ipilimumab in recurrent or refractory cancer of unknown primary: a phase II trial
Source: Nat Commun. 2023 Oct 24;14:6761. doi: 10.1038/s41467-023-42400-5 (PMC10598029; doi:10.1038/s41467-023-42400-5)
Supplement: Supplementary file 1 — Supplementary Information [file 41467_2023_42400_MOESM1_ESM.pdf]

Nivolumab and ipilimumab in recurrent or refractory cancer of unknown primary: a phase II trial

Maria Pouyiourou, Bianca N. Kraft, Timothy Wohlfromm, Michael Stahl, Boris Kubuschok, Harald Löffler, Ulrich T. Hacker, Gerdt Hübner, Lena Weiss, Michael Bitzer, Thomas Ernst, Philipp Schütt, Thomas Hielscher, Stefan Delorme, Martina Kirchner, Daniel Kazdal, Markus Ball, Klaus Kluck, Albrecht Stenzinger, Tilmann Bochtler & Alwin Krämer

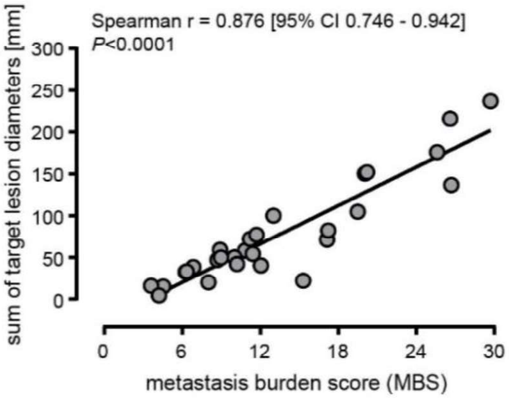

Supplementary Figure 1 | High correlation between matched sum of target lesion diameters and metastasis burden score (MBS). Scatter plot of baseline metastasis burden score, calculated as detailed in Supplementary Table 1, versus baseline sum of target lesion diameters according to RECIST v1.1 (*n*=29). Each dot represents a single patient. Correlation analyses were performed based on Spearman's correlation coefficient; a regression line was fitted. 95% CI, 95% confidence interval. Source data are provided as a Source Data file.

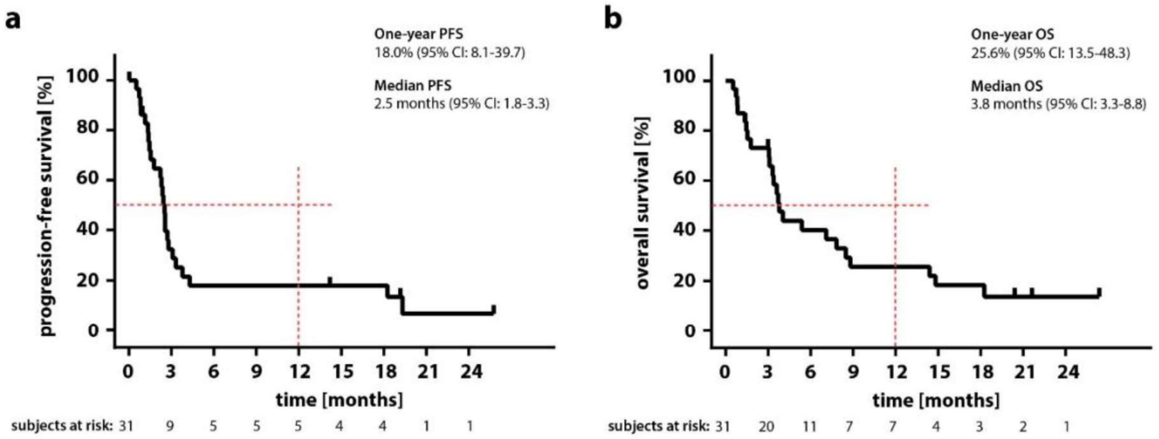

Supplementary Figure 2 | Progression-free survival (PFS) and overall survival (OS) for all patients of the CheCUP trial receiving combined nivolumab/ipilimumab. a Kaplan-Meier estimate of PFS for all patients (*n*=31). **b** Kaplan-Meier estimate of OS for all patients (*n*=31). Crosses denote censored observations, and for each time interval the number of patients at risk are indicated below the plots. The horizontal dashed lines mark the median values, the vertical dashed lines the one-year values. 95% CI, 95% confidence interval. Source data are provided as a Source Data file.

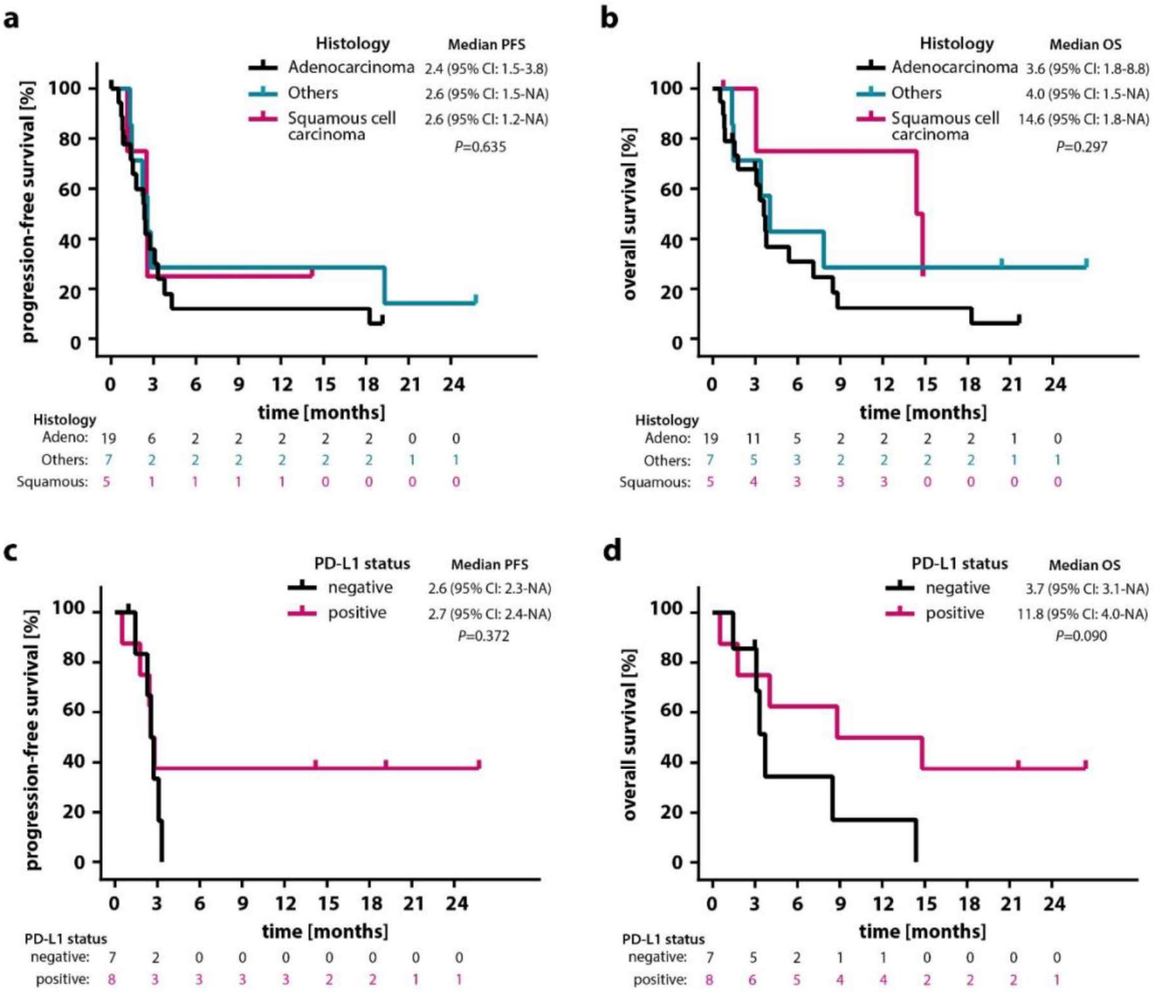

Supplementary Figure 3 | Patient outcome is neither affected by tumor histology nor by PD-L1 expression. a, b Kaplan-Meier estimates of **a** PFS and **b** OS, classified according to tumor histology: adenocarcinoma (*n*=19), squamous cell carcinoma (*n*=5) and others (*n*=7). Others included undifferentiated carcinoma and carcinoma with sarcomatoid differentiation. **c, d** Kaplan-Meier estimates of **c** PFS and **d** OS, classified according to PD-L1 expression status assessed per immunohistochemistry of baseline tumor biopsies: negative (*n*=7) and positive (*n*=8). Comparisons are made using a two-sided log-rank test, Cox proportional hazard regression modeling was used to calculate hazard ratio. 95% CI, 95% confidence interval; HR, hazard ratio. Source data are provided as a Source Data file.

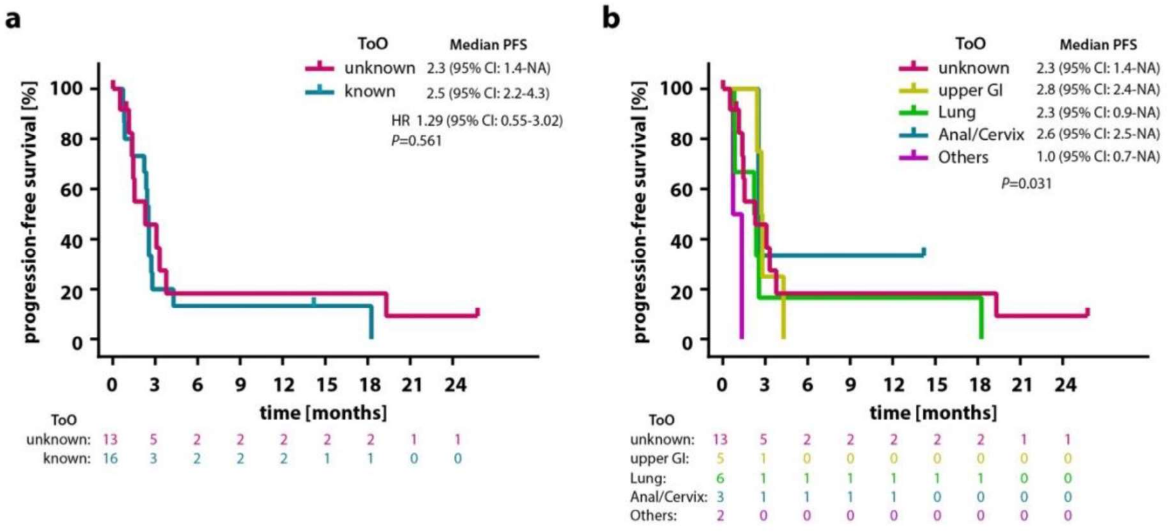

Supplementary Figure 4 | Patient outcome is not affected by putative CUP primary sites. Kaplan-Meier estimates of PFS, stratified **a** by clinical judgement into CUP cases with putative primary tumors being registered (known) versus cases in which the primary site was considered fully enigmatic (unknown) and **b** according to the putative primary sites identified: upper gastrointestinal (*n*=5, 16.1%), lung (*n*=6, 19.4%), anal/cervix (*n*=3, 9.7%), others (*n*=2, 6.4%), unknown (n=13, 41.9%). Comparisons are made using a two-sided log-rank test, Cox proportional hazard regression modeling was used to calculate hazard ratio. 95% CI, 95% confidence interval; HR, hazard ratio; ToO, tissue of origin; unknown, cancer of unknown primary; known, CUP cases in which a putative primary site was assigned by clinical judgement; GI, gastrointestinal. Source data are provided as a Source Data file.

a

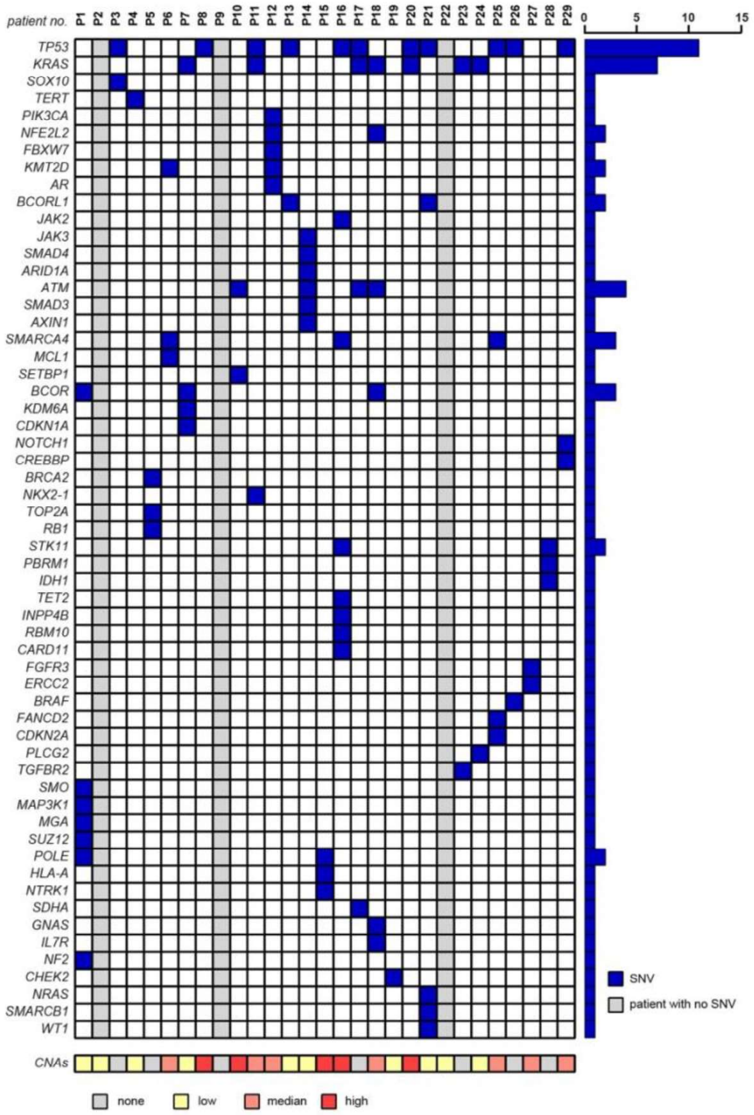
patient no.
P1 P2 P3 P4 P5 P6 P7 P8 P9 P10 P11 P12 P13 P14 P15 P16 P17 P18 P19 P20 P21 P22 P23 P24 P25 P26 P27 P28 P29
0 5 10 15
TP53
KRAS
SOX10
TERT
PIK3CA
NFE2L2
FBXW7
KMT2D
AR
BCORL1
JAK2
JAK3
SMAD4
ARID1A
ATM
SMAD3
AXIN1
SMARCA4
MCL1
SETBP1
BCOR
KDM6A
CDKN1A
NOTCH1
CREBBP
BRCA2
NKX2-1
TOP2A
RB1
STK11
PBRM1
IDH1
TET2
INPP4B
RBM10
CARD11
FGFR3
ERCC2
BRAF
FANCD2
CDKN2A
PLCG2
TGFBR2
SMO
MAP3K1
MGA
SUZ12
POLE
HLA-A
NTRK1
SDHA
GNAS
IL7R
NF2
CHEK2
NRAS
SMARCB1
WT1
SNV
patient with no SNV
CNAs
none
low
median
high

b

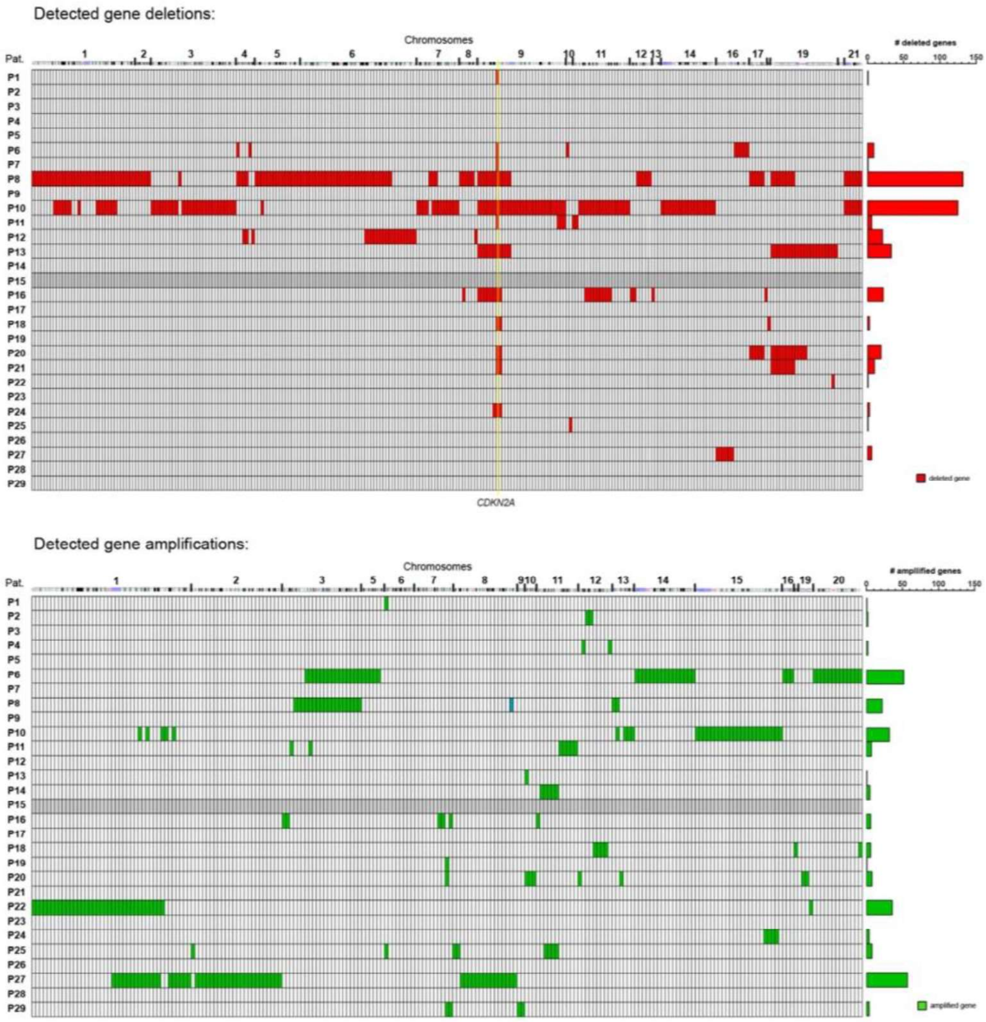

Supplementary Figure 5 | Baseline genomic landscape of the CheCUP cohort. a Oncoplot showing all potentially clinically relevant tumor gene SNVs/indels as assessed by comprehensive genomic profiling using the TSO500 panel in baseline tumor samples from the trial cohort (*n*=29). A column represents a patient; gray shaped columns indicate patients where no SNV/indels were detected. Numbers listed right represent the number of patients harboring an SNV/indel in the gene listed left. Bottom bars show categorization into groups with different frequency of detected CNAs (low: ≤3 CNAs, median: 3<CNAs<10, high: ≥10 CNAs). **b** Oncoplots showing a detailed listing of all detected gene deletions and amplifications on the genome as assessed by comprehensive genomic profiling with the TSO500 panel of baseline biopsy samples. A row represents a patient; gray shaped rows indicate patients where an unambiguous CNA profile statement was impossible. Numbers listed right represent the number of deleted or amplified genes in individual patients. Source data are provided in Supplementary Data 1.

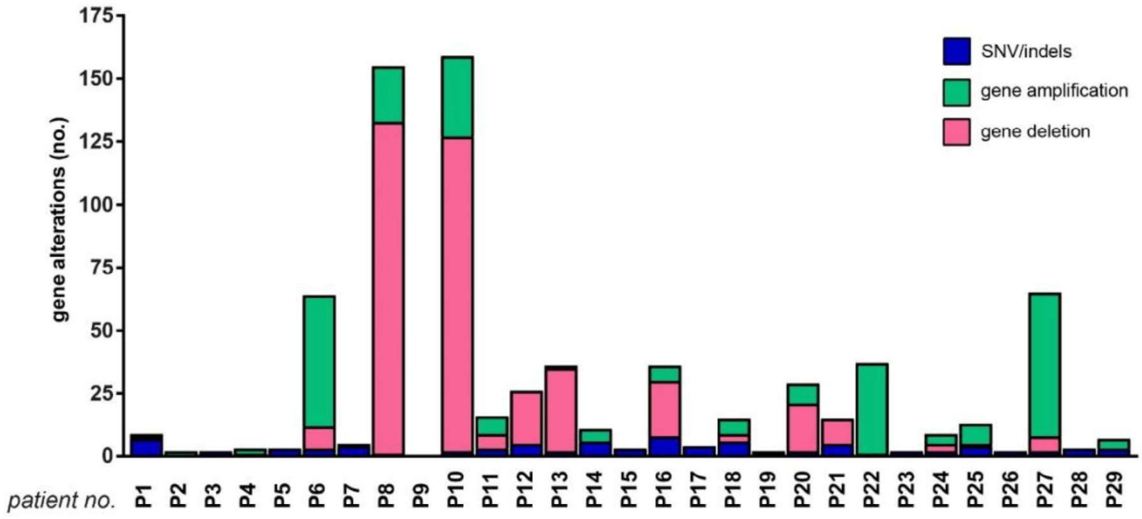

Supplementary Figure 6 | Summary of gene alterations detected in the CheCUP trial cohort (*n*=29). Individual patients harbored up to eight clinically relevant SNVs, 57 amplified and 132 deleted genes, or 159 molecular alterations in total. Source data are provided as a Source Data file.

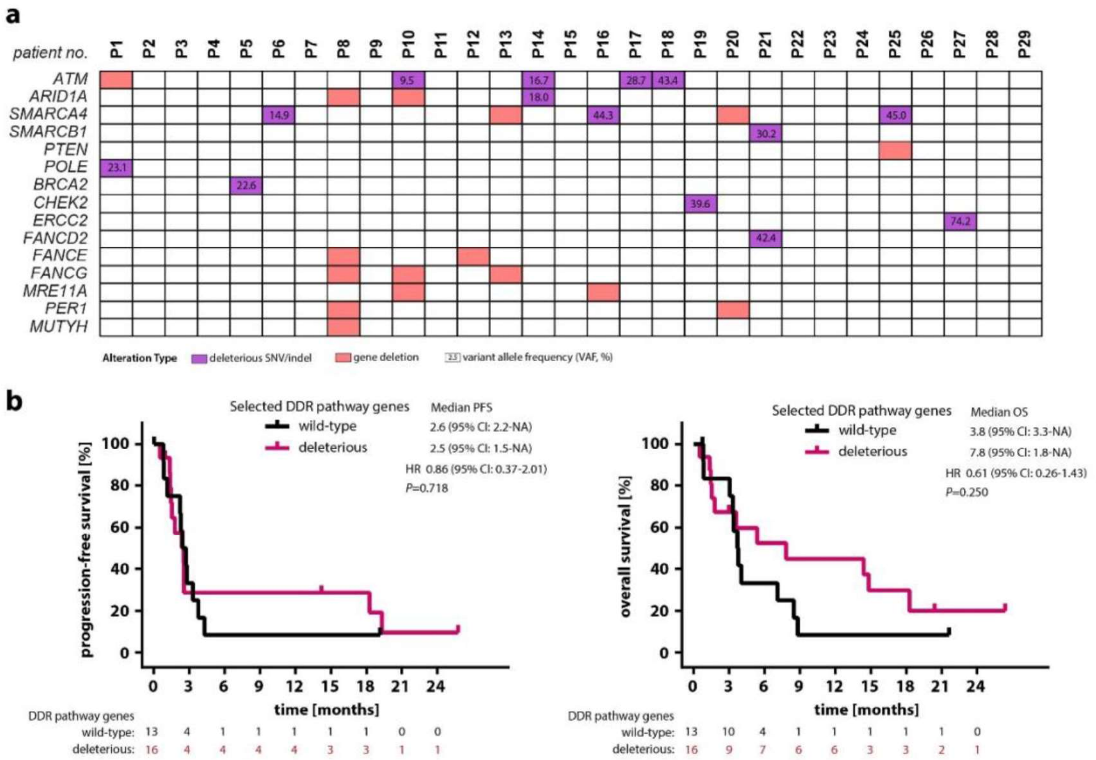

Supplementary Figure 7 | DNA damage repair (DDR) pathway gene alterations and patient outcome of combined nivolumab/ipilimumab treatment. a Oncoplot showing all detected potentially clinically relevant, functionally deleterious SNVs/indels in DDR pathway genes as well as whole DDR gene deletions detected in 3% or more patients as assessed by comprehensive genomic profiling with TSO500 panel of baseline CUP metastasis biopsy samples from the trial cohort (*n*=29). Numbers in the boxes indicate variant allele frequencies (VAF, %) of the detected SNVs/indels. Source data are provided in Supplementary Data 1. **b** Kaplan-Meier estimates of PFS and OS, stratified according to selected deleterious DDR pathway genes: deleterious (*n*=16) and wild-type (*n*=13). Comparisons are made using a two-sided log-rank test, Cox proportional hazard regression modeling was used to calculate hazard ratio. No adjustments for multiple comparisons were made. 95% CI, 95% confidence interval; HR, hazard ratio. Source data are provided as a Source Data file.

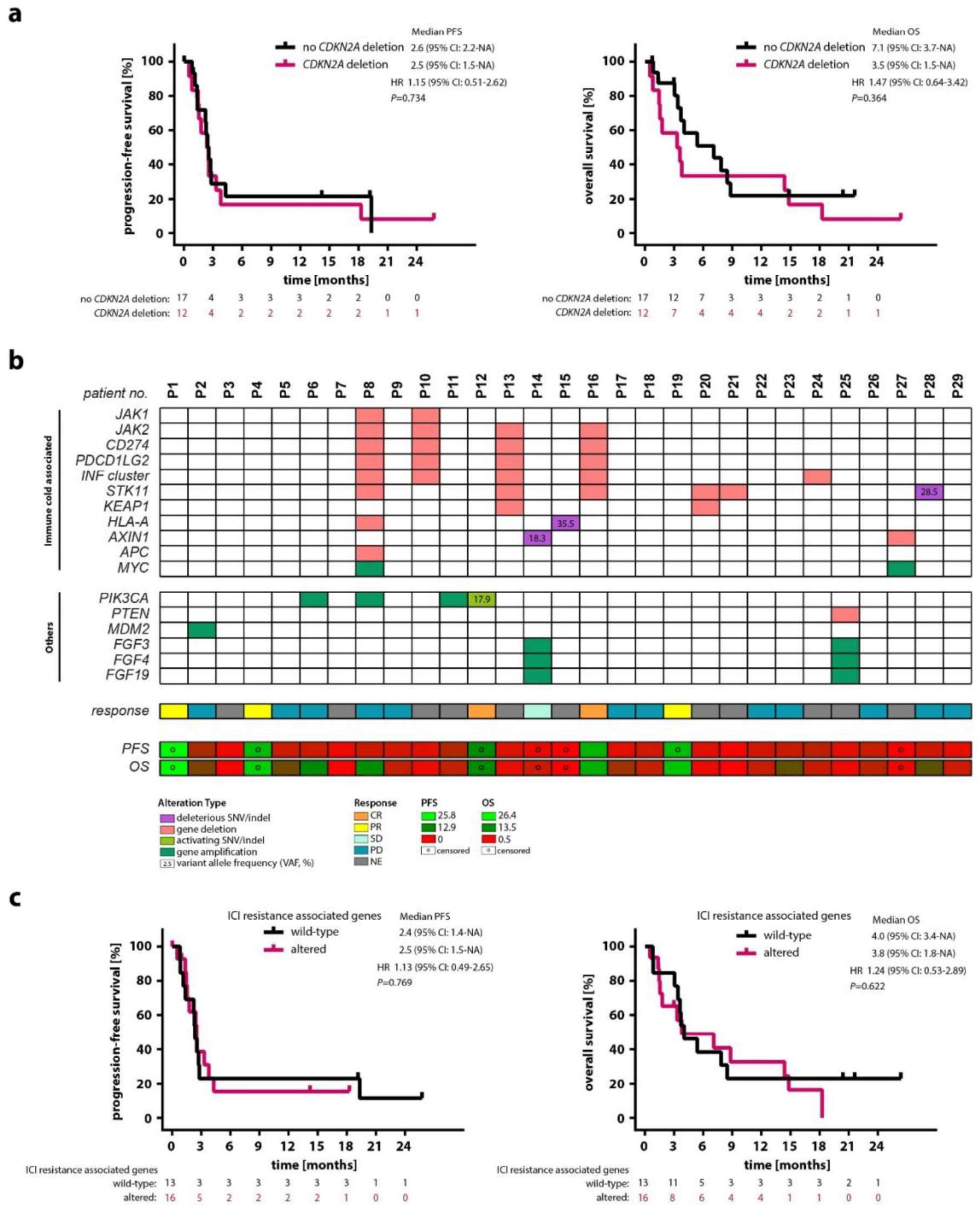
a
Median PFS
no CDKN2A deletion 2.6 (95% CI: 2.2-NA)
CDKN2A deletion 2.5 (95% CI: 1.5-NA)
HR 1.15 (95% CI: 0.51-2.62)
P=0.734
progression-free survival [%]
time [months]
no CDKN2A deletion: 17 4 3 3 3 2 2 0 0
CDKN2A deletion: 12 4 2 2 2 2 2 1 1
Median OS
no CDKN2A deletion 7.1 (95% CI: 3.7-NA)
CDKN2A deletion 3.5 (95% CI: 1.5-NA)
HR 1.47 (95% CI: 0.64-3.42)
P=0.364
overall survival [%]
time [months]
no CDKN2A deletion: 17 12 7 3 3 3 2 1 0
CDKN2A deletion: 12 7 4 4 4 2 2 1 1
b
patient no.
P1 P2 P3 P4 P5 P6 P7 P8 P9 P10 P11 P12 P13 P14 P15 P16 P17 P18 P19 P20 P21 P22 P23 P24 P25 P26 P27 P28 P29
Immune cold associated
JAK1
JAK2
CD274
PDCD1LG2
INF cluster
STK11
KEAP1
HLA-A
AXIN1
APC
MYC
28.5
35.5
18.3
Others
PIK3CA
PTEN
MDM2
FGF3
FGF4
FGF19
17.9
response
PFS
OS
Alteration Type
deleterious SNV/indel
gene deletion
activating SNV/indel
gene amplification
2.5 variant allele frequency (VAF, %)
Response
CR
PR
SD
PD
NE
PFS
25.8
12.9
0
censored
OS
26.4
13.5
0.5
censored
c
ICI resistance associated genes
wild-type
altered
Median PFS
2.4 (95% CI: 1.4-NA)
2.5 (95% CI: 1.5-NA)
HR 1.13 (95% CI: 0.49-2.65)
P=0.769
progression-free survival [%]
time [months]
ICI resistance associated genes
wild-type: 13 3 3 3 3 3 3 1 1
altered: 16 5 2 2 2 2 1 0 0
Median OS
4.0 (95% CI: 3.4-NA)
3.8 (95% CI: 1.8-NA)
HR 1.24 (95% CI: 0.53-2.89)
P=0.622
overall survival [%]
time [months]
wild-type: 13 11 5 3 3 3 3 2 1
altered: 16 8 6 4 4 1 1 0 0

Supplementary Figure 8 | Neither *CDKN2A* deletion nor altered ICI resistance associated genes are associated with patient outcome with combined nivolumab/ipilimumab treatment. **a** Kaplan-Meier estimates of PFS and OS, stratified according to *CDKN2A* alterations: deletion (*n*=17) versus wild-type (*n*=12). **b** Oncoplot showing potentially clinically relevant tumor gene alterations (SNVs/indels, gene deletions and amplifications) in ICI resistance associated genes of 3% or more patients as assessed by comprehensive genomic profiling of baseline CUP metastasis biopsy samples from trial cohort (*n*=29). Most selected gene alterations were associated with immune cold tumors. A column represents a patient. Bottom bars show radiological response assessment as well as heatmaps of PFS and OS (both in months, censored patients marked with dots). **c** Kaplan-Meier estimates of PFS and OS, stratified according to altered ICI resistance associated genes: altered (*n*=16) versus wild-type (*n*=13). In (**a, c**), comparisons are made using a two-sided log-rank test, Cox proportional hazard regression modeling was used to calculate hazard ratio. 95% CI, 95% confidence interval; HR, hazard ratio; CR, complete response; PR, partial response; SD, stable disease; PD, progressive disease; NE, not evaluable. Source data are provided in Supplementary Data 1 and in the Source Data file.

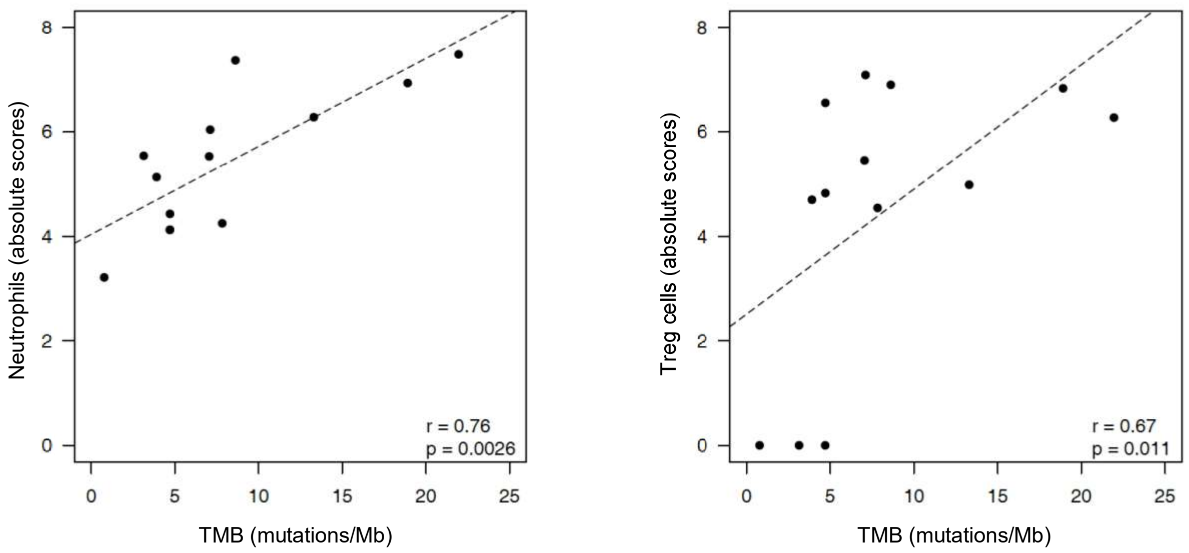

Supplementary Figure 9 | Tumor mutational burden (TMB) correlates with neutrophil and Treg cell infiltration of the CUP tumor microenvironment. Scatter plots of TMB versus the abundance of neutrophils or regulatory T (Treg) cells, as estimated from gene expression profiles using a 770-gene panel (NanoString nCounter technology). Each dot represents a single patient (*n*=13). Correlation analyses were performed based on Spearman's correlation coefficients. Source data are provided in Supplementary Data 4.

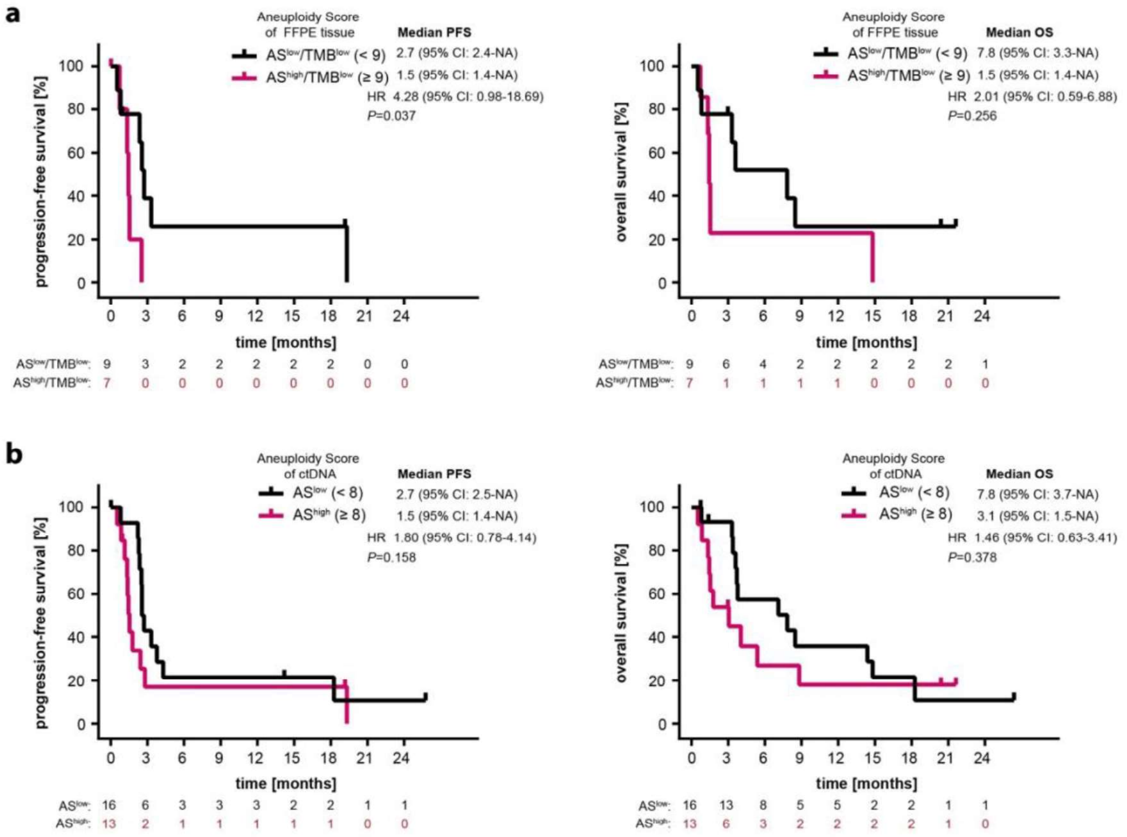

Supplementary Figure 10 | Patient outcome correlates with aneuploidy levels of tumor tissue. Kaplan-Meier estimates of PFS and OS, stratified into patients with low versus high aneuploidy scores, as determined in **a** FFPE tumor tissue of the TMB^{low} CheCUP population (n=16) and **b** ctDNA at baseline before start of study treatment of CheCUP patients irrespective of their TMB level (n=29). Comparisons are made using a two-sided log-rank test. Cox proportional hazard regression modeling was used to calculate hazard ratio. 95% CI, 95% confidence interval; HR, hazard ratio; CR, complete response; PR, partial response; SD, stable disease; PD, progressive disease; FFPE, formalin-fixed paraffin-embedded; AS, aneuploidy score; TMB, tumor mutational burden; PFS, progression-free survival; OS, overall survival; ctDNA, circulating tumor DNA. Source data are provided as a Source Data file.

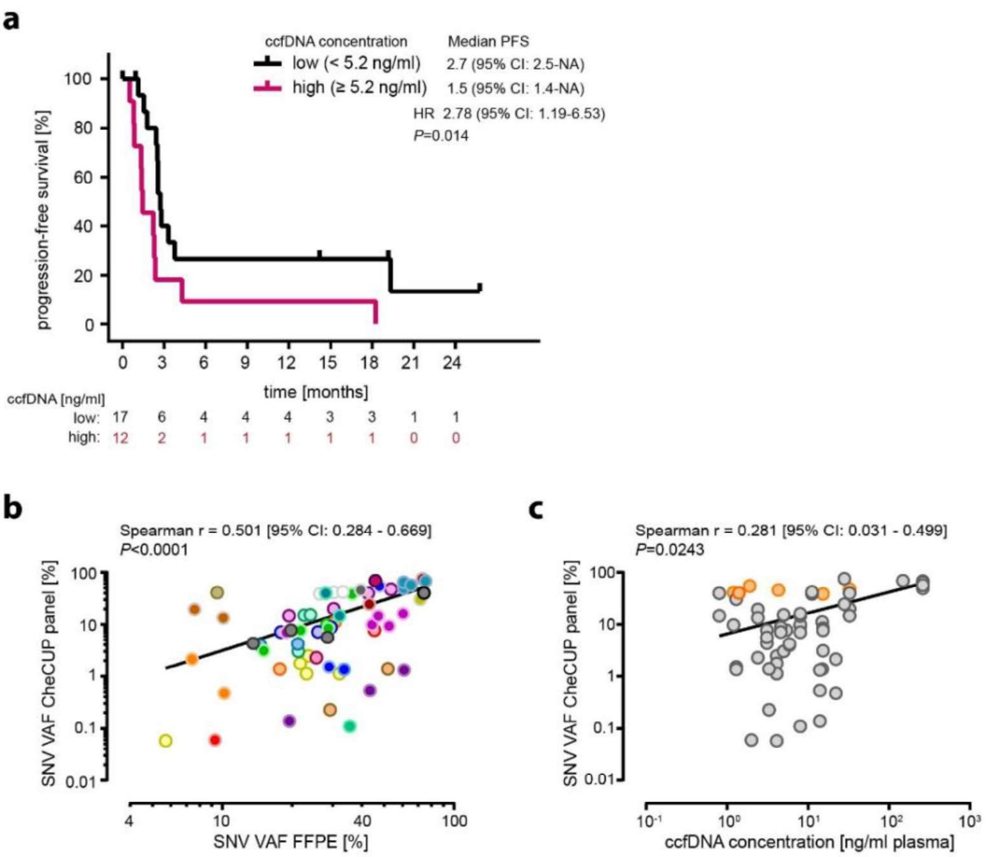

Supplementary Figure 11 | ctDNA analyses by targeted NGS with the customized CheCUP panel. a Kaplan-Meier estimate of PFS, stratified according to high (*n*=12) or low (*n*=17) ccfDNA based on a ccfDNA concentration cut-off of 5.2 ng/ml plasma. Comparisons are made using a two-sided log-rank test, Cox proportional hazard regression modeling was used to calculate hazard ratio. No adjustments for multiple comparisons were made. 95% CI, 95% confidence interval; HR, hazard ratio. **b** Scatter plot of SNV/indel VAFs, detected by targeted NGS of patient-specific hotspot mutations with the CheCUP panel, versus matched SNV/indel VAFs, detected by comprehensive genomic profiling with the TSO500 panel, in plasma and FFPE tissue samples of CUP patients (*n*=64), respectively. Each dot represents a single SNV/indel; SNV/indels from the same CUP patient are indicated by the same color. **c** Scatter plot of baseline ccfDNA concentration versus baseline ctDNA SNV VAFs. Each dot represents a single SNV/indel (*n*=64); orange dots indicate heterozygous germline mutations confirmed by whole exome sequencing of healthy peripheral blood mononuclear cells from same CUP patients. In (**b, c**), correlation analyses were performed based on Spearman's correlation coefficient; a regression line was fitted. Of note, the log-log axes are for display purpose only. Source data are provided as a Source Data file.

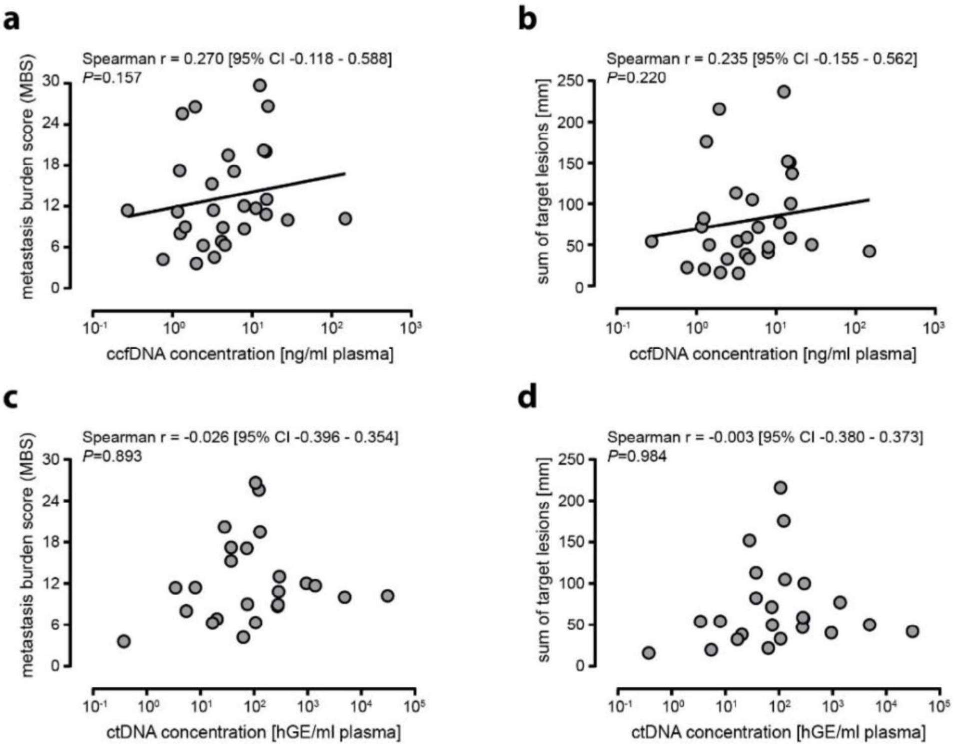

Supplementary Figure 12 | Baseline ccfDNA and ctDNA levels do not correlate with either sum of target lesion diameters or the metastasis burden score. a, b Scatter plots of baseline ccfDNA concentration versus **a** metastasis burden score (MBS), calculated as detailed in Supplementary Table S1, or **b** sum of target lesion diameters according to RECIST v1.1. **c, d** Scatter plots of baseline ctDNA concentration versus **c** metastasis burden score (MBS) or **d** sum of target lesion diameters according to RECIST v1.1. In (**a-d**), each dot represents a single patient (*n*=29). Correlation analyses were performed based on Spearman's correlation coefficient; a regression line was fitted. Of note, the x-axes are in log-scale for display purpose only. 95% CI, 95% confidence interval. Source data are provided as a Source Data file.

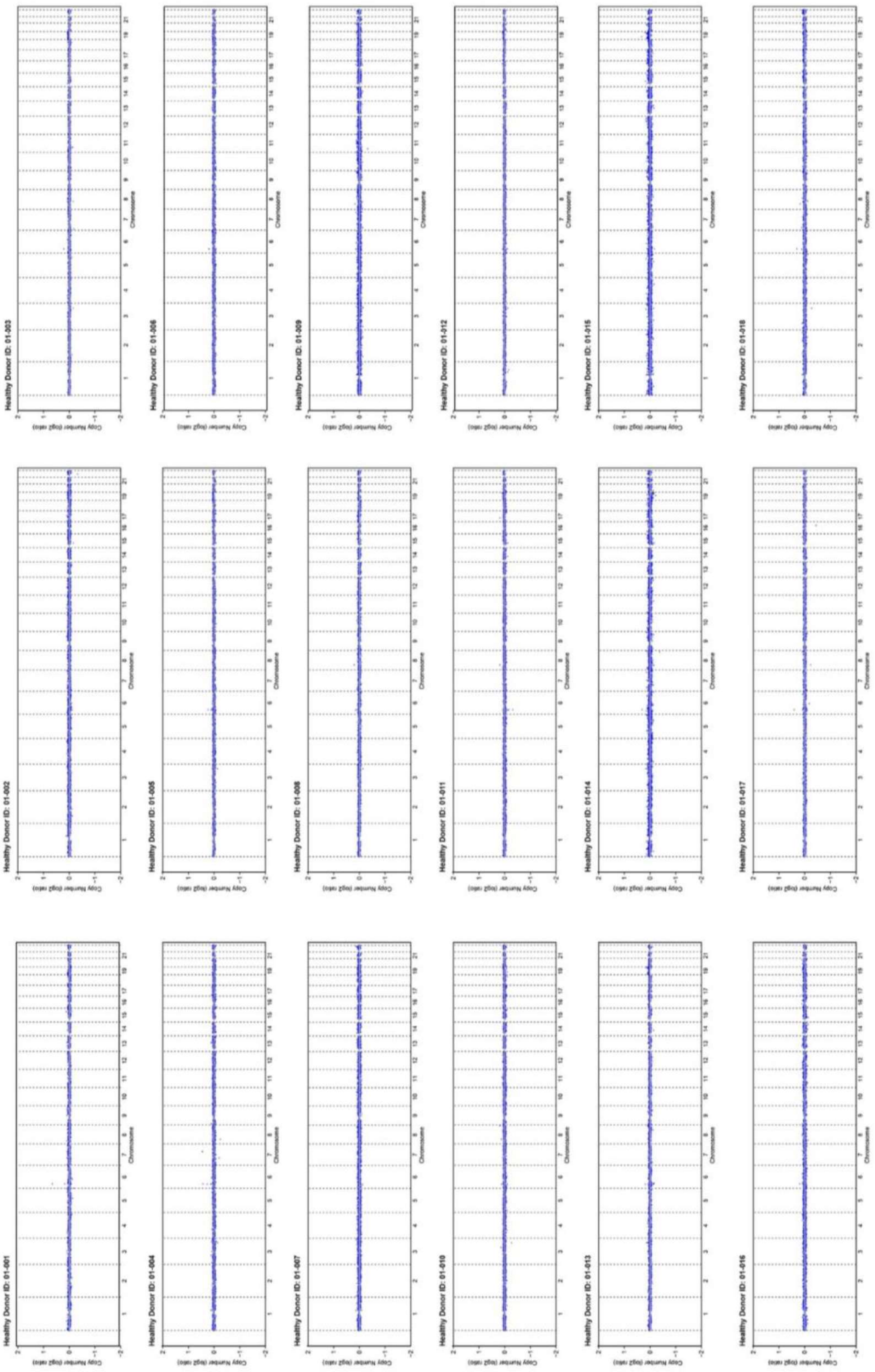

Supplementary Figure 13 | Genome-wide CNA profiles inferred from sWGS of plasma ccfDNA from 19 healthy individuals intended as reference panel for CNA analysis of ccfDNA from CUP patients.

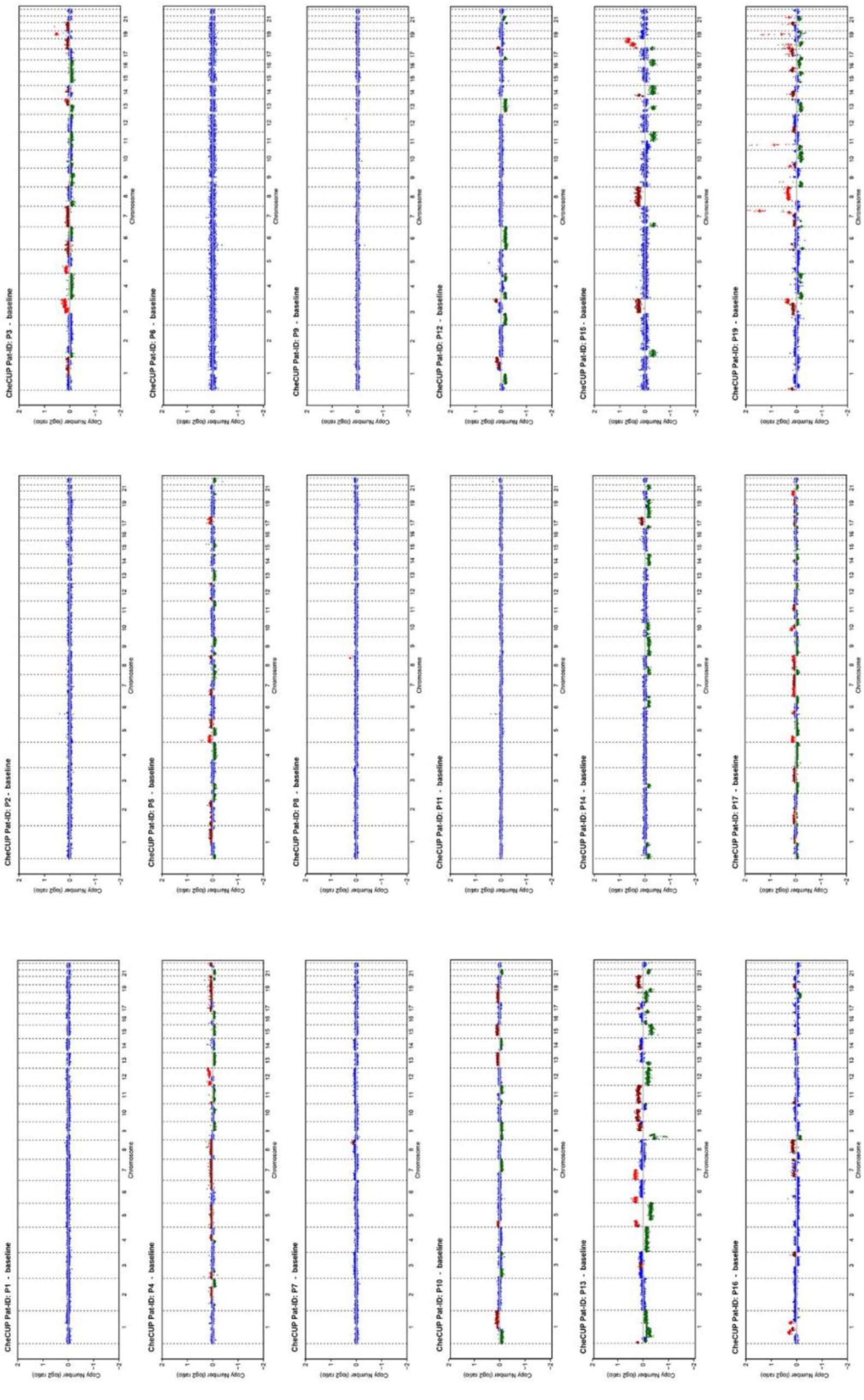

Supplementary Figure 14 | Representative examples of genome-wide CNA profiles inferred from sWGS of baseline plasma ccfDNA from CUP patients. TFx estimates >4% could be identified. Chromosome regions in shades of red indicate CNA gains, regions in green CNA losses.

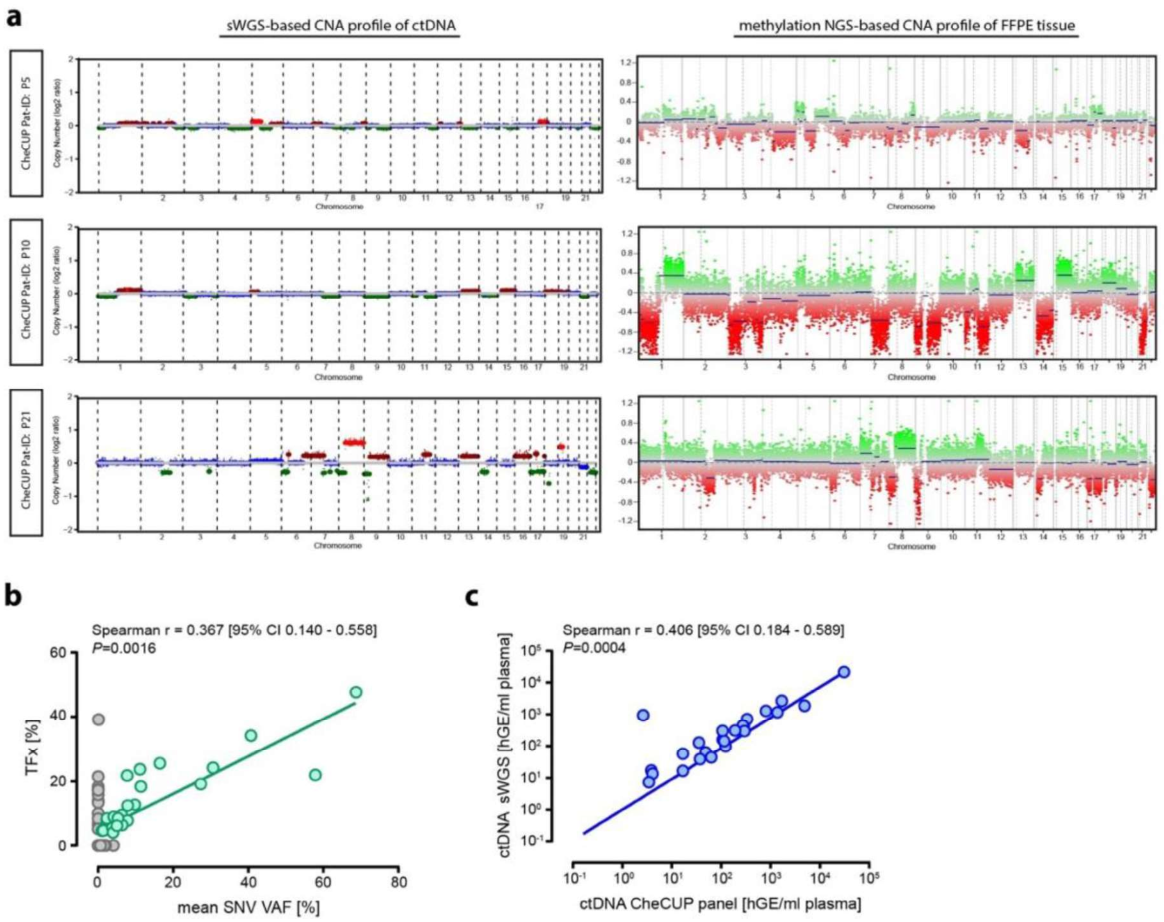

Supplementary Figure 15 | Combined targeted NGS of patient-specific hotspot mutations and sWGS-based CNA profiling strategy to detect ctDNA levels in patients with unfavorable CUP. a Three representative examples of CUP patients demonstrating that sWGS-based CNA analysis of ctDNA (*left panels*, chromosome regions in shades of red indicate CNA gains, chromosome regions in green indicate CNA losses) yields in results comparable to CNA assessment by methylation profiling of matched FFPE tumor biopsy samples (*right panels*, copy number gains in green, copy number losses in red). **b** Correlation between TFx estimates and mean SNV VAFs detected in the same plasma samples (*n*=71) by sWGS-based CNA profiling and targeted NGS of patient-specific hotspot mutations, respectively. The diagram shows the raw values of the individual sequencing approaches: each dot represents a plasma sample. Gray dots highlight samples in which either no TFx values or no SNVs/indels were detectable. Of note, even with relatively high TFx values no SNV/indels could be detected in some samples by targeted NGS. **c** Correlation between ctDNA contents in haploid genome equivalents (hGE)/ml plasma, as calculated from sWGS-based TFx estimates and mean ctDNA VAFs detected by targeted NGS. Of note, log-log axes are for display purpose only. In contrast to **b**, only samples (*n*=29) in which the ctDNA content could be determined by both sequencing strategies are shown. In **b, c** correlation analyses were performed based on Spearman's correlation coefficient; a regression line was fitted. Source data are provided as a Source Data file.

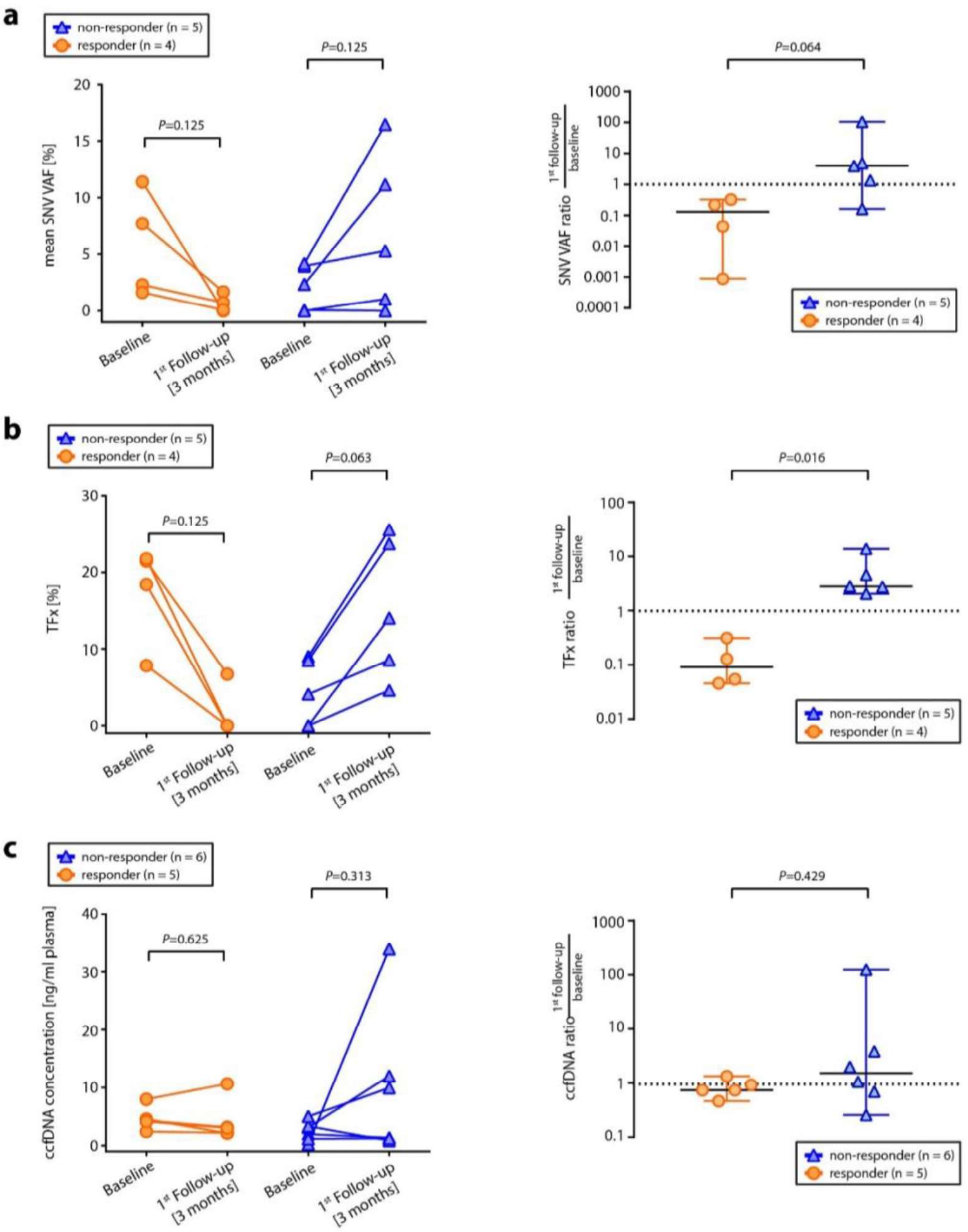
a
non-responder (n = 5)
responder (n = 4)
P=0.125
P=0.125
mean SNV VAF [%]
Baseline
1st Follow-up [3 months]
Baseline
1st Follow-up [3 months]
P=0.064
SNV VAF ratio 1st follow-up / baseline
b
non-responder (n = 5)
responder (n = 4)
P=0.063
P=0.125
TFx [%]
P=0.016
TFx ratio 1st follow-up / baseline
c
non-responder (n = 6)
responder (n = 5)
P=0.313
P=0.625
ccfDNA concentration [ng/ml plasma]
P=0.429
ccfDNA ratio 1st follow-up / baseline

Supplementary Figure 16 | ctDNA and ccfDNA levels in patients responding versus non-responding to immune checkpoint inhibitor treatment. Changes in **a** mean ctDNA SNV VAFs, **b** TFx estimates and **c** ccfDNA levels in paired baseline and first follow-up plasma samples after three months of ICI treatment of radiologically responding (orange) and non-responding (blue) patients (left panels). Each dot/triangle represents a single CUP patient. Right panels depict comparisons between the molecular responses of radiologically responding (orange) and non-responding patients (blue), calculated as the ratio of first follow-up to baseline sample (the y-axis is in log-scale for display purpose only). Each dot/triangle represents the molecular response of a single patient, bold horizontal bars indicate the median values +/- 95% confidence interval; a dashed horizontal line indicates the cut-off predicting response and outcome. For both targeted NGS and sWGS sequencing, no ctDNA could be detected in one responder and non-responder, respectively. Comparisons between baseline and first follow-up samples of the same patient were made using either a two-tailed Wilcoxon matched-pairs signed rank test or a two-tailed Mann-Whitney U test. Source data are provided as a Source Data file.

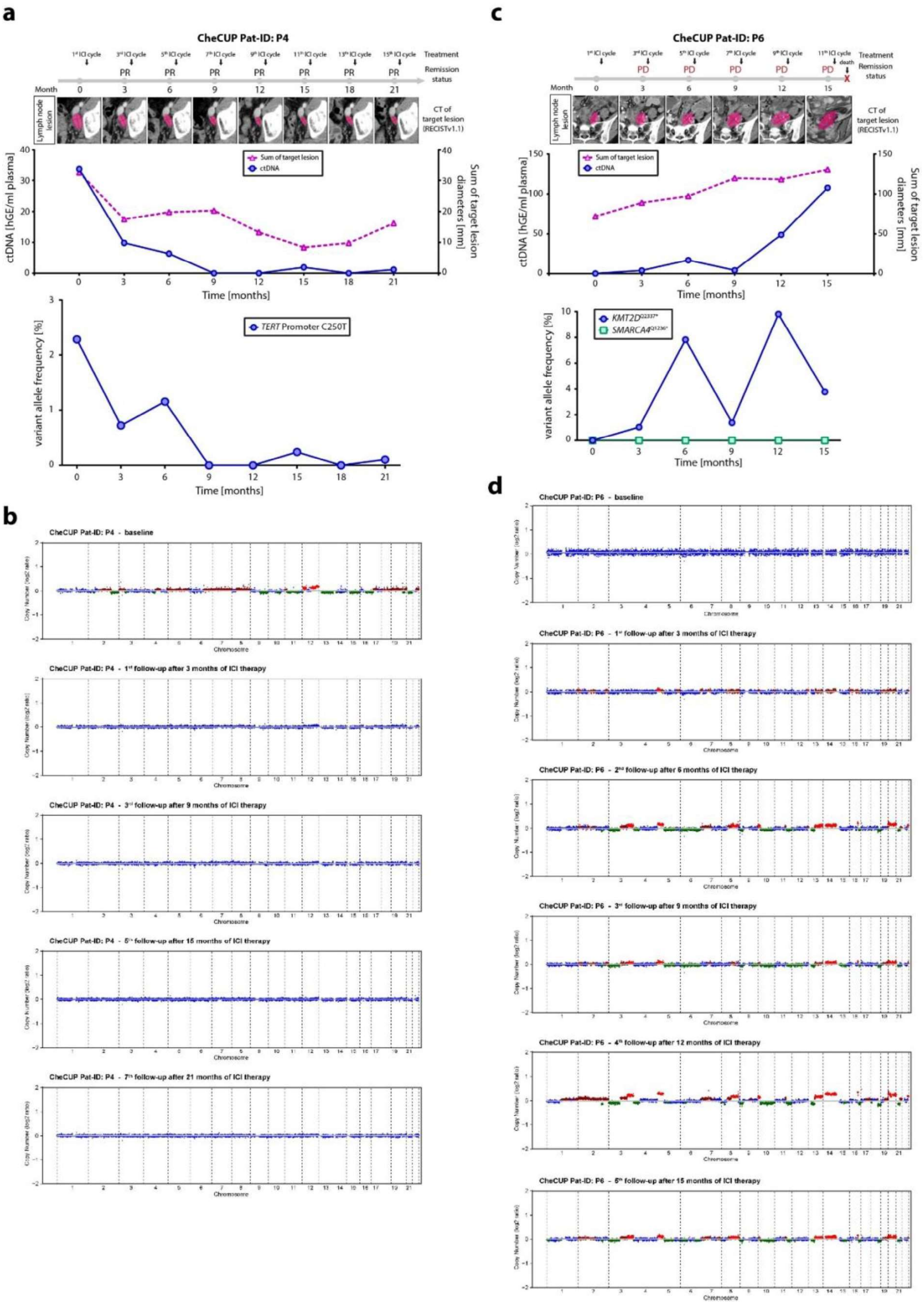
a
CheCUP Pat-ID: P4
Treatment
Remission status
Month
CT of target lesion (RECISTv1.1)
Lymph node lesion
Sum of target lesion
ctDNA
ctDNA [hGE/ml plasma]
Sum of target lesion diameters [mm]
Time [months]
TERT Promoter C250T
variant allele frequency [%]
b
CheCUP Pat-ID: P4 - baseline
CheCUP Pat-ID: P4 - 1st follow-up after 3 months of ICI therapy
CheCUP Pat-ID: P4 - 3rd follow-up after 9 months of ICI therapy
CheCUP Pat-ID: P4 - 5th follow-up after 15 months of ICI therapy
CheCUP Pat-ID: P4 - 7th follow-up after 21 months of ICI therapy
c
CheCUP Pat-ID: P6
death
KMT2D Q2337*
SMARCA4 Q1236*
d
CheCUP Pat-ID: P6 - baseline
CheCUP Pat-ID: P6 - 1st follow-up after 3 months of ICI therapy
CheCUP Pat-ID: P6 - 2nd follow-up after 6 months of ICI therapy
CheCUP Pat-ID: P6 - 3rd follow-up after 9 months of ICI therapy
CheCUP Pat-ID: P6 - 4th follow-up after 12 months of ICI therapy
CheCUP Pat-ID: P6 - 5th follow-up after 15 months of ICI therapy
Chromosome

Supplementary Figure 17 | Serial tumor-specific CNA profiling by sWGS parallels the dynamics of somatic tumor SNVs detected by targeted NGS. a, b Monitoring of tumor burden and ctDNA changes in a case (CheCUP patient P4) responding to combined nivolumab/ipilimumab therapy. **a** *Upper graph:* Comparison of ctDNA level in serial collected plasma samples with the measured sum of target lesion diameters by RECIST v1.1. On-treatment ctDNA analyses by targeted NGS were consistent with the radiological response assessment, showing complete molecular response after nine months of ICI treatment. hGE, haploid genome equivalent. Source data are provided as a Source Data file. *Lower graph*: Dynamic tracking of the *TERT* promotor C250T SNV VAF. **b** Dependent on the detection limit of TFx>4%, genome-wide CNA profiles inferred from sWGS showed complete molecular response already after three months of ICI treatment. Chromosome regions in shades of red indicate CNA gains, regions in green CNA losses. **c, d** Monitoring of tumor burden and ctDNA changes in a case (CheCUP patient P6) not responding to combined nivolumab/ipilimumab therapy. **c** *Upper graph:* Comparison of ctDNA level in serial collected plasma samples with the measured sum of target lesion diameters by RECIST v1.1. On-treatment ctDNA analyses by targeted NGS were consistent with the radiological response assessment: ctDNA undetectable at baseline dramatically increased at disease progression and again before patient's death. hGE, haploid genome equivalent. Source data are provided as a Source Data file. *Lower graph*: Detection of a subclonal *KMT2D*-p.Q2337* treatment-resistant mutation by dynamic tracking of VAF from single somatic tumor mutations. **d** Genome-wide CNA profiles inferred from sWGS were consistent with the dynamics of somatic tumor SNVs detection by targeted NGS. Chromosome regions in shades of red indicate CNA gains, regions in green CNA losses.

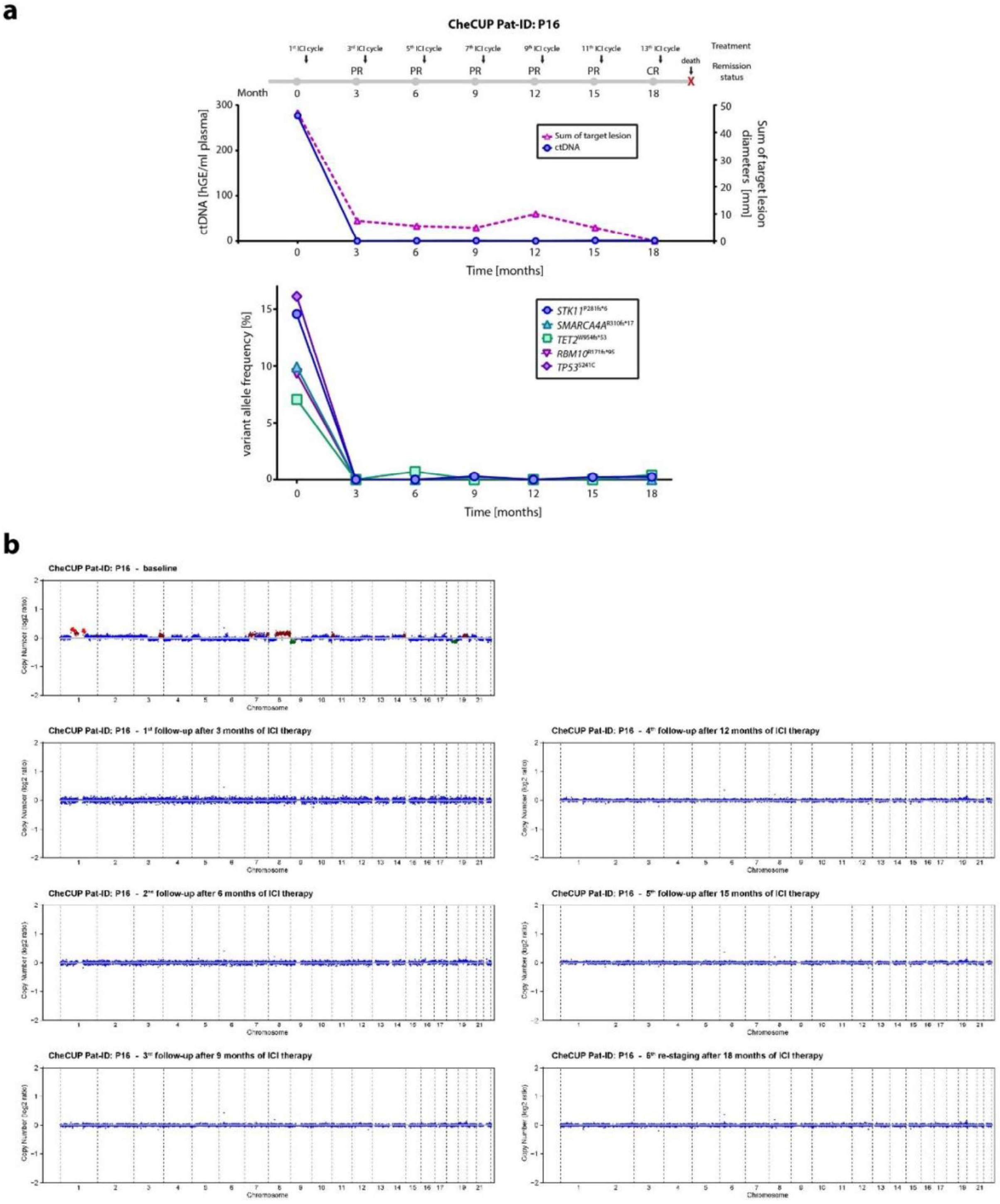
a
CheCUP Pat-ID: P16
Treatment
Remission status
death
PR
CR
Month
Sum of target lesion
ctDNA
ctDNA [hGE/ml plasma]
Sum of target lesion diameters [mm]
Time [months]
STK11 P281fs*6
SMARCA4A R3106fs*17
TET2 W954fs*53
RBM10 R171fs*95
TP53 S241C
variant allele frequency [%]
b
CheCUP Pat-ID: P16 - baseline
CheCUP Pat-ID: P16 - 1st follow-up after 3 months of ICI therapy
CheCUP Pat-ID: P16 - 4th follow-up after 12 months of ICI therapy
CheCUP Pat-ID: P16 - 2nd follow-up after 6 months of ICI therapy
CheCUP Pat-ID: P16 - 5th follow-up after 15 months of ICI therapy
CheCUP Pat-ID: P16 - 3rd follow-up after 9 months of ICI therapy
CheCUP Pat-ID: P16 - 6th re-staging after 18 months of ICI therapy
Copy Number (log2 ratio)
Chromosome

Supplementary Figure 18 | Both targeted NGS of patient-specific hotspot mutations and tumor-specific CNA profiling by sWGS predicts treatment response several months earlier than radiological response assessment. a, b Representative case (CheCUP patient P16) exemplifying the utility of ctDNA analyses in monitoring ICI response in parallel to radiological assessment. **a** *Upper graph:* Comparison of ctDNA level in serial collected plasma samples with the measured sum of target lesion diameters by RECIST v1.1. On-treatment ctDNA analyses by targeted NGS predicted complete response to combined nivolumab/ipilimumab already after three months of ICI treatment. hGE, haploid genome equivalent. *Lower graph*: Dynamic tracking of VAF from single somatic tumor mutations in ctDNA. Source data are provided as a Source Data file. **b** Genome-wide CNA profiles inferred from sWGS were consistent with the dynamics of somatic tumor SNVs detection by targeted NGS. Chromosome regions in shades of red indicate CNA gains, regions in green CNA losses.

Supplementary Table 1 | Metastasis burden score (MBS). The metastasis burden was calculated as the sum of organ involvement points added to the sum of diameters of all target lesions in cm according to RECIST v1.1. Low disease burden 0-9; intermediate disease burden 10-13; high disease burden >13.

Organ	Manifestation	Score points
Liver	1 lesion	1
	2-3 lesions	2
	>3 lesions	3
Lung	1 lesion	1
	2-3 lesions	2
	>3 lesions	3
Bones	1 lesion	1
	2-3 lesions	2
	>3 lesions	3
Soft tissue	1 lesion	1
	2-3 lesions	2
	>3 lesions	3
Lymph nodes	1 lymph node	1
	1 lymph node region	2
	At least 2 lymph node regions	3
Peritoneum / Pleura	Effusion compatible but without proof of carcinomatosis	1
	Carcinomatosis of either peritoneum or pleura	2
	Carcinomatosis of both peritoneum and pleura	3
Additional metastases in further organs	1 metastasis in 1 organ	1
	2 separate metastases in 1 organ or 2 separate organs with 1 metastasis in each organ	2
	At least 2 additional organs with multiple metastases in at least 1 of these organs	3

Supplementary Table 2 | PD-L1 status. PD-L1 expression status was evaluated either as part of the initial diagnostic workup or within the CheCUP trial, whenever FFPE tissue was available. Source data are provided as a Source Data file.

	**Total**	**TMB^{high}**	**TMB^{low}**
CPS			
< 1	6	1	5
≥ 1	2	2	0
≥ 10	4	1	3
≥ 50	2	0	2
TPS			
< 1	8	2	6
≥ 1	3	2	1
≥ 10	2	0	2
≥ 50	1	0	1
IC-Score			
<1	4	0	4
1-2	5	3	2
≥ 3	2	0	2

CPS, combined positive score; TPS, tumor proportion score; IC, immune cell

Supplementary Table 3 | Treatment response according to disease histology. Response assessment was performed by the trial radiologists according to RECIST v1.1 every second treatment cycle. Source data are provided as a Source Data file.

	**Total**	**Adeno-carcinoma**	**Squamous cell carcinoma**	**Poorly differentiated carcinoma**	**Carcinoma with sarcomatoid differentiation**
**Patients (n)**	31	20	5	4	2
**Overall response n (%)**	5 (16.1)	2 (10)	1 (20)	1 (25)	1 (50)
**Best overall response**					
Complete response	2 (6.5)	1 (5)	1 (20)	0	0
Partial response	3 (9.7)	1 (5)	0	1 (25)	1 (50)
Stable disease	1 (3.2)	1 (5)	0	0	0
Progressive disease	12 (38.7)	7 (35)	3 (60)	2 (50)	0
Early study discontinuation	13 (41.9)	10 (50)	1 (20)	1 (25)	1 (50)

Supplementary Table 4 | Progression-free and overall survival depending on the levels of 14 immune cell populations in tumor tissue from 13 patients as determined by targeted mRNA expression profiling using the NanoString nCounter gene expression platform in pretreatment tumor samples. Absolute scores for cell types above the median were classified as high and those below the median as low. Median overall and progression-free survival is calculated using the Kaplan-Meier method. Comparisons are made using a two-sided log-rank test, Cox proportional hazard regression modeling was used to calculate hazard ratios. Source data are provided as a Source Data file.

Cell type	Overall survival					Progression-free survival				
	Median OS (months)		Log-rank *P*	HR	95% CI	Median PFS (months)		Log-rank *P*	HR	95% CI
	Low	High				Low	High			
B cells	3.6	8.5	0.27	0.46	0.11-1.91	2	2.7	0.18	0.32	0.06-1.66
$CD45^+$ cells	1.5	14	0.17	0.37	0.08-1.63	1.5	2.6	0.25	0.45	0.12-1.85
$CD8^+$ T cells	5.7	18	0.54	0.64	0.15-2.69	2.5	0.82	0.7	0.73	0.17-3.04
Cytotoxic cells	1.5	14	0.17	0.37	0.08-1.63	1.5	2.6	0.25	0.48	0.12-1.85
Dendritic cells	1.5	8.5	0.63	0.72	0.19-2.77	1.5	2.6	0.61	0.73	0.19-2.80
Exhausted $CD8^+$ cells	3.6	7.8	0.54	1.51	0.40-5.74	2.4	1.7	0.3	2.2	0.55-8.88
Macrophages	3.6	4.3	0.66	1.35	0.35-5.14	2.4	1.7	0.64	1.42	0.38-5.39
Mast cells	1.5	8.5	0.16	0.35	0.07-1.63	1.5	2.6	0.14	0.35	0.08-1.45
Neutrophils	1.5	14	0.15	0.35	0.08-1.54	1.5	2.6	0.25	0.48	0.12-1.85
$CD56^{dim}$ NK cells	7.8	1.1	0.91	1.08	0.28-4.14	2.6	1.1	0.97	0.98	0.25-3.76
NK cells	3.6	8.5	0.6	0.7	0.18-2.71	2.4	1.8	0.46	0.54	0.12-2.35
T cells	2.6	8.5	0.2	0.41	0.10-1.69	2.0	2.7	0.18	0.32	0.06-1.66
Th1 cells	7.8	1.5	0.94	1.06	0.26-4.37	2.6	1.5	0.73	1.23	0.30-5.01
Treg cells	3.6	14	0.62	0.70	0.17-2.90	2.4	1.7	1.0	1.01	0.25-4.08

HR, hazard ratio; 95% CI, 95% confidence interval; NK, natural killer; Treg, regulatory T; OS, overall survival, PFS, progression-free survival

Supplementary Table 5 | Content of the customized CheCUP targeted mutation panel. Gene names are provided with their Genbank transcript ID. In the Hs1 to Hs7 columns, the different hotspots within these genes are provided, indicated with the one-letter amino acid code and its position in the protein.

Gene	NM_ID	Hs1	Hs2	Hs3	Hs4	Hs5	Hs6	Hs7
AR	NM_000044	Q488						
ATM	NM_000051	W1795	E2052	R2486	R2714			
BCOR	NM_017745	R1183	Q1434	E1539				
BCORL1	NM_021946	V472	E1566					
BRAF	NM_004333	V600						
BRCA2	NM_000059	E475	N3272					
CDKN2A	NM_058195	G106						
CHEK2	NM_007194	T476						
ERCC2	NM_000400	E576						
FAT1	NM_005245	K2873						
FBXW7	NM_018315	S505						
GNAS	NM_000516	R201						
HLA-A	NM_002116	E152						
IDH1	NM_005896	R132						
JAK3	NM_000215	V722						
KDM6A	NM_021140	S537						
KMT2D	NM_003482	Q2337	Q3927					
KRAS	NM_004985	G12	Q61					
LATS2	NM_014572	E24						
MAP3K1	NM_005921	E1294						
NOTCH1	NM_017617	N918						
NOTCH2	NM_024408	Q767						
PBRM1	NM_018313	E356						
PIK3CA	NM_006218	E542	E545					
PLCG2	NM_002661	E530						
POLE	NM_006231	S1353	K1942					
RB1	NM_000321	p.spl? (3-4)						
RBM10	NM_005676	R171						
SDHA	NM_004168	R31						
SETBP1	NM_015559	S337						
SMAD3	NM_005902	Y237						
SMARCA4	NM_003072	R310	Q1236					
SMARCB1	NM_003073	S49						
SMO	NM_005631	I408						
SOX10	NM_006941	P363						
STK11	NM_000455	S31	R104	P281				
SUZ12	NM_015355	R196						
TERT	NM_198253	Promoter C250						
TET2	NM_001127208	W954	W1233					
TOP2A	NM_001067	H432						
TP53	NM_000546	Q38	G105	K132	E171	Q191	I195	M237
		All exons	S241	G245	R248	R273	R280	D281

Supplementary Note: Study protocol of the CheCUP trial

CLINICAL TRIAL PROTOCOL
CheCUP

EudraCT No. 2018-004562-33

CA209-8WY

A phase II, open-label, non-randomized, multi-center study evaluating the efficacy and safety of nivolumab plus ipilimumab in patients with cancer of unknown primary site who are relapsed after or refractory to platinum-based chemotherapy (CheCUP)

Phase of study: phase II, non-randomized, open-label, multi-center trial

GCP Statement: The study will be conducted in compliance with Good Clinical Practices (ICH-GCP) and the Declaration of Helsinki, and in accordance with applicable legal and regulatory requirements, including archiving of essential documents.

CONFIDENTIAL: This protocol contains confidential information and is intended solely for the guidance of the clinical investigation. This protocol may not be disclosed to parties not associated with the clinical investigation or used for any purpose without the prior written consent of the Principal Investigator/ Coordinating Investigator.

PROTOCOL APPROVAL SIGNATURE PAGE

The present trial protocol was subject to critical review and has been approved in the present version by the persons undersigned. The information contained is consistent with:

- the current risk-benefit assessment of the investigational medicinal product,
- the moral, ethical, and scientific principles governing clinical research as set out in the latest relevant version of Declaration of Helsinki, the principles of the guidelines of ICH Good Clinical Practices and the applicable legal and regulatory requirements.

The investigator will be supplied with details of any significant or new finding including AEs relating to treatment with the investigational medicinal product.

It will be ensured that the first subject is enrolled only after all ethical and regulatory requirements are fulfilled. Written consent from all subjects is received after detailed oral and written information and according to the requirements of local law (AMG). According to GCP-V §7, Section 2 No 15 it will be confirmed that all study participants will be informed on the type of encoding their personal data (pseudo-anonymization) and who receives or has access to such data. Subjects who do not agree to this data encoding and transfer will not be enrolled into the trial. In this context it will be assured (according to GCP-V §7, Section 3 No 15) that all investigational sites comply with the local regulatory requirements for data protection.

According to GCP-V §7, Section 3 No 4 the Sponsor/ Sponsor representative states that it is not planned to include subjects in a relationship of any dependence to the investigator or sponsor.

Via current versions of the clinical trial protocol and the investigator's brochure (IB) it will be ensured that all principal investigators are informed about the pharmacological-toxicologial assessments and results regarding the benefits and risks of the clinical trial.

Sensitive information censored

INVESTIGATOR SIGNATURE PAGE

I have read the above trial protocol and confirm that it contains all information to conduct the clinical trial. I pledge to conduct the clinical trial according to the protocol.

I will enroll the first subject only after all ethical and regulatory requirements are fulfilled. I will obtain written consent for trial participation from all subjects after detailed oral and written information and according to the requirements of local law (AMG). According to GCP-V §7, Section 2 No 15. I declare that all study participants will be informed on the type of encoding their personal data (pseudo-anonymisation) and who receives or has access to such data. Subjects who do not agree to this data encoding and transfer will not be enrolled into the trial. In this context I confirm (according to GCP-V §7, Section 3 No 15) that my investigational site complies with all local regulatory requirements for data protection.

Furthermore, I declare (according to GCP-V §7, Section 3 No 4) that to the best of my knowledge no subjects in a relationship of any dependence to the investigator or sponsor will be included.

I know the requirements for accurate notification of serious adverse events and I pledge to document and notify such events as described in the protocol.
I declare that I am informed about the pharmacological-toxicologial assessments and results regarding the benefits and risks of the clinical trial by reading the description in the clinical trial protocol and in the current version of the IB. I ensure that all investigators/ relevant staff at my site will be informed of this results and possibly new risks that are forwarded by the sponsor later on (e.g. via new version of the investigator's brochure.

I confirm that every staff will be adequately trained to guaranty compliance to the trial protocol incl. subsequent amendments.

I will retain all trial-related documents and source data as described. I will provide a Curriculum Vitae (CV) before trial start. I agree that the CV and Financial Disclosure (FD) may be submitted to the responsible EC.
As the clinical trial and the results have to be published in a clinical trial register and forwarded to the EC and competent authority. I agree that my name and clinic address will be part of this final trial (summary) report/ public register and are disclosed pursuant to §42b.

Date:	____________________	Signature:	____________________
		Name (Print name):	____________________
		Function:	Principal Investigator (PI)
		Investigational Site (address):	____________________<br><br>____________________<br><br>____________________
Date:	____________________	Signature:	____________________
		Name (Print Name):	____________________
		Function:	Deputy Principal Investigator

ADMINISTRATIVE STRUCTURE

Sensitive Information censored

Sensitive Information censored

Sensitive Information censored

TRANSLATIONAL RESEARCH

Liquid biopsy (cfDNA Samples)

Clinical Cooperation Unit Molecular Hematology/ Oncology

German Cancer Research Center

Michael Kirsch

Im Neuenheimer Feld 280

69120 Heidelberg

Phone: +49-6221 / 42-1443

Fax: +49-6221 / 42-1448

E-Mail: m.kirsch@dkfz-heidelberg.de

Microbiome Analysis

Division of Microbiome and Cancer

German Cancer Research Center

Dr. Christoph Stein-Thöringer

Im Neuenheimer Feld 280

69120 Heidelberg

Phone: +49-6221 / 42-4952

Fax: +49-6221 / 42-2995

E-Mail: c.stein-thoeringer@dkfz-heidelberg.de

PARTICIPATING SITES

The clinical trial is planned to be conducted up to 15 national trial sites. The sites will be specified in a separate document.

TABLE OF CONTENTS

PROTOCOL APPROVAL SIGNATURE PAGE **2**
INVESTIGATOR SIGNATURE PAGE **3**
ADMINISTRATIVE STRUCTURE **4**
PROTOCOL SYNOPSIS **12**
TRIAL SCHEDULE **20**
ABBREVIATIONS **22**
1 INTRODUCTION **25**
1.1 SCIENTIFIC BACKGROUND 25
1.2 TRIAL RATIONALE/ JUSTIFICATION 29
1.3 RISK-BENEFIT ASSESSMENT 31
1.4 DATA MONITORING COMMITTEE (DMC) 32
2 TRIAL OBJECTIVES AND ENDPOINTS **33**
2.1 PRIMARY OBJECTIVE AND PRIMARY ENDPOINT 33
3 TRIAL DESIGN AND DESCRIPTION **35**
3.1 TRIAL DESIGN 35
3.2 SCREENING PERIOD 35
3.3 TREATMENT PERIOD 38
3.4 POST-TREATMENT PERIOD 38
3.5 TRANSLATION RESEARCH 39
3.6 TRIAL DURATION AND SCHEDULE 39
4 SELECTION OF SUBJECTS AND CENTRES **40**
4.1 NUMBER OF SUBJECTS 40
4.2 TRIAL SITES 40
4.3 GENERAL CRITERIA FOR SUBJECTS' SELECTION 40
4.4 INCLUSION CRITERIA 40
4.5 EXCLUSION CRITERIA 43
4.6 CRITERIA FOR WITHDRAWAL 45
4.6.1 *Withdrawal of Subjects from Treatment* *45*
4.6.2 *Premature Closure of the Clinical Trial or a Trial Site* *47*
5 INVESTIGATIONAL MEDICINAL PRODUCTS (IMP) **48**
5.1 STUDY MEDICATION 48
5.1.1 *General information about Nivolumab* *48*
5.1.2 *General information about Ipilimumab* *48*
5.2 SUPPLY, PACKAGING AND LABELLING OF IMPS 48
5.3 SUPPLIES AND DRUG ACCOUNTABILITY 48
5.4 ADMINISTRATION OF STUDY MEDICATION 49
5.4.1 *Assignment of Identification Codes* *49*
5.4.2 *Dosage Schedule* *49*
5.4.3 *Compliance* *49*
5.4.4 *Prior and Concomitant Diseases* *50*
5.4.5 *Prior and Concomitant Medication* *50*
5.4.6 *Adjustments to dosage and delays in administration of nivolumab and ipilimumab in the individual trial subject* *51*
5.4.7 *Dosing delay and discontinuation criteria of study treatment* *51*
5.4.8 *Dosing discontinuation criteria requiring patient withdrawal from the study treatment* *56*
6 DESCRIPTION OF TRIAL VISITS **58**
6.1 SCREENING VISIT 58
6.2 COMBINATION THERAPY VISITS 59

6.3 SURVEILLANCE FOR IMMUNE-RELATED ADVERSE EVENTS (IRAES) 60
6.4 STAGING VISITS 61
6.5 END OF TREATMENT VISIT 61
6.6 SAFETY PHONE CALL 62
6.7 FOLLOW-UP PHASE 62
6.8 STUDY VISITS IN CASE OF TRIAL SITE IMPAIRMENT IN CASE OF A PANDEMIC 62

7 METHODS OF DATA COLLECTION 63

7.1 TMB VALUE ANALYSIS 63
7.2 MICROBIOME ANALYSIS 63
7.3 SAFETY PARAMETERS 64
7.3.1 *Adverse Events 64*
7.4 EFFICACY PARAMETERS 64
7.4.1 *Progression-free Survival (PFS) 64*
7.4.2 *Overall Survival (OS) 64*

8 ADVERSE EVENTS 65

8.1 DEFINITIONS 65
8.1.1 *Adverse Event 65*
8.1.2 *Serious Adverse Event 66*
8.1.3 *Adverse (Drug) Reaction 67*
8.1.4 *Expectedness 67*
8.1.5 *Suspected Unexpected Serious Adverse Reaction (SUSAR) 68*
8.1.6 *Relationship and Outcome of AEs 68*
8.2 TREATMENT OF AES / SAES 70
8.3 PERIOD OF OBSERVATION AND DOCUMENTATION 70
8.4 REPORTING OF SERIOUS ADVERSE EVENTS BY INVESTIGATOR 70
8.5 EXPEDITED REPORTING 70
8.6 SAFETY MANAGEMENT 71

9 STATISTICAL PROCEDURES 72

9.1 SAMPLE SIZE CALCULATION 72
9.2 ANALYSIS VARIABLES 72
9.3 DEFINITION OF TRIAL POPULATION TO BE ANALYZED 72
9.4 STATISTICAL METHODS 72
9.5 INTERIM ANALYSES 73
9.6 FINAL ANALYSIS 73

10 DATA MANAGEMENT 74

10.1 DATA COLLECTION 74
10.2 DATA HANDLING 74
10.3 ARCHIVING OF ESSENTIAL DOCUMENTS AND DATA 75

11 ETHICAL AND LEGAL ASPECTS 76

11.1 GOOD CLINICAL PRACTICE 76
11.2 LEGAL BASES 76
11.2.1 *Declaration of Helsinki 76*
11.2.2 *Other Legal Bases 76*
11.3 APPROVAL OF TRIAL PROTOCOL AND AMENDMENTS 76
11.4 NOTIFICATION OF REGULATORY AUTHORITIES 77
11.5 SUBJECT INFORMATION AND INFORMED CONSENT 77
11.6 INSURANCE 78
11.7 CONTINUOUS INFORMATION TO THE ETHICS COMMITTEE AND THE COMPETENT AUTHORITY 78

12 QUALITY CONTROL AND QUALITY ASSURANCE 79

12.1 DIRECT ACCESS TO SOURCE DOCUMENTS ACCORDING TO ICH-GCP 79
12.2 DATA PROTECTION 79
12.3 MONITORING 79

12.4 INSPECTIONS AND AUDITS ... 80
12.5 RESPONSIBILITIES OF THE INVESTIGATOR ... 80

13 ADMINISTRATIVE AGREEMENTS ... 81

13.1 FINANCING OF THE TRIAL ... 81
13.2 FINANCIAL DISCLOSURE ... 81
13.3 REPORTS ... 81
13.4 REGISTRATION OF THE TRIAL ... 81
13.5 PUBLICATION ... 81

15 REFERENCES ... 82

16 APPENDICES ... 86

TABLE INDEX

Table 1 Therapy for Patients With Favorable-Risk Cancer of Unknown Primary ... **27**

Table 2 Commonly Used Low-Toxicity Chemotherapy Regimens for Patients With Cancer of Unknown Primary in the non-specific subset ... **28**

Table 3 Objectives and Corresponding Endpoints ... **33**

Table 4 Definitions and types of premature study withdrawal ... **46**

Table 5 Doses and Infusion times for intravenous administration of nivolumab and ipilimumab **49**

Table 6 Discontinuation / Delay Criteria ... **52**

Table 7 Study visits 1st cycle ... **59**

Table 8 Study visits from 2nd cycle ... **60**

PROTOCOL SYNOPSIS

**TITLE**	***A phase II, open-label, non-randomized, multi-center study evaluating the efficacy and safety of nivolumab plus ipilimumab in patients with cancer of unknown primary site (CUP) who are relapsed after or refractory to platinum-based chemotherapy***
**SHORT TITLE**	Nivolumab/Ipilimumab in second line CUP-syndrome
**CLINICAL TRIAL CODE**	CheCUP
**EUDRACT NO.**	2018-004562-33
**INDICATION**	CUP-syndrome, relapsed/refractory to platinum-based chemotherapy<br>ICD10: C80.0
**OBJECTIVES**	Primary<br>To compare the efficacy of nivolumab plus ipilimumab in subjects with high (≥ 12 mutations/MB) vs. Intermediate/low (< 12 mutations/MB) TMB poor-prognosis CUP (non-specific subset) who are relapsed or refractory to platinum-based first-line chemotherapy<br><br>Secondary<br>To evaluate the efficacy of nivolumab plus ipilimumab in subjects with poor-prognosis CUP (non-specific subset) who are relapsed or refractory to platinum-based first-line chemotherapy
**PHASE**	II
**INVESTIGATIONAL MEDICINAL PRODUCT(S)**	Nivolumab and Ipilimumab
**REFERENCE DRUG**	N.A.
**STUDY DESIGN**	open-label
**STUDY POPULATION**	Key Inclusion Criteria<br>• Signed Informed Consent Form<br>• Able and willing to comply with the study protocol<br>• Age ≥ 18 years at time of signing Informed Consent Form<br>• Histologically-confirmed disseminated or advanced unresectable CUP diagnosed according the criteria defined in the 2015

	ESMO Clinical Practice Guidelines for CUP. Acceptable disease histology includes:<br>– Adenocarcinoma of unknown primary site (ACUP)<br>– Poorly differentiated adenocarcinoma of unknown primary site<br>– Poorly differentiated carcinoma of unknown primary site<br>– Squamous cell carcinoma of unknown primary site (SCUP)<br>• At least one lesion that is measurable according to RECIST v1.1 by CT/MRI<br>• Availability of a tumor FFPE block either fresh or archival if obtained ≤ 6 months at Screening that is sufficient for generation of a TruSight Oncology 500 (TSO500) panel at the central reference pathology laboratory or pre-existing result of a TMB analysis from routinely performed panel sequencing using the TSO500 panel at the MPZ, Institute of Pathology, University Heidelberg, which must not be older than ≤ 6 months at screening, respectively. In case one attempt to perform TMB analysis on a new specimen has failed due to insufficient tumor cell quantity or insufficient quality in the specimen, or a re-biopsy has failed or cannot be performed for clinical or technical reasons, resorting to a specimen not older ≤24 months is allowed as an exception.<br>• Availability of test reports confirming local CUP diagnosis. If test reports confirming local CUP diagnosis are not available, an FFPE block or a fresh biopsy sample must be submitted that is sufficient to allow for central confirmation of CUP diagnosis.<br>• Disease relapse or progression after at least three cycles of a platinum-based standard chemotherapy. There is no upper limit of prior treatments received.<br>• Subjects who have received prior surgery and/or radiotherapy and/or stereotactic brain metastasis radiosurgery are eligible. In case of prior radiotherapy, the measurable lesion(s) must not have been irradiated, radiotherapy has to be finished at least 7 days before start of study treatment and the patient must have

recovered to grade 1 or less from any toxicity of radiotherapy.

- ECOG performance status of 0 - 2
- Life expectancy ≥ 12 weeks
- Eligible for immune checkpoint inhibitor
- Adequate hematologic and end-organ function as detailed in the protocol (see section 4.4)
- For women of childbearing potential and men capable of reproduction: agreement to remain abstinent (refrain from heterosexual intercourse) or use contraceptive methods with a failure rate of $<$1% per year during the treatment period and for at least 5 months for women and 7 months for men, respectively after the last dose of study treatment.
- Recovery from significant toxicity from platinum-doublet therapy to Grade ≤ 1, except for alopecia and for neurosensory toxicity, which must be ≤ 2
- Recovery from active infections requiring intravenous antibiotics, with antibiotic therapy ceased for ≥ 7 days prior to planned start of therapy

Key Exclusion Criteria

- Subjects with any of the specific non-CUP neoplasms identified in the ESMO CUP guidelines (Fizazi et al. 2015)
- Subjects belonging to any of the following subsets of CUP with favorable prognoses:

– Poorly differentiated carcinoma with midline distribution

– Women with papillary adenocarcinoma of the peritoneal cavity

– Women with adenocarcinoma involving only the axillary lymph nodes

– Squamous cell carcinoma restricted to cervical lymph nodes

– Poorly and well differentiated neuroendocrine tumors

– Men with blastic bone metastases and elevated PSA

	– Subjects with a single, small tumor potentially resectable and/or amenable to radiotherapy with curative intent<br>– Colon cancer-type CUP<br>• Known presence of brain or spinal cord metastasis, as determined by CT or magnetic resonance imaging (MRI) evaluation during screening. As an exception, patients with brain metastases are allowed to be included if all of the following five criteria are met:<br>(i) the total number of brain metastases is 3 or less,<br>(ii) brain metastases were / are asymptomatic,<br>(iii) brain metastases have been completely surgically resected or completely treated with stereotactic radiosurgery<br>(iv) there was / is no indication for whole-brain irradiation,<br>(v) a brain MRI or high-resolution CT-scan at screening shows no evidence of residual disease.<br>If 1 to 3 asymptomatic brain metastases are detected at screening and are treatable and treated with stereotactic radiosurgery within the screening period, no renewed MRI / CT imaging of the brain is required before inclusion.<br>Benign lesions such as meningiomas may be accepted, if demonstration is made that they will not affect the interpretation of the study results or render the patient at high risk from treatment complications.<br>• History or known presence of leptomeningeal disease<br>• Uncontrolled or symptomatic hypercalcemia (serum calcium $\geq$ 2.9mmol/L)<br>• Known clinically significant history of liver disease consistent with Child-Pugh Class B or C, including active viral or other hepatitis, current alcohol abuse, or cirrhosis<br>• Human immunodeficiency virus (HIV) infection<br>• Positive for hepatitis C virus (HCV) infection at screening

	• Positive for hepatitis B surface antigen (HBsAg) at screening<br>• Active tuberculosis at Screening<br>• Significant cardiovascular disease (such as New York Heart Association Class II or greater cardiac disease, myocardial infarction, or cerebrovascular accident) within 3 months prior to initiation of study treatment, unstable arrhythmia (including active ventricular arrhythmia requiring medication), or unstable angina<br>• Major surgical procedure, other than for diagnosis, within 4 weeks prior to initiation of study treatment, or anticipation of need for a major surgical procedure during the study<br>• History of malignancy other than CUP within 5 years prior to screening, with the exception of malignancies with a negligible risk of metastasis or death (e.g., 5-year OS rate >90%), such as adequately treated carcinoma in situ of the cervix, non-melanoma skin carcinoma, localized prostate cancer, ductal carcinoma in situ, or stage I uterine cancer<br>• Solid organ transplantation<br>• Prior allogeneic stem cell transplantation with follow-up < 1 year, need for systemic immunosuppression or active chronic graft-versus host disease (cGVHD)<br>• Any other disease, metabolic dysfunction, physical examination finding, or clinical laboratory finding that contraindicates the use of an investigational drug, may affect the interpretation of the results, or may render the patient at high risk from treatment complications<br>• Known allergy or hypersensitivity to any component of the immunotherapy, including history of severe allergic anaphylactic reactions to chimeric or humanized antibodies or fusion proteins and to Chinese hamster ovary cell products or other recombinant human or humanized antibodies for nivolumab and ipilimumab.<br>• Subjects with an active autoimmune disease, including, but not limited to, myasthenia gravis, myositis, autoimmune hepatitis, myocarditis, systemic lupus erythematosus, rheumatoid arthritis, inflammatory bowel disease,

	antiphospholipid antibody syndrome, Wegener granulomatosis, Sjögren syndrome, Guillain-Barré syndrome, or multiple sclerosis. Subjects with type I diabetes mellitus, hypothyroidism only requiring hormone replacement, skin disorders (such as vitiligo, psoriasis, or alopecia) not requiring systemic treatment, or conditions not expected to recur in the absence of an external trigger are permitted to enroll.<br>• Subjects with a condition requiring systemic treatment with either corticosteroids (> 10 mg daily prednisone equivalents), or other immuno-suppressive medications within 14 days of study treatment. Inhaled or topical steroids and adrenal replacement doses > 10 mg daily prednisone equivalents in the absence of active autoimmune disease are permitted.<br>• Subjects who received prior treatment with an anti-PD-1, anti-PD-L1, anti-PD-L2, anti-CD137, or anti-CTLA-4 antibody, or any other antibody or drug specifically targeting T-cell co-stimulation or checkpoint pathways<br>• All toxicities attributed to prior anti-cancer therapy other than alopecia and fatigue must have resolved to Grade 1 (NCI CTCAE version 5) or baseline before administration of study drug. Subjects with toxicities attributed to prior anti-cancer therapy which are not expected to resolve and result in long lasting sequelae, such as neuropathy after platinum-based therapy, are permitted to enroll.<br>• Systemic treatment for cancer (any chemotherapy, biologics for cancer or investigational therapy) within 21 days of first administration of study treatment<br>• Radiotherapy or stereotactic brain metastasis radiosurgery has to be finished at least 7 days before inclusion into the study and the subject must have recovered to grade 1 or less from any toxicity of radiotherapy / stereotactic brain metastasis radiosurgery.<br>• Subjects must not have received a live / attenuated vaccine within 30 days of first treatment.<br>• Pregnancy or breastfeeding, or intention of becoming pregnant during study treatment or within 5 months after the last dose of study

	treatment or intention of fathering a child within 7 months after the last dose of study treatment.
**SAMPLE SIZE**	To be screened: 700<br>To be enrolled 194<br>(97 subjects with high and intermediate/low TMB, respectively)<br>To be analyzed: 194
**TRIAL DURATION**	Total trial duration: 36 months<br>Duration of clinical phase: 24 months<br>Beginning of the preparation phase: 10/2018<br>FSI (first subject in): 12/2019<br>LSI (last subject in): 12/2021<br>LSO (last subject out): 12/2022<br>DBL (database lock): [Q1 2023]<br>Statistical analyses completed: [Q2 2023]<br>Trial report completed: [Q2 2023]
**STATISTICAL ANALYSIS**	This is a non-randomized biomarker trial. Tumor mutational burden (TMB) is considered as biomarker. Subjects showing high TMB are considered biomarker-positive. A total of 194 subjects with 191 events are required to detect a hazard ratio of 0.65 for biomarker positive vs biomarker negative subjects with 80% power at the two-sided significance level of 5%. Median progression-free survival in the studied subject population is assumed to be 2.3 months, and 15% of subjects are expected to be biomarker-positive. Biomarker-positive subjects are expected to have a favorable prognosis. Assuming a hazard ratio of 0.65 for biomarker-positive versus biomarker-negative subjects and exponentially distributed survival, median survival times are 2.18 and 3.35 months for biomarker-negative and biomarker-positive subjects, respectively. Subjects will be recruited in a 1:1 ratio, i.e. biomarker-positive subjects will be enriched, which means that approximately 700 subjects need to be assessed for their TMB status. There will be a 24 months recruitment period and a minimal follow-up time of 12 months. The primary analysis will be performed by testing the null hypothesis of no difference in PFS between both biomarker groups using a log-rank test at a significance level of 5%.

	Primary endpoint:<br>• Progression-free survival (PFS)<br><br>Secondary endpoints:<br>• Overall survival (OS)<br>• Overall response rate (ORR)<br>• Duration of clinical benefit (DCB)<br><br>Safety endpoints:<br>• Incidence, nature and severity of adverse events (AEs)<br>• Incidence and reasons for any dose reductions, interruptions, or premature discontinuation of any component of study treatment<br>• Clinically significant laboratory values and vital signs
**NUMBER OF TRIAL SITES**	Approximately 15
**FINANCING**	Bristol-Myers Squibb (BMS)
**TRANSLATIONAL RESEARCH**	cfDNA serial plasma samples ("liquid biopsies") will be collected at screening, at staging visits and on the EoT visit from those subjects who consent to do so. These samples will be analysed (i) by panel sequencing in order to detect newly developed mutations as sign of tumor evolution and (ii) by digital PCR to quantify mutations in order to monitor treatment response and progression.<br><br>One saliva sample and one stool sample will be collected at screening from those subjects who consented to do so. The microbiome of these samples will be analysed by sequencing in order to determine bacterial flora associated with treatment response as potential predictive marker.

TRIAL SCHEDULE

Visit No.	Visit 1	Visit 2	Visit 3	Visit 4	Visit 5	Visit 6	Visit …		Visit n	EoT Visit	Safety F/U	Survival Contact
Assessments	Screening	Treatment Phase									Phone call	Follow-up
		Start							End			
Relative timepoint	-42- 0 days	day 1	day 8# (+/-1)	day 15* (+/-2)	day 22# (+/-2)	day 29* (+/-2)	day 36* (+/-2)	every 12 weeks (+/-10 days)≠		day 30 (+/-7) after last study treatment	day 100 (+/-7) after last study treatment	every 3(+/-1) months
Inclusion/ exclusion criteria	●											
Tumor tissue sample for panel seq+	●											
cfDNA blood sample (Streck, 20 ml; whole blood EDTA, 10 ml; serum sample, 10 ml)	●							●		●		
stool and/or saliva sample for translational research	●											
Written informed consent	●											
Physical examination	●	●	●	●	●	●	●	●	●	●		
Medical history	●											
Pregnancy test (females of childbearing potential only)	●¥	●¥				●¥						

Visit No.	Visit 1	Visit 2	Visit 3	Visit 4	Visit 5	Visit 6	Visit …		Visit n	EoT Visit	Safety F/U	Survival Contact
Assessments	Screening	Treatment Phase									Phone call	Follow-up
		Start							End			
Relative timepoint	-42 – 0 days	day 1	day 8# (+/-1)	day 15* (+/-2)	day 22# (+/-2)	day 29* (+/-2)	day 36* (+/-2)	every 12 weeks (+/-10 days)≠		day 30 (+/-7) after last study treatment	day 100 (+/-7) after last study treatment	every 3(+/-1) months
12 lead ECG	•							•				
left ventricular ejection fraction	•											
HIV, HBV, HCV serology; tubrtculosis, if indicated	•											
Total Laboratory	•	•	•	•	•	•	•	•	•	•		
Tumor assessment (CT-/MRI scan)	•							•		• (if required)		
IMP administration*		Nivo, Ipili		Nivo		Nivo						
ECOG	•	•	•	•	•	•	•	•	•	•		
vital signs**	•	•	•	•	•	•	•	•	•	•		
Concomitant medications	•	•	•	•	•	•	•	•	•	•		
AE documentation		•	•	•	•	•	•	•	•	•	•	
Phone call											•	•

* Interval between two nivolumab (Nivo) infusions must not be less than 12 days; Ipilimumab (Ipili), blood results have to available before IMP administration
** Vital signs (weight, blood pressure, pulse, body temperature)
# skipped in subsequent cycles e.g. cycle 2
+ To assess the subject's eligibility either an already pre-existing FFPE block or a fresh FFPE block has to be analyzed during the screening phase. Alternatively, the TMB value from a routinely performed panel sequencing can be used.
¥ A highly sensitive serum pregnancy test must be carried out within 3 days prior to the initial dose of study treatment. Additionally, a highly sensitive serum pregnancy test has to be done on day29 of the first cycle. In subsequent cycles, the highly sensitive pregnancy test has to be done one day before or on day 1 of each cycle.
≠ should correspond to day 29 – 36 of every second cycle

ABBREVIATIONS

5-FU	5-fluorouracil
ACUP	Adenocarcinoma of unknown primary site
AE	Adverse Event
ALT	Alanine transaminase
AMG	German Drug Law (Deutsches Arzneimittelgesetz)
ANC	Absolute neutrophil count
Anti-CD137	Antibody against the protein cluster of differentiation 137
aPTT	Activated partial thromboplastin time
AST	Aspartate transaminase
ATC	Anatomical-Therapeutic-Chemical Code, part of WHO-DRL (Drug Reference List)
AUC	Area under the concentration-time curve
BDSG	Bundesdatenschutzgesetz
BML	Below measurable limit
BMS	Bristol-Myers Squibb
BDSG	Bundesdatenschutzgesetz
CDX2	Caudal-type homeobox transcription factor 2
cfDNA	circulating free DNA
CHO	Chinese hamster ovary
CK	Cytokeratin
CP	Conditional power
CR	Complete Response
CRF	Case Report Form
CRP	C-reactive protein
CT	Computed Tomography
CTCAE v5.0	Common Terminology Criteria for Adverse Events version 5.0
CTLA-4	Cytotoxic T-Lymphocyte Antigen 4
CUP	Cancer of unknown primary site
CV	Curriculum Vitae
DBL	Data Base Lock
DCB	Duration of clinical benefit
DILI	Drug-induced liver injury
DKFZ	Deutsches Krebsforschungszentrum
DM	Data Manager
DMC	Data Monitoring Committee
DNA	Deoxyribonucleic acid
DSUR	Development Safety Update Report
DVP	Data validation plan
EC	Ethics Committee
ECG	Electrocardiography
eCRF	Electronic case report form

ECOG	Eastern Cooperative Oncology Group
EMA	European Medicines Agency
ESMO	European Society for Medical Oncology
EoT	End of Treatment Visit
FAS	Full-analysis set
FD	Financial Disclosure
FDA	Food and Drug Administration
FFPE	Formalin-fixed paraffin-embedded tissue
FSI	First Subject In
FPI	First Subject In
HBcAb	Hepatitis B core antibody
HBV	Hepatitis B virus
HBsAg	HBV surface antigen
HCV	Hepatitis C virus
HIV	Human immunodeficiency virus
GCP	Good Clinical Practice
GCP-V	Good Clinical Practice Ordinance (GCP-Verordnung)
GDPR	General Data Protection Regulation
IB	Investigator´s Brochure
ICD-10	International statistical Classification of Diseases and related health problems, 10th revision
ICH	International Council on Harmonisation of Technical Requirements for Registration of Pharmaceuticals for Human Use
ICH-GCP	Integrated addendum R2 of ICH harmonised tripartite guideline on GCP
ICMJE	International Committee of Medical Journal Editors
IHC	Immunohistochemistry
IMP	Investigational Medicinal Product
IMPD	Investigational Medicinal Product Dossier
INN	International Nonproprietary Name
INR	International normalized ratio
Ipili	Ipilimumab
irAE	Immune-related adverse event
ISF	Investigator Site File
ISRCTN	International Standard Randomised Controlled Trial Number
i.v.	Intravenous/-ly
IZKS	Interdisziplinäres Zentrum Klinische Studien
KKS	Coordination Centre for Clinical Trials (Koordinierungszentrum für Klinische Studien)
LDH	Lactate dehydrogenase
LKP	Coordinating Investigator according to AMG (Leiter der Klinischen Prüfung)
LSO	Last Subject Out
LSI	Last Subject In

LSO	Last Subject Out
Mb	Mega base
MedDRA	Medical Dictionary for Regulatory Activities
MPZ	Molekularpathologisches Zentrum, Institute of Pathology, University Hospital Heidelberg
MRI	Magnetic Resonance Imaging
NCI	National Cancer Institute
NE	Not evaluable
Nivo	Nivolumab
NSCLC	Non-small cell lung cancer
ORR	Objective response rate
OS	Overall survival
PCR	Polymerase chain reaction
PD	Progressive disease
PD-1	Programmed Death-1
PD-L1	Programmed Death-Ligand 1
PD-L2	Programmed Death-Ligand 2
PEI	Paul-Ehrlich-Institute
PET	Positron emission tomography
PFS	Progression-free survival
PI	Principal Investigator
PR	Partial response
PSA	Prostate-specific antigen
PV	Pharmacovigilance
Q	Quarter (time span)
q3w	Once every 3 weeks
RECIST	Response Evaluation Criteria in Solid Tumors
RDE	Remote data entry
RT	Radiotherapy
SAE	Serious Adverse Event
SC	Steering Committee
SCUP	Squamous cell carcinoma of unknown primary site
SD	stable disease
SmPC	Summary of Product Characteristics
SOP	Standard Operating Procedure
SUSAR	Suspected Unexpected Serious Adverse Reaction
ULN	Upper Limit of Normal
TMB	Tumor mutational burden
TMF	Trial Master File
TSH	Thyroid stimulating homone
TSO500	TruSight Oncology 500
TTF1	Thyroid Transcription Factor 1
WBC	White blood cell

1 INTRODUCTION

1.1 Scientific Background

Cancer of unknown primary site (CUP) is defined as a histologically-confirmed metastatic cancer for which a standardized diagnostic work-up fails to identify the site of origin at the time of diagnosis (Pavlidis and Fizazi 2009; Massard et al. 2011; Fizazi et al. 2015). A standardized diagnostic work-up in this context includes mainly:

- Histopathological review of biopsy material using immunohistochemistry (IHC)
- Detailed medical history of the subject
- Complete physical examination (including pelvic and rectal examination)
- Full blood count and biochemistry analysis
- Urinalysis and fecal occult blood tests
- Computed tomography (CT) scan of the thorax, abdomen and pelvis
- Mammography scan and breast MRI (in certain cases)

CUP accounts for 3% to 5% of all malignancies (Fizazi et al. 2015). The disease has a median age of occurrence of approximately 60 years, is rare in children, and is marginally more frequent in males. Survival of patients with CUP is poor, with a median overall survival (OS) of 8–11 months and a one-year survival rate of 25% (Massard et al. 2011).

No obvious risk factors have been identified for CUP (Pavlidis and Fizazi 2009; Massard et al. 2011; Fizazi et al. 2015). By definition, early detection of patients with CUP is problematic, and screening programs for the disease are nonexistent. Autopsies are performed in only a minority of patients with CUP, but even thorough post-mortem evaluations identify only 55–85% of the primaries, usually small asymptomatic tumors in the pancreas, lung, gut, and kidney (Abrams et al. 1950; Didolkar et al. 1977; Jordan and Shildt 1985; Le Chevalier et al. 1988; Mayordomo et al. 1993; Jemal et al. 2008).

Two opposing hypotheses have been proposed for CUP pathogenesis (Busson et al. 2006; Pentheroudakis et al. 2007). One hypothesis considers CUP to be a distinct biological entity with unique molecular features that accounts for the absence of the primary site and for early metastatic disease. The second hypothesis posits that CUP arises from different groups of unrelated tumors, each with a primary site that escapes detection.

The heterogeneity of CUP tumors as well as their lack of an identified tissue of origin impose challenges on how the disease is treated. The European Society for Medical Oncology (ESMO) has developed a treatment algorithm for CUP that consists of two general steps:

- In the first step, if examination results including clinical features, immunohistochemistry, radiology, laboratory values and additional diagnostic measures beyond those of the standard diagnostic work-up strongly suggest a tissue of origin, treatment is initiated based on established site-specific therapies for the identified cancer type. In particular, distinct clinic-pathological subgroups mimicking other malignancies, which are well defined as favorable-risk entities, should be recognized and treatment should be adjusted to the presumed primary.

- In the second step, patients for whom a likely tissue of origin cannot be identified and for whom local treatment in curative intent is not feasible are classified as poor-risk CUP and subsequent chemotherapeutic treatment should be initiated based on this classification.

To further evaluate the potential tissue of origin of CUP, as well as to exclude chemosensitive and potentially curable tumors (e.g., lymphomas and germ-cell tumors), high-quality tumor samples are subjected to extensive IHC on multiple antigenic markers (Abbruzzese et al. 1995; Oien 2009). Patients with metastatic lesions with site-specific IHC profiles may be considered for treatment with standard site-specific regimens adjusted to the likely primary tissue of origin if the morphology and clinical picture are also pointing in this direction.

In patients without a confirmed tissue of origin, two distinct subsets of CUP have been identified: favorable-risk CUP, and poor-risk CUP.

Favorable-Risk Cancer of Unknown Primary Site

A minority (15%–20%) of patients with CUP, as defined by clinical and pathological criteria (Table 1), are referred to as having favorable-risk disease (Fizazi et al. 2015). Within this subset, 30%–60% of patients can achieve long-term disease control if managed similarly to patients with a potentially equivalent metastatic cancer of known primary site. Retrospective analyses confirm that the clinical behavior, biology, response to treatment and outcome of subjects with favorable-risk CUP parallel those observed with metastatic tumors from a known primary site (Hainsworth and Fizazi 2009; Pavlidis et al. 2009; Spigel et al. 2009; Pentheroudakis et al. 2010; Pentheroudakis and Pavlidis 2010).

Table 1 Therapy for Patients With Favorable-Risk Cancer of Unknown Primary

CUP Subtype	Treatment	Potential Equivalent Tumor
Poorly differentiated neuroendocrine carcinomas of an unknown primary	Platinum + etoposide combination chemotherapy	Poorly differentiated neuroendocrine carcinomas with a known primary
Well-differentiated neuroendocrine tumor of unknown primary	Somatostatin analogs, streptozocin +5,-FU, sunitinib, everolimus	Well-differentiated neuroendocrine tumor of a known primary site
Peritoneal adenocarcinomatosis of a serous papillary histological type in females	Optimal surgical debulking followed by platinum–taxane-based chemotherapy	Ovarian cancer
Isolated axillary nodal metastases in females	Axillary nodal dissection mastectomy or breast irradiation and adjuvant chemo/hormone therapy	Breast cancer (found in 50%–70% when breast MRI is performed)
Squamous cell carcinoma involving nonsupraclavicular cervical lymph nodes	Neck dissection and/or irradiation of bilateral neck and head–neck axis. For advanced stages, induction chemotherapy with platinum-based combination or chemoradiation	Head and neck squamous cell cancer
CUP with a colorectal IHC (CK20 + CDX2 + CK7−) or molecular profile	Systemic treatment used for colorectal cancer	Metastatic colorectal cancer
Single metastatic deposit from unknown primary	Resection and/or RT ± systemic therapy	Single metastasis
Men with blastic bone metastases or IHC/serum PSA expression	Androgen deprivation therapy ± RT	Prostate cancer
Men with poorly differentiated carcinoma with midline distribution (extragonadal germ cell syndrome)	Platinum-based combination chemotherapy	Extragonadal germ cell tumor

5-FU, 5-fluorouracil; MRI, magnetic resonance imaging; IHC, immunohistochemistry; PSA, prostate-specific antigen; RT, radiotherapy; CK, cytokeratin

From (Fizazi et al. 2015).

Poor-Risk Cancer of Unknown Primary

The remaining patients (80%–85%) have more limited sensitivity to therapy. Two prognostic groups can be identified among patients with poor-risk disease based on the following criteria (Culine et al. 2002):

- Good performance status (ECOG 0-1) and a normal lactate dehydrogenase (LDH) level
- Poor performance status, elevated serum LDH, or both

The first prognostic group has a median OS of 1 year, while the second prognostic group has a median OS of ~4 months.

A review conducted in 2009 found that poor-risk CUP had similar outcome whether treatment was based on platinum salts, taxanes or new-generation cytotoxic compounds (gemcitabine, vinca-alkaloids or irinotecan) (Golfinopoulos et al. 2009). Hence, no single broad-based chemotherapy regimen has yet been identified as superior in this patient population. Importantly, superiority of any chemotherapy regimen over best supportive care only has never been formally demonstrated either. Nevertheless, platinum-based doublet chemotherapy regimens are viewed as standard-of-care in the first-line treatment of poor-risk CUP. Commonly used chemotherapy regimens for poor risk CUP are shown in Table 2.

Table 2 Commonly Used Low-Toxicity Chemotherapy Regimens for Patients With Cancer of Unknown Primary in the non-specific subset

Chemotherapy (mg/m^2)	Time	Interval	Comments
Cisplatin 60–75 +<br>Gemcitabine 1000	Day 1<br>Day 1+8	q3w	Fit patients, adequate hydration
Cisplatin 75 +<br>Etoposide 100	Day 1<br>Day 1–3	q3w	Fit patients with neuroendocrine-feature CUP, adequate hydration
Paclitaxel 175 +<br>Carboplatin AUC 5	Day 1	q3w	Convenient outpatient regimen, monitor neurotoxicity
Docetaxel 75 +<br>Carboplatin AUC 5	Day 1	q3w	Convenient outpatient regimen, monitor neurotoxicity
Irinotecan 160 +<br>Oxaliplatin 80	Day 1	q3w	Outpatient regimen, monitor for neurotoxicity and diarrhea
Oral capecitabine 2000 ±<br>Oxaliplatin 85–130	Days 1–14<br>Day 1	q3w	Outpatient regimen risk for diarrhea and neurotoxicity
Gemcitabine 1000/irinotecan 100	Day 1+8	q3w	Convenient outpatient regimen, monitor diarrhea

AUC, area under the concentration-time curve; q3w, once every 3 weeks

From (Fizazi et al. 2015)

Second-line chemotherapy regimens have never been systematically evaluated and only very few studies have been published on this topic (Culine et al. 2001; Hainsworth et al. 2001; Hainsworth et al. 2005; Möller et al. 2010). Derived from these data, second-line chemotherapy regimens containing gemcitabine, irinotecan, oxaliplatin, capecitabine or 5-fluorouracil lead to a progression-free survival (PFS) and overall survival (OS) of approximately 2.3 and 3.9 months, respectively, in poor risk CUP syndrome.

In addition to chemotherapy, CUP manifestations that pose localized problems can be irradiated or treated with other loco-regional therapy for pain relief and/or prevention of complications.

Recent data demonstrate that about 10% of patients with adeno/undifferentiated and 23% of patients with squamous cell CUP syndrome show high tumor mutational burden (TMB) levels

above 20 mutations/Mb (Gay et al. 2017; Krämer et al. 2018). In addition, several case reports suggest that subjects with CUP syndrome may respond favorably to immune checkpoint inhibitor treatment (Gröschel et al. 2016)

1.2 Trial Rationale/ Justification

Treatment of cancer is generally based on the specific neoplasm's tissue of origin (National Comprehensive Cancer Network), an approach that is obviously problematic in patients with CUP. ESMO recommends standard broad-based chemotherapeutic agents for non-specific subsets of CUP, but these regimens are suboptimal in poor-prognosis disease, resulting in median OS values of 1 year in patients with good ECOG performance status (0–1) and normal LDH and approximately 4 months in patients with poor ECOG performance status (2–4), elevated LDH, or both (Fizazi et al. 2015). Whether these chemotherapy regimens prolong survival over best supportive care remains unknown. With second-line chemotherapy PFS and OS are only approximately 2.3 and 3.9 months, respectively. The reason(s) for the low response remains unknown, but might reflect the fact that any case of CUP could originate from multiple types of primary lesions (each with its own susceptibility to specific anticancer agents) or that some other unique feature(s) of CUP biology blocks response. In either case, it is clear that a high unmet need exists for new therapeutic approaches in patients with CUP who do not fall into the favorable risk subset.

Immuno-oncology is a rapidly emerging approach for cancer treatment (Marshall and Djamgoz 2018). Immune checkpoint inhibitors block inhibitory receptors expressed on T lymphocytes (PD-1, CTLA-4) or their corresponding ligands expressed on tumor cells (PD-L1). Therapeutically blocking these inhibitory molecular axes using specific monoclonal antibodies targeting PD-1 (nivolumab) or CTLA-4 (ipilimumab), either as monotherapy or in combination, activates the immune system to recognize and target cancer cells via a T-cell-mediated immune response. Many recent clinical trials on immunotherapy have shown impressive results in many different advanced metastatic cancers including melanoma, non-small-cell lung cancer, renal cell carcinoma, head and neck squamous cell carcinoma, urothelial cancer, refractory Hodgkin's lymphoma, and malignancies with microsatellite instability, and have led to fast-track approval of immune checkpoint inhibitors for several cancer types by both the US Food and Drug Administration (FDA) and the European Medicines Agency (EMA). Importantly, in 2017 the FDA granted approval for the use of an immune checkpoint inhibitor for mismatch repair-deficient tumors, marking the first tissue-agnostic and site-agnostic drug approval by the agency (Le et al. 2017). For lung cancer, melanoma, urothelial cancer and many other types of neoplasms, this immuno-oncology approach has revolutionized treatment, resulting in clinically significant improvements in multiple clinical outcomes, including OS.

Given the therapeutic challenges imposed when the tissue of origin is unknown, CUP would appear to be especially well suited for an immuno-oncology treatment approach. Indeed, beyond the theoretical underpinning described above, an increasing amount of published data suggests that such an approach may have important clinical benefits in patients with CUP, especially in patients with higher levels of tumor mutational burden (TMB):

- Rizvi et al. found that higher TMB was associated with improved objective response rate (ORR), durable clinical benefit, and progression-free survival (PFS) in subjects with NSCLC who were treated with the PD-1 inhibitor pembrolizumab (Rizvi et al. 2015). Efficacy also correlated with the molecular smoking signature, higher neoantigen burden and DNA repair pathway mutations, each of which was associated with higher TMB. These results are consistent with the idea that newly formed, immunogenic neoantigens on tumor cells may stimulate antitumor response via cytotoxic T-lymphocytic killing. In further support of this interpretation, other recent studies demonstrated that anti-PD-1 therapy is effective in subjects with metastatic DNA mismatch repair-deficient or microsatellite instability-high colorectal cancer (Overman et al. 2017).

- First-line combination treatment with nivolumab and ipilimumab led to a significantly increased PFS in subjects with non-small-cell lung cancer and high tumor mutational burden as compared to standard chemotherapy (Hellmann et al. 2018). Similarly, neoadjuvant nivolumab was associated with major pathological responses in 45% of resectable non-small-cell lung cancers, with tumor mutational burden being predictive of the pathological response to PD-1 blockade (Forde et al. 2018).

- Other studies have shown that CUP is often associated with high TMB. For instance, Gay et al. assessed TMB in 6116 CUP tumor specimens, defining high, intermediate and low TMB as ≥ 20, ≥ 6 and < 20, and < 6 mutations/Mb, respectively (Gay et al. 2017). Significant numbers of patients within each tested tumor type (ACUP, CUP not otherwise specified [NOS], squamous cell CUP) had high TMB. Overall, 23% of squamous cell tumors, 15% of malignant neoplasm NOS tumors and 8-11% of ACUP or CUP, the most common tumors, had high TMB. In addition, 1.6% of CUP cases were found to be MSI-H. In line with these findings, and in support of a potential role for immunotherapy in CUP, Varghese et al. found that about 10% of CUP tumors harbor signatures of tobacco-related or ultraviolet-induced DNA damage and high TMB in 14% of cases (Varghese et al. 2017).

- In a recent case report, Gröschel and coworkers have described a major and long-lasting response of a patient with undifferentiated adeno-CUP syndrome refractory to both radio- and chemotherapy (Gröschel et al. 2016).

Combined, the above considerations clearly suggest that CUP patients might benefit from immunotherapy, but prospective clinical studies evaluating this potentially promising approach are lacking. Therefore, to test this hypothesis, the CheCUP study will directly assess the effect of a combination immune checkpoint inhibitor treatment with nivolumab and ipilimumab in subjects with poor-prognosis CUP (non-specific subset as defined in the ESMO guidelines for CUP (Fizazi et al. 2015)) who are resistant or refractory to platinum-based first-line chemotherapy.

Although within the study all subjects will receive the same treatment combination of nivolumab and ipilimumab, subjects will be stratified according to their tumor mutational burden (TMB), in order to determine whether TMB qualifies to predict response to treatment and overall survival of subjects with platinum-resistant/refractory poor-prognosis CUP.

Assessment of tumor mutational burden will be performed at the MPZ, Institute of Pathology, University of Heidelberg.

The combination of nivolumab plus ipilimumab has been tested successfully throughout numerous malignancies, including malignant melanoma (Wolchok et al. 2017), non-small cell lung cancer (Hellmann et al. 2017; Hellmann et al. 2018), small cell lung cancer (Antonia et al. 2016), and renal cell cancer (Motzer et al. 2018). Meanwhile, the combination of nivolumab plus ipilimumab is established as standard of care in patients with advanced melanoma. (Wolchok et al. 2017). In non-small cell lung cancer, an objective response rate (ORR) of 38% was obtained for the nivolumab 3 mg/kg, 2-weekly and ipilimumab 1 mg/kg, 6-weekly arm (Hellmann et al. 2017). In non-small cell lung cancer patients with a high TMB, the ORR with nivolumab plus ipilimumab was 45.3% versus 26.9% with chemotherapy, irrespective of the PD-L1 expression level (Hellmann et al. 2018). Likewise, in previously untreated renal cell carcinoma patients, nivolumab plus ipilimumab achieved superior remission rates as compared to the kinase inhibitor sunitinib (Motzer et al. 2018).

Regarding safety, results from a non-small cell lung cancer phase 1 trial showed that treatment with nivolumab and ipilimumab is tolerable (Hellmann et al. 2017). In this trial, the nivolumab 3 mg/kg, 2-weekly plus ipilimumab 1 mg/kg, 6-weekly schedule proved safe and tolerable with treatment-related grade 3 and 4 adverse events reported in 33% of patients. This marked a notable improvement in safety as compared to previous dosing schedules. In a later study by Hellmann et al., any grade 3 and 4 events were reported in 31.2% of patients, and treatment had to be discontinued due to treatment-related events in 17.4% of patients (Hellmann et al. 2018). In a small cell lung cancer trial, treatment-related grade 3 and 4 adverse events were observed in 19% of patients in the nivolumab 3 mg/kg plus ipilimumab 1 mg/kg arm, and 7% of patients allocated to this arm had to discontinue study treatment due to treatment-related adverse events. Accordingly, the safety profile was deemed manageable.

In conclusion, there is broad evidence from clinical cancer trials that the combination of nivolumab and ipilimumab is effective in a broad spectrum of cancer patients. There is also reliable data that the safety profile of nivolumab plus ipilimumab is favorable. With the 240 mg nivolumab flat dose, 2-weekly (which has been shown to be equivalent to nivolumab 3 mg/kg, 2 weekly (Checkmate 817, 2018; Hellmann et al. 2018)) plus ipilimumab 1 mg/kg, 6-weekly schedule that will be used in this trial we have adopted a dosing scheme well established in lung cancer patients – who should be comparable from a clinical perspective to the CUP target population of this trial – and with a proven favorable safety profile.

1.3 Risk-benefit Assessment

As described in Section 1, patients with CUP (non-specific subset) have poor outcomes on currently recommended treatments and, hence, have a high unmet need for new therapeutic approaches. Unfortunately, treatment options for CUP have not evolved in decades, and no drug has yet been registered specifically for the disease. This dearth of treatment options likely reflects the fact that, until recently, treatment of cancer has generally been based on the neoplasm's tissue of origin, which is obviously problematic in the case of CUP. However, with the advent of large-scale DNA sequencing technologies, and the availability of immunotherapies, a new treatment paradigm may now be possible for CUP that is independent of tissue of origin (see above, Study Rationale). The potential benefits of this treatment approach provide a strong medical rationale for carrying out the current study.

While combination immunotherapy with nivolumab and ipilimumab is not established for CUP, both drugs have been approved for the treatment of several other cancers. As immune checkpoint inhibition has been shown to operate at the molecular level in a similar fashion across different cell types, it is reasonable to hypothesize that the immunotherapy regimen used in this study may have significant benefit in patients with CUP. Moreover, tumor types with proven benefit from immune checkpoint inhibitor therapy comprise a significant fraction of the primary tumor spectrum responsible for CUP (Pentheroudakis et al. 2007).

An additional advantage of using agents approved in other cancer indications is that their safety profiles have been assessed in great detail, at least in other cancers (Postow et al. 2018). Several steps will be taken to further limit the risk of participants in this study. First, administration of both compounds will be performed in a setting with emergency medical facilities and staff who are trained to monitor for and respond to medical emergencies. Second, identified and potential risks associated with nivolumab and ipilimumab will be closely monitored throughout this study. Third, the study will have an independent data monitoring committee (DMC) to assess benefit-risk profiles and safety signals. Fourth, key IMP-specific eligibility criteria relating to safety in other cancers will be assessed prior to starting treatment. Finally, the study contains protocol-specified drug interruption criteria designed to ensure safety.

In view of the biological and medical rationale for using immunotherapies in subjects with CUP—and with the above safety precautions in place—a favorable benefit/risk proposition exists to support the conduct of this study. This is especially true given the high unmet need for new therapeutic options in patients who have this difficult-to-treat disease.

1.4 Data Monitoring Committee (DMC)

Ensuring the ethical conduct of the trial and protecting the rights and welfare of the subjects are the tasks of the DMC. The DMC consists of two clinical experts on carcinoma of unknown primary. Since an interim analysis is planned, in addition an external biometrician is involved in the DMC. Neither clinical experts nor the statistician are involved in the conduct of the trial. The DMC will meet on a regular basis (approx. every six months). After reviewing the data on the study conduct (recruitment, protocol adherence/ protocol deviations) and on safety issues, the DMC will make recommendations on the further study conduct (modification, continuation, closure).

Throughout this process of surveillance, the DMC provides the sponsor with recommendations with regard to continuing the trial (e.g. termination or modification) based on the data collected. The data necessary for the DMC to fulfil this function are provided by the sponsor as determined by the DMC. Amongst other datasets, these must include listings providing information on serious adverse events and further variables that the DMC considers necessary at least every 6 months and when formal interim analyses are conducted.

2 TRIAL OBJECTIVES AND ENDPOINTS

2.1 Primary Objective and Primary Endpoint

The main purpose of the study is to determine the efficacy and safety of an immunotherapy with nivolumab plus ipilimumab in subjects with poor-prognosis CUP (non-specific subset as defined in the ESMO guidelines (Fizazi et al. 2015)) who are resistant or refractory to platinum-based first-line chemotherapy. Subjects will be stratified according to their tumor mutational burden (TMB).

Specific objectives and corresponding endpoints for the study are outlined in Table 3.

Table 3 Objectives and Corresponding Endpoints

**Primary Efficacy Objective**	**Corresponding Endpoint**
To compare the efficacy of nivolumab plus ipilimumab in subjects with high vs. Intermediate/low TMB poor-prognosis CUP (non-specific subset) who are resistant or refractory to platinum-based first-line chemotherapy	Progression-free survival (PFS), defined as the time from treatment start to the first occurrence of disease progression, as assessed by the investigator according to Response Evaluation Criteria in Solid Tumors, Version 1.1 (RECIST v1.1), or death from any cause, whichever occurs first.
**Secondary Efficacy Objectives**	**Corresponding Endpoints**
• To evaluate the efficacy of nivolumab plus ipilimumab in subjects with poor-prognosis CUP (non-specific subset) who are resistant or refractory to platinum-based first-line chemotherapy	• Progression-free survival (PFS), defined as the time from treatment start to the first occurrence of disease progression, as assessed by the investigator according to Response Evaluation Criteria in Solid Tumors, Version 1.1 (RECIST v1.1), or death from any cause, whichever occurs first<br>• Overall survival (OS), defined as the time from treatment start to death from any cause<br>• Overall response rate (ORR), defined as the proportion of subjects who exhibit a CR or PR to study treatment on two consecutive occasions ≥ 4 weeks apart<br>• Duration of clinical benefit (DCB), defined as the time from the first occurrence of a CR, PR or SD after treatment start until disease progression or death from any cause, whichever occurs first<br>Responses will be determined by the investigator according to RECIST v1.1

• To compare the efficacy of nivolumab plus ipilimumab in subjects with high vs. Intermediate/low TMB poor-prognosis CUP (non-specific subset) who are resistant or refractory to platinum-based first-line chemotherapy	• Overall survival (OS), defined as the time from treatment start to death from any cause<br>• Overall response rate (ORR), defined as the proportion of subjects who exhibit a CR or PR to study treatment on two consecutive occasions ≥ 4 weeks apart<br>• Duration of clinical benefit (DCB), defined as the time from the first occurrence of a CR, PR or SD after treatment start until disease progression or death from any cause, whichever occurs first<br>Responses will be determined by the investigator according to RECIST v1.1
**Safety Objective**	**Corresponding Endpoints**
• To evaluate the safety of nivolumab plus ipilimumab treatment in subjects with poor-prognosis CUP (non-specific subset) who are resistant or refractory to platinum-based first-line chemotherapy	• Incidence, nature and severity of adverse events (AEs)<br>• Incidence and reasons for any interruptions, or premature discontinuation of any component of study treatment<br>• Clinically significant laboratory values and vital signs
**Exploratory Objectives**	**Corresponding Endpoints**
• To evaluate the mutagenic effects of platinum-based first-line chemotherapy in subjects with CUP	• Genomic profiles including TMB pre and post platinum-based first-line chemotherapy, as assessed by using the TruSight Oncology 500 (TSO500) panel from Illumina, cfDNA will be used for analysis in subjects for whom no post-chemotherapy tumor biopsy sample is available.
• To evaluate clonal evolution of CUP during cancer immunotherapy treatment	• Genomic profiles, TMB, MSI and PD-L1 expression pretreatment and at disease progression in subjects receiving cancer immunotherapy
• To determine the predictive impact of saliva and stool microbiome on response to nivolumab and ipilimumab.	• Microbiome analysis by sequencing of saliva and stool samples collected before study treatment start

CUP, cancer of unknown primary; ORR, objective response rate; OS, overall survival; PFS, progression-free survival; RECIST v1.1, Response Evaluation Criteria in Solid Tumors, version 1.1.

3 TRIAL DESIGN AND DESCRIPTION

3.1 Trial design

This study is a phase II, non-randomized, open-label, multi-center trial. The study will consist of a 42-day Screening Period, a Treatment Period, an End of Treatment Visit occurring 30 (± 7) days from last treatment or at initiation of other anti-cancer therapy (whichever occurs first), and a Follow-Up Period. The first day of treatment will be Day 1 (baseline) of the study.

The overall study design is presented in Figure 1. A Schedule of Activities is provided in the trial schedule (page 16 ff.). A timeline of key study events is summarized in the synopsis and further detailed below.

Figure 1 Study Design

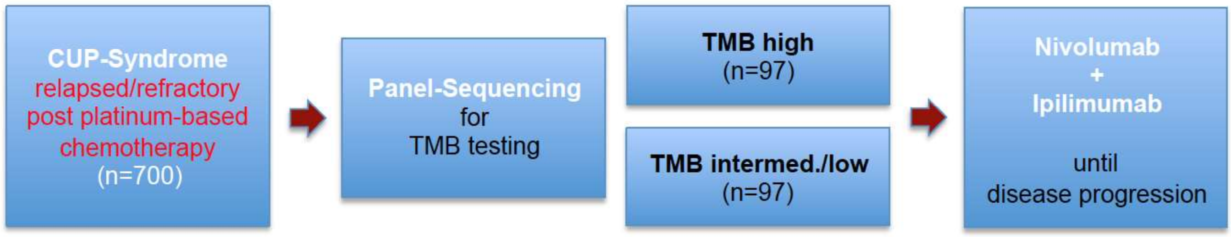

CUP, cancer of unknown primary site; TMB, tumor mutational burden; intermed., intermediate.

CUP diagnosis has to be confirmed according to the 2015 ESMO Clinical Practice Guidelines for CUP (Fizazi et al. 2015).

The tumor tissue sample has to be suitable for: 1) the initial diagnosis of CUP at the study site's local laboratory, and 2) central pathology (confirmation of the CUP diagnosis) and generation of a comprehensive genomic profile at the central reference pathology laboratory. If, after local diagnosis of CUP, insufficient tumor tissue remains for the central pathology laboratory to generate a TMB report, then a fresh biopsy sample must be collected during the Screening Period that meets the study's requirements. Likewise, when the tumor tissue available is older than 6 months, then a fresh biopsy sample must be collected during the Screening Period that meets the study's requirements. In case one attempt to perform TMB analysis on a new specimen has failed due to insufficient tumor cell quantity or insufficient quality in the specimen, or a re-biopsy has failed or cannot be performed for clinical or technical reasons, resorting to a specimen not older ≤24 months is allowed as an exception.

Blood samples which have to be suitable for analysis of circulating tumor DNA should be submitted prior to study treatment initiation and subsequently every three months, if subject consented to participate in sub-trial.

3.2 Screening Period

The Screening Period will be a maximum of 42 days long. Once all required screening tests have been performed and all inclusion criteria are met, but none of the exclusion criteria, the treatment phase can begin before the 42 days elapsed. By definition, CUP designates a metastatic malignancy where the primary tumor has remained elusive in spite of a thorough diagnostic work-up. The diagnosis of CUP and the diagnosis of the unfavorable CUP subset should follow the ESMO guidelines. Accordingly, the diagnosis of unfavorable CUP requires the exclusion of metastatic cancers with known primary as well as the exclusion of defined

favorable CUP subtypes. In tune with the ESMO guidelines the minimum requirements for CUP diagnosis in this study include a routinely performed up-to date CT or MRI scan of neck, chest and abdomen as well as histologic confirmation of malignancy. In case of a possible GI tract primary, gastroscopy and colonoscopy should be performed as well. Further diagnostic tests including a gynecologic exam should be performed as suggested by the clinical picture of the metastases and the immunohistological specimen. This diagnostic work-up, if thoroughly performed at first diagnosis before first line treatment, does not have to be repeated at study inclusion, at relapse or progression.

An immunohistological profile suggesting a likely primary that is not found by imaging is compatible with the diagnosis of CUP, whereas a clinical picture and immunohistological profile unequivocally pointing towards a primary tumor is not. In patients with large isolated hepatic metastases the differential diagnosis of cholangiocellular carcinoma should be considered and histology and imaging should be analyzed in this respect. TTF1-positive adenocarcinomas can be included only in the absence of thoracic (intrapulmonary, mediastinal) or thyroid masses.

To be eligible, subjects must have a histological diagnosis of CUP (non-specific subset), with available report, as determined by the study site's local laboratory on a tissue sample not older than 6 months. The non-specific subset of CUP should be diagnosed based on the clinico-pathologic criteria described in the 2015 ESMO Clinical Practice Guidelines for CUP (Fizazi et al. 2015). In brief, subjects must not have any of the non-CUP neoplasms identified in the ESMO guidelines (i.e., non-epithelial cancer, extragonadal germ-cell tumor, etc.), must not have an immunohistochemistry profile that provides a definitive clinical suspicion of a primary cancer with a specific treatment, and must not have any of the favorable-risk CUP subsets as identified in the ESMO guidelines. Acceptable disease will include:

- Adenocarcinoma of unknown primary site (ACUP)
- Poorly differentiated adenocarcinoma of unknown primary site
- Poorly differentiated carcinoma of unknown primary
- Squamous cell carcinoma of unknown primary site (SCUP)

Patients who can be assigned to a specific subset of CUP for which a specific treatment is recommended by guidelines will be excluded, including:

- Poorly differentiated carcinoma with midline distribution
- Women with papillary adenocarcinoma of the peritoneal cavity
- Women with adenocarcinoma involving only the axillary lymph nodes
- Squamous cell carcinoma of the cervical lymph nodes
- Poorly and well differentiated neuroendocrine tumors
- Men with blastic bone metastases and elevated PSA
- Patients with a single, small tumor potentially resectable and/or amenable to radiotherapy with curative intent
- Colon cancer-type CUP

Subjects must be relapsed or refractory after at least three cycles of platinum-based standard chemotherapy, must have ECOG performance status scores of 0 – 2, and must have at least one lesion that is measurable according to RECIST v1.1 (appendix 2). Tumor lesions situated in a previously irradiated area, or in an area subjected to other loco-regional therapy, are usually not considered measurable unless there has been demonstrated progression in the lesion.

As this study aims to stratify treatment according to TMB, it is mandated that subjects are willing and able to provide:

- A tumor tissue sample suitable for confirmation of the CUP diagnosis and for generation of a TruSight Oncology 500 (TSO500) genomic profile at the central reference pathology laboratory.
- Archival tumor FFPE block ≤ 6 months prior to screening will be acceptable for these central analyses. However, if an acceptable archival tumor FFPE block is not available at screening, an FFPE block from a freshly obtained biopsy sample must be provided that meets the study's requirements.

TMB will be analyzed from FFPE tumor tissue samples no older than ≤ 6 months prior to screening after the patients consented to take part in the CheCUP study. If the TMB value was already previously determined using the TSO500 panel during clinical routine from tissue that is no older than ≤ 6 months prior to screening, the TMB value can be used and the TMB analysis does not need to be repeated. If no archival material no older than ≤ 6 months prior to screening is available, a fresh biopsy has to be taken. In case one attempt to perform TMB analysis on a new specimen has failed due to insufficient tumor cell quantity or insufficient quality in the specimen, or re-biopsy has failed or cannot be performed for clinical or technical reasons, resorting to a specimen not older ≤24 months is allowed as an exception.

Subjects will be stratified according to their TMB value to the TMB low/intermediate and TMB high group, respectively. The study design aims at balanced strata sizes with 50% of subjects (n = 97) belonging to the TMB-high and 50% (n = 97) to the TMB-intermediate/low group, respectively. Balancing of strata to a TMB-high : TMB-intermediate/low ratio of 50 : 50 will be done at the total study level but not at the single center level. Alternating recruitment of patients into TMB-high and TMB-intermediate/low strata is intended. An excess of up to 5 patients in one of the strata will be allowed. Notification of study centers on the qualification of individual patients for study participation on the basis of their respective TMB levels will be done by the coordinating investigator's/LKP's office. Patients who cannot enter the study for strata balancing reasons can remain on a waiting list for a maximum of 4 weeks. An intermediate chemotherapy while on the waiting list might be performed. Only patients qualified for the clinical trial based on the notification from the coordinating investigator are allowed to receive study treatment.

For the generation of the patients' genomic profiles genomic DNA is isolated from the obtained FFPE blocks. Subsequently, the genomic DNA is fragmented and a DNA library is prepared by Index PCR. The DNA library is hybridized with a specific oligo pool (TSO500), consisting of ~ 500 genes, to enrich DNA covering 1.34 Mb or 4 % of the coding human exome. The enriched DNA is panel sequenced by next generation sequencing using a NextSeq500 (Illumina). The results are assessed and analyzed by the manufacturer's provided software TruSight™ Oncology local app (version 1.3). All detected gene variants are manually validated before the TMB value is calculated. Results from these analyses will be used to stratify subjects for high

versus intermediate/low TMB. (TMB-high ≥ 12 mutations/Mb, TMB-intermediate/low < 12 mutations/Mb). For further analyses, the TMB-intermediate/low group will be subdivided into TMB-intermediate and TMB-low groups (≥6 to <12 and <6 mutations/Mb), respectively. In addition, as TMB is a continuous variable (Buchhalter et al. 2018) that is measured quantitatively, we will also explore percentiles of TMB (highest 10%, 20%, 30% of cases compared to the overall cohort) and their associations with response (Samstein et al. 2019).

3.3 Treatment Period

All subjects fulfilling eligibility and stratification criteria will be enrolled into the trial. They enter the Treatment Period, where they will receive nivolumab as a 30-minute infusion, 240 mg flat dose every 2 weeks and ipilimumab as a 30-minute infusion, 1 mg/kg every 6 weeks, starting on Day 1, until progression, unacceptable toxicity, investigator or subject decision to withdraw from therapy or death, whichever occurs first.

When nivolumab and ipilimumab are to be administered on the same day, separate infusion bags and filters must be used for each infusion. Nivolumab is to be administered first. The second infusion will always be ipilimumab and will start no sooner than 30 minutes after completion of the nivolumab infusion. Subjects who require small volumes may infuse < 30 minutes but no less than 20 minutes. Nivolumab and ipilimumab may be diluted in 0.9% Sodium Chloride Solution or 5% Dextrose solution. Dosing calculations should be based on the body weight. If the subject's weight on the day of dosing differs by > 5% from the weight used to calculate the prior dose, the dose must be recalculated. Use baseline weight at cycle 1 and prior dose weight at cycle 2 and onwards to calculate weight difference. All doses should be rounded to the nearest milligram. There will be no dose modifications allowed. Subjects may be dosed with nivolumab no less than 12 days from the previous dose. There are no premedications recommended. Subjects should be carefully monitored for infusion reactions. If an acute infusion reaction (anaphylactic reaction and shock, bronchospasm, hypersensitivity, and infusion-related reaction) is noted, infusion of nivolumab plus ipilimumab or nivolumab should be immediately interrupted and respective anti-allergic treatment should be initiated promptly. In such a situation use of corticosteroids is possible without restrictions. Doses of nivolumab and/or ipilimumab may be interrupted, delayed, or discontinued depending on how well the subject tolerates the treatment. For more details, see sections 5.4.7 (dose delays) and 5.4.8 (discontinuations). For the purpose of homogeneity, an assessment cycle will be considered to be 42 days.

3.4 Post-Treatment Period

Following discontinuation of study treatment, subjects will return to the clinic 30 (± 7) days from the last treatment or at initiation of another anti-cancer therapy (whichever occurs first) (End of Treatment Visit). Patients will be contacted by their physician by phone at day 100 (± 7) from the last treatment for a safety follow-up. AEs are to be recorded until day +100 after the final dose of study treatment. Thereafter, subjects will be contacted by the physician by phone every 3 (± 1) months for survival follow-up.

Subjects who discontinue for reasons other than PD but who do not withdraw consent to follow up will be assessed for progression as described during the Treatment Period (including scans)

until disease progression. After disease progression, these subjects will continue with survival follow up.

3.5 Translation Research

Within a translational project cfDNA serial plasma samples ("liquid biopsies") will be collected at screening, staging visits and EoT visit from those subjects who consent to do so. These samples will be analysed (i) by panel sequencing in order to detect newly developed mutations as sign of tumor evolution and (ii) by digital PCR to quantify mutations in order to monitor treatment response and progression.

In a further translational project one saliva and one stool sample will be collected at screening from those subjects who consent to do so. The microbiome of these samples will be analysed by sequencing in order to determine bacterial flora associated with treatment response as potential predictive marker.

3.6 Trial Duration and Schedule

The end of study will occur when all enrolled subjects have either died, withdrawn consent, are lost to follow up, or have been followed for 12 months after the last study patient is enrolled, whichever occurs first.

Recruitment is expected to occur over approximately 24 months. It is therefore estimated that the study will last for a total of approximately 36 months.

Recruitment of subjects started in December 2019. The actual overall or recruitment duration may vary. A planned interim futility analysis will be performed after recruitment of 97 subjects. The study end is defined as “last Subject Out” (LSO).

Total trial duration:	36 months
Duration of the clinical phase:	24 months
Beginning of the preparation phase:	October 2018
FSI (First Subject In):	12/2019
LSI (Last Subject In):	12/2021
LSO (Last Subject Out):	12/2022
DBL (Data Base Lock):	[Q1 2023]
Statistical analyses completed:	[Q2 2023]
Trial report completed:	[Q2 2023]

4 SELECTION OF SUBJECTS AND CENTRES

4.1 Number of Subjects

As calculated in section 9.1, 194 subjects will be enrolled in the clinical trial, i.e. 97 subjects with high and intermediate/low TMB, respectively. Recruitment and treatment of subjects will be performed in approximately 10 trial sites.

As described above, TMB is expected to be high versus intermediate/low in about 15% and 85% of CUP subjects (Gay et al. 2017; Krämer et al. 2018). As this asymmetric patient distribution between the two groups would lead to an increased sample size of subjects needed to be recruited into the trial in order to demonstrate a statistical significant difference in response to nivolumab plus ipilimumab treatment (section 9.1), a subject enrichment strategy will be used to increase the ratio of subjects with TMB-high versus TMB-intermediate/low to 50 : 50. For that, TMB will be analyzed by TSO500 panel for all subjects from the diagnostic FFPE tumor tissue sample which will be available from initial diagnosis for each subject. If not enough biopsy material is available for genomic profiling, an additional biopsy has to be taken in order to allow for TMB determination before study entry.

An interim futility analysis is scheduled after inclusion of 97 subjects in the full analysis set. If the study continues without adaptation, the final analysis will be performed after inclusion of 97 further subjects.

4.2 Trial Sites

The study will be conducted on a national, multicentre basis. It is intended that the study will take place at about 10 German sites. All sites are large university hospitals, community hospitals or oncological practices with dedicated, high-volume oncology units and large numbers of CUP subjects. In addition, each of the sites has a clinical trial centre and study nurses specifically dedicated to execution and documentation of the CheCUP study.

4.3 General Criteria for Subjects' Selection

As there will be no preferences on the selection of gender to be included, it is anticipated that the study results will give a representative gender distribution, which should reflect the natural gender distribution in the underlying disease.

4.4 Inclusion Criteria

Subjects meeting all of the following criteria will be considered for admission to the trial:

- Signed Informed Consent Form
- Able and willing to comply with the study protocol
- Age ≥ 18 years at time of signing Informed Consent Form
- Histologically-confirmed disseminated or advanced unresectable CUP diagnosed according the criteria defined in the 2015 ESMO Clinical Practice Guidelines for CUP (Fizazi et al. 2015). Acceptable disease histology includes:
    - Adenocarcinoma of unknown primary site (ACUP)
    - Poorly differentiated adenocarcinoma of unknown primary site
    - Poorly differentiated carcinoma of unknown primary site
    - Squamous cell carcinoma of unknown primary site (SCUP)

Advanced unresectable disease should not be amenable to resection and/or irradiation with curative intent during the course of the study

- At least one lesion that is measurable according to RECIST v1.1 (appendix 2) by CT/MRI
    - If a fresh biopsy is needed during Screening, the biopsy procedure must not affect measurability of disease
    - Tumor lesions situated in a previously irradiated area, or in an area subjected to other loco-regional therapy, are usually not considered measurable unless there has been demonstrated progression in the lesion.
- Availability of a tumor FFPE block either fresh or archival if obtained ≤ 6 months at Screening that is sufficient for generation of a TruSight Oncology 500 (TSO500) panel at the central reference pathology laboratory (MPZ, Institute of Pathology, University of Heidelberg; refer to the Laboratory Manual for specimen collection instructions) or pre-existing result of a TMB analysis from routinely performed panel sequencing using the TSO500 panel at the MPZ, which must not be older than ≤ 6 months at screening, respectively. In case one attempt to perform TMB analysis on a new specimen has failed due to insufficient tumor cell quantity or insufficient quality in the specimen, or re-biopsy has failed or cannot be performed for clinical or technical reasons, resorting to a specimen not older ≤24 months is allowed as an exception.
- Availability of test reports confirming local CUP diagnosis. If test reports confirming a local CUP diagnosis are not available, an FFPE block or a fresh biopsy sample must be submitted that is sufficient to allow for central confirmation of CUP diagnosis.
- Disease relapse or progression after at least three cycles of a platinum-based standard chemotherapy not limited to those listed in Table 2. There is no upper limit of prior treatments received.
- Subjects who have received prior surgery and/or radiotherapy and/or stereotactic brain metastasis radiosurgery are eligible. In case of prior radiotherapy, the measurable lesion(s) must not have been irradiated, radiotherapy has to be finished at least 7 days before start of study treatment and the patient must have recovered to grade 1 or less from any toxicity of radiotherapy.
- ECOG performance status of 0 - 2
- Life expectancy ≥ 12 weeks
- Eligible for immune checkpoint inhibitor
- Adequate hematologic and end-organ function, defined by the following laboratory results obtained within 14 days prior to initiation of study treatment:
    - Absolute neutrophil count (ANC) ≥ 1.0 × 10e9 cells/L, corresponding to ≥ 1000/µl (without granulocyte colony-stimulating factor support within 2 weeks prior to the first study treatment)
    - Platelet count ≥ 80 × 10e9 cells/L, corresponding to 80,000/µl (without transfusion within 2 weeks prior to the first study treatment)
    - Hemoglobin ≥ 90 g/L (9.0 g/dL)
      Subjects may be transfused or receive erythropoietic treatment to meet this criterion

- Aspartate transaminase (AST) and alanine transaminase (ALT) ≤ 3 times the upper limit of normal (ULN)

- Serum bilirubin ≤ 1.5 × ULN
  Subjects with known Gilbert disease who have serum bilirubin level ≤ 3 × ULN may be enrolled

- Creatinine clearance ≥ 30 mL/min

- For subjects not receiving therapeutic anticoagulation: International normalized ratio (INR) or activated partial thromboplastin time (aPTT) ≤ 1.5 × ULN
  Subjects receiving heparin treatment should have an aPTT between 1.5 to 2.5 × ULN (or patient value before starting heparin treatment). Subjects receiving coumarin derivatives should have an INR between 2.0 and 3.0 assessed in two consecutive measurements 1 to 4 days apart. Subjects receiving a therapy with other anticoagulants do not need additional testing.

- For women of childbearing potential and men capable of reproduction: agreement to remain abstinent (refrain from heterosexual intercourse) or use contraceptive methods with a failure rate of < 1% per year during the treatment period and for at least 5 months for women and 7 months for men, respectively after the last dose of study treatment. A woman is considered to be of childbearing potential if she is postmenarcheal, has not reached a postmenopausal state (≥ 12 continuous months of amenorrhea with no identified cause other than menopause), and has not undergone surgical sterilization (removal of ovaries and/or uterus)
  - Examples of contraceptive methods with a failure rate of < 1% per year include bilateral tubal ligation, male sterilization (with appropriate post-vasectomy documentation of the absence of sperm in the ejaculate), hormonal contraceptives that inhibit ovulation, hormone-releasing intrauterine devices, and copper intrauterine devices. Hormonal contraceptive methods <u>must</u> be supplemented by a barrier method.
  - The reliability of sexual abstinence should be evaluated in relation to the duration of the clinical trial and the preferred and usual lifestyle of the patient. Periodic abstinence (e.g., calendar, ovulation, symptothermal, or postovulation methods) and withdrawal are not acceptable methods of contraception.
  - Women of childbearing potential must have a negative highly sensitive serum pregnancy test result within 3 days prior to first dose.

- Recovery from significant toxicity from platinum-doublet therapy to Grade ≤ 1, except for alopecia, fatigue and for neurosensory toxicity, which must be ≤ 2

- Recovery from active infections requiring intravenous antibiotics, with antibiotic therapy ceased for ≥ 7 days prior to planned start of therapy

4.5 Exclusion Criteria

Subjects who meet any of the following criteria will be excluded from study entry:

- Subjects with any of the specific non-CUP neoplasms identified in the ESMO CUP guidelines (Fizazi et al. 2015)
- Subjects belonging to any of the following subsets of CUP with favorable prognoses:
    - Poorly differentiated carcinoma with midline distribution
    - Women with papillary adenocarcinoma of the peritoneal cavity
    - Women with adenocarcinoma involving only the axillary lymph nodes
    - Squamous cell carcinoma restricted to cervical lymph nodes
    - Poorly and well differentiated neuroendocrine tumors
    - Men with blastic bone metastases and elevated PSA
    - Subjects with a single, small tumor potentially resectable and/or amenable to radiotherapy with curative intent
    - Colon cancer-type CUP
- Known presence of brain or spinal cord metastasis, as determined by CT or magnetic resonance imaging (MRI) evaluation during screening. As an exception, patients with brain metastases are allowed to be included if <u>all</u> of the following five criteria are met:
    (i) the total number of brain metastases is 3 or less,
    (ii) brain metastases were / are asymptomatic,
    (iii) brain metastases have been completely surgically resected or completely treated with stereotactic radiosurgery
    (iv) there was / is no indication for whole-brain irradiation,
    (v) a brain MRI or high-resolution CT-scan at screening shows no evidence of residual disease.

    If 1 to 3 asymptomatic brain metastases are detected at screening and are treatable and treated with stereotactic radiosurgery within the screening period, no renewed MRI / CT imaging of the brain is required before inclusion.

    Benign lesions such as meningiomas may be accepted, if demonstration is made that they will not affect the interpretation of the study results or render the patient at high risk from treatment complications.
- History or known presence of leptomeningeal disease
- Uncontrolled or symptomatic hypercalcemia (serum calcium $\geq$ 2.9mmol/L)
- Known clinically significant history of liver disease consistent with Child-Pugh Class B or C, including active viral or other hepatitis, current alcohol abuse, or cirrhosis
- Human immunodeficiency virus (HIV) infection
- Positive for hepatitis C virus (HCV) infection at screening
- Positive for hepatitis B surface antigen (HBsAg) at screening
- Active tuberculosis at screening

- Significant cardiovascular disease (such as New York Heart Association Class II or greater cardiac disease, myocardial infarction, or cerebrovascular accident) within 3 months prior to initiation of study treatment, unstable arrhythmia (including active ventricular arrhythmia requiring medication), or unstable angina
- Major surgical procedure, other than for diagnosis, within 4 weeks prior to initiation of study treatment, or anticipation of need for a major surgical procedure during the study
- History of malignancy other than CUP within 5 years prior to screening, with the exception of malignancies with a negligible risk of metastasis or death (e.g., 5-year OS rate $>$90%), such as adequately treated carcinoma in situ of the cervix, non-melanoma skin carcinoma, localized prostate cancer, ductal carcinoma in situ, or Stage I uterine cancer
- Solid organ transplantation
- Prior allogeneic stem cell transplantation with follow-up < 1 year, need for systemic immunosuppression or active chronic graft-versus host disease (cGVHD)
- Any other disease, metabolic dysfunction, physical examination finding, or clinical laboratory finding that contraindicates the use of an investigational drug, may affect the interpretation of the results, or may render the patient at high risk from treatment complications
- Known allergy or hypersensitivity to any component of the immunotherapy, including history of severe allergic anaphylactic reactions to chimeric or humanized antibodies or fusion proteins and to Chinese hamster ovary cell products or other recombinant human or humanized antibodies for nivolumab and ipilimumab.
- Subjects with an active autoimmune disease, including, but not limited to, myasthenia gravis, myositis, autoimmune hepatitis, myocarditis, systemic lupus erythematosus, rheumatoid arthritis, inflammatory bowel disease, antiphospholipid antibody syndrome, Wegener granulomatosis, Sjögren syndrome, Guillain-Barré syndrome, or multiple sclerosis. Subjects with type I diabetes mellitus, hypothyroidism only requiring hormone replacement, skin disorders (such as vitiligo, psoriasis, or alopecia) not requiring systemic treatment, or conditions not expected to recur in the absence of an external trigger are permitted to enroll.
- Subjects with a condition requiring systemic treatment with either corticosteroids (> 10 mg daily prednisone equivalents), or other immunosuppressive medications within 14 days of study treatment. Inhaled or topical steroids and adrenal replacement doses > 10 mg daily prednisone equivalents in the absence of active autoimmune disease are permitted.
- Subjects who received prior treatment with an anti-PD-1, anti-PD-L1, anti-PD-L2, anti-CD137, or anti-CTLA-4 antibody, or any other antibody or drug specifically targeting T-cell co-stimulation or checkpoint pathways
- All toxicities attributed to prior anti-cancer therapy other than alopecia, neurosensory toxicity and fatigue must have resolved to Grade 1 (NCI CTCAE version 5) or baseline before administration of study drug. Subjects with toxicities attributed to prior anti-cancer therapy which are not expected to resolve and result in long lasting sequelae, such as neuropathy after platinum-based therapy, are permitted to enroll.
- Systemic treatment for cancer (any chemotherapy, biologics for cancer or investigational therapy within 21days of first administration of study treatment.

- Radiotherapy or stereotactic brain metastasis radiosurgery has to be finished at least 7 days before inclusion into the study and the subject must have recovered to grade 1 or less from any toxicity of radiotherapy / stereotactic brain metastasis radiosurgery.
- Subjects must not have received a live / attenuated vaccine within 30 days of first treatment.
- Pregnancy or breastfeeding, or intention of becoming pregnant during study treatment or within 5 months after the last dose of study treatment or intention of fathering a child within 7 months after the last dose of study treatment.

No subject will be allowed to start study treatment more than once. However, a patient can be screened more than once, e.g. if a TMB report could not be generated in time. For each screening a new screening number will be assigned.

4.6 Criteria for Withdrawal

4.6.1 *Withdrawal of Subjects from Treatment*

Any subject can withdraw from the treatment at any time without personal disadvantages and without having to give a reason. Subjects who discontinue participation in the clinical study on their own or subjects who are withdrawn by the investigator, for reasons other than disease progression (for example in case of AEs or protocol violations, see also table 4), will be defined as premature withdrawals. Premature withdrawals will not be replaced. The time of treatment discontinuation must be documented in the patient file and on the CRF and sponsor and if applicable LKP and DMC must be informed written form.
The investigator can also discontinue the study after considering the risk-to-benefit ratio, if he/she no longer considers the further treatment of the subject according to study protocol justifiable. The date of and the primary reason for the withdrawal, as well as the observations available at the time of withdrawal are to be documented on the CRF. Reasons leading to the withdrawal of a patient can include the following (**one primary reason must be determined**):

- **Lack of efficacy** of the study medication, e.g.
  - Progress of CUP compared to baseline (unless the patient may benefit clinically of treatment beyond progression and consent from sponsor has been obtained)
  - Need for a prohibited concomitant medication for the treatment of CUP
  - Use of another anti-cancer therapy not permitted in the protocol
- **Intolerable adverse events** (like severe autoimmune disorders or neurologic side effects)
- **Lack of patient's cooperation**, e.g.
  - Patient's request to withdraw
  - Lack of compliance, patient fails to attend the interim visits as agreed
  - Existing or intended pregnancy, lactation
- **Pregnancy**
- **Any medical condition the investigator or the sponsors deems as a risk to the patient and his safety when continuing with study treatment**
- **Decision on the part of the Investigator or Sponsor that withdrawal from the study is in the patient´s best interest**
- **Other reasons** (noting reason), e.g.
  - Did not meet major in-/exclusion criteria (coming to light after study inclusion)

If violation of inclusion / exclusion criteria becomes evident after enrollment and safety of the participant is affected, the patient has to be withdrawn from the study to ensure patient´s safety.

If exclusion criteria not relating to the diagnosis of CUP and the discrimination of favorable CUP types become evident after enrollment and safety of the participant is not affected, the patient has nevertheless to be withdrawn from the study to ensure the integrity of the trial.

If exclusion criteria relating to the diagnosis of CUP and the discrimination of favorable CUP types become evident after enrollment (e.g. the demasking / detection of the primary cancer while the patient is on study or new medical findings pointing towards favorable subset CUP) and safety of the participant is not affected, the patient can remain in the study after consultation with the principle investigator / coordinating investigator (Leiter der klinischen Prüfung) provided that continued treatment within the trial is considered to be in the interest of the patient.

In all subjects who finish the study prematurely, a withdrawal examination at least with respect to the primary endpoint should be carried out. The subject must be asked to consent to this last examination. Hereby, oral consent is deemed sufficient. The withdrawal examination must be documented in the CRF.

If a subject does not come to a visit, the reason should be clarified. If the subject wants to withdraw, the reason should be documented in the subjects file and in the CRF. If the subject withdraws, the reason should be asked for in detail and documented in detail, if the subject is willing to explain himself.

For documentation of AE and SAEs see 8.2 and 8.1.7.

Table 4 Definitions and types of premature study withdrawal

**Term**	**Definition**
Drop-out, study	Participation terminated completely, including follow-up<br>Possible reasons:<br>• Patient withdraws consent: **Withdrawal**<br>• Patient moved/cannot be contacted<br>• Follow-up-interventions cannot be performed due to medical reasons<br>• Non-compliance of patient<br>Drop-out after completion of study intervention: **Lost to follow-up**<br>FU-CRF-forms need to be marked as invalid.<br>Final examination will be performed, if patient agrees. Study-Completion/ Withdrawal-form will be completed.
Drop-out, study intervention	Termination of study intervention, follow-up as per protocol.
Screening-failure	Exclusion criteria given prior to screening / enrolment:<br>Patient will be recorded at screening list, but will not be provided with a patient number.<br>(depending on sponsor a screening CRF may have to be completed)
Protocol deviation	Drop-out to study and drop-out to study intervention are both protocol deviations.

	It needs to be predefined, how to manage each type of protocol deviation.<br><br><u>major deviations:</u><br>If exclusion criteria become evident after enrollment, and safety of the participant is affected, or if the diagnosis does not any more relate to the indication listed in the protocol affecting the benefit/risk negatively, the participant has to be excluded from study intervention. FU-examinations may still be performed.<br><br><u>Minor deviations:</u><br>Other protocol deviations (errors in timing of visits/ missing samples/ missing examinations) do not result in exclusion

4.6.2 *Premature Closure of the Clinical Trial or a Trial Site*

If new information on the risk-to-benefit ratio of the drug or on the treatment methods used in the study is obtained in the meantime and safety concerns arise, the sponsor reserves the right to interrupt or terminate the project. Premature termination is also possible if the sponsor notices and agrees upon that patient recruitment is insufficient and that this cannot be expedited by appropriate measures.

Premature termination of a single trial site is also possible if the sponsor notices that the conduction of the trial is not compliant with ICH-GCP and / or is not according to the protocol, the patient recruitment and / or the quality of the data is insufficient.

The DMC can recommend interruption or termination of the study or of treatment arms based on the results of the intermittent SAE evaluation or of accumulating information on the above-mentioned reasons.

The ethics committee (EC) and the competent authorities must be informed about the premature closure of the trial or one of the treatment arms. Furthermore, the ethics committee(s) and competent authorities themselves may decide to stop or suspend the trial.

All involved investigators have to be informed immediately about a cessation / suspension of the trial. The decision is binding to all trial centres and investigators.

When the trial is closed, all study documentation must be stored at the trial site. Study medication must be sent to BMS or designee.

5 INVESTIGATIONAL MEDICINAL PRODUCTS (IMP)

5.1 Study medication

5.1.1 *General information about Nivolumab*

Nivolumab (also referred to as BMS-936558, MDX1106, or ONO-4538) is a human monoclonal antibody (HuMAb; immunoglobulin G4 [IgG4]-S228P) that targets the programmed death-1(PD-1) cluster of differentiation 279 (CD279) cell surface membrane receptor. PD-1 is a negative regulatory molecule expressed by activated T and B lymphocytes. Binding of PD-1 to its ligands, programmed death–ligands 1 (PD-L1) and 2 (PD-L2), results in the down-regulation of lymphocyte activation. Inhibition of the interaction between PD-1 and its ligands promotes immune responses and antigen-specific T-cell responses to both foreign antigens as well as self-antigens. Nivolumab is expressed in Chinese hamster ovary (CHO) cells and is produced using standard mammalian cell cultivation and chromatographic purification technologies. The clinical study product is a concentrated sterile solution for intravenous administration.

The physical and chemical properties of Nivolumab drug substance are described in the investigator's Brochure (IB).

5.1.2 *General information about Ipilimumab*

Ipilimumab (BMS-734016, MDX010, MDX-CTLA4) is a fully human monoclonal immunoglobulin (Ig) G1κ specific for human cytotoxic T-lymphocyte antigen 4 (CTLA-4), which is expressed on a subset of activated T cells. CTLA-4 is a negative regulator of T-cell activity. Ipilimumab binds to CTLA-4 and blocks the interaction of CTLA-4 with its ligands CD80 and CD86. Blockade of CTLA-4 has been shown to augment T-cell activation and proliferation, including the activation and proliferation of tumor-infiltrating T-effector cells. Inhibition of CTLA-4 signaling can also reduce T-regulatory cell function, which may contribute to a general increase in T-cell responsiveness, including the anti-tumor response.

Ipilimumab is expressed in Chinese hamster ovary (CHO) cells and is produced by DNA recombinant technologies. The clinical study product is a concentrated sterile solution for intravenous administration.

The physical and chemical properties of Ipilimumab drug substance are described in the investigator's Brochure (IB).

5.2 Supply, Packaging and Labelling of IMPs

Nivolumab and Ipilimumab will be labelled according to § 5 of GCP-V by BMS and packed in cartons of either 5 vials (Nivolumab) or 4 vials (Ipilimumab), respectively. BMS will provide to the local pharmacies the quantity of Nivolumab and Ipilimumab required for the clinical trial depending on the recruitment rate. The IMP provided must be used only in the context of this clinical trial.

5.3 Supplies and Drug Accountability

The investigator will confirm correct receipt of the trial medication in writing and ensure that the medication is stored safely and correctly. The trial medication must be carefully stored in accordance with the current IB at 2-8°C and protected from light at the trial sites in a locked area with restricted access, separately from other drugs, and kept out of the reach and sight of children. The investigator will document the distribution and return of the IMP to the subject with the date, recording the quantity distributed and used on the forms provided for this purpose. The site monitor will periodically check the supplies of IMP held by the investigator or local pharmacy, respectively to ensure the correct accountability of all IMP used. At the end of the trial, all unused IMP and all medication containers will be completely returned to BMS or

designee. It will be assured that a final report of the drug accountability is prepared and maintained by the investigator.

5.4 Administration of study medication

5.4.1 *Assignment of Identification Codes*

All subjects who seem suitable for study participation and take part in the screening will receive a screening number. The screening number consists of the two-digit trial site number followed by a three-digit consecutive number starting with 001 at each trial site. At the end of the 42-day screening phase or after completion of all screening procedures, respectively, the eligibility of the subject is assessed finally. The coordinating investigator Prof. Krämer will decide if a subject can be enrolled or has to enter a waiting list based on the result of the TMB value (high/low - intermediate). The study treatment cannot be started without the approval of the coordinating investigator.

When the subject is included in the study (all inclusion and stratification criteria and none of the exclusion criteria apply), the subject will keep his screening number as enrollment code/subject number. Subjects withdrawn from the study retain their subject number. If subjects change the assigned trial site during the treatment period, they keep their original subject number. Reasons for changing a trial site could be for example a move, or a nearby trial site is activated for the CheCUP study.

The reconstitution of the IMP as an intravenous dose specifically calculated per subject will be performed by trained pharmacists in the local pharmacy. Each reconstitution of IMP will be witnessed and all steps will be documented by a second trained pharmacist.

The intravenous administration of the IMPs will be supervised by trained study personnel according to the below described dosage schedule. Subjects should be carefully monitored for infusion reactions during the time of the infusions and 30 minutes afterwards (see also section 8.6, safety management).

5.4.2 *Dosage Schedule*

The IMPs are as outlined in the table 5 and section 3.4 Treatment Period.

Table 5 Doses and Infusion times for intravenous administration of nivolumab and ipilimumab

Study Drug	Drug Dose	Treatment Frequency
**Nivolumab**	240 mg flat dose over 30 minutes	Every 2 weeks
**Ipilimumab**	1 mg/kg over 30 minutes	Every 6 weeks

Nivolumab should be first administered followed by ipilimumab when administered at the same day. For each drug should be a separate infusion set and an in-line, sterile, non-pyrogenic, low protein binding filter (pore size of 0.2 µm to 1.2 µm) be used.

The study treatment will be continued until disease progression, intolerance, investigator’s or subject’s decision to withdraw from therapy.

5.4.3 *Compliance*

Because all study drugs will be given i.v. in the trial sites, noncompliance is not considered an issue.

5.4.4 *Prior and Concomitant Diseases*

Relevant additional diseases present at the time of informed consent are regarded as concomitant diseases and will be documented on the appropriate pages of the case report form (CRF). Included are conditions that are seasonal, cyclic, or intermittent (e.g. seasonal allergies; intermittent headache).

Abnormalities which appear for the first time or worsen (intensity, frequency) during the trial are adverse events (AEs) and must be documented on the appropriate pages of the CRF.

5.4.5 *Prior and Concomitant Medication*

Prohibited medications

- Any concomitant chemotherapy or anti-cancer agent to treat CUP other than the study medication within 21 days of study treatment
- Systemic immunosuppressants including cyclosporine, tacrolimus, sirolimus, mycophenolate, leflunomide, cyclophosphamide, methotrexate or TNFα blockers within 14 days of study treatment, unless indicated for treatment of irAEs

Medications permitted under restrictions

Treatment with systemic steroids within 14 days of study treatment (prednisone, prednisolone and dexamethasone) is prohibited, with the following exceptions

- Participants with a condition requiring systemic treatment with corticosteroids at a dose of ≤ 10 mg daily prednisone equivalents in the absence of active autoimmune disease
- Participants receiving adrenal replacement doses > 10 mg daily prednisone equivalents are permitted in the absence of active autoimmune disease
- Participants receiving Inhaled or topical steroids
- Steroids are permitted in the course of the trial to treat autoimmune side effects induced by study treatment without limitation. Patients are also allowed to continue with study treatment while still on steroid treatment.

Mandatory medications

- None

Permitted medications

- Any medication not listed among prohibited, for corticosteroids see restrictions above

If concomitant drugs are administered, these must be recorded in the subject file and in the CRF, stating

- The type (preferably the generic name / INN, or trade name)
- The route of administration
- The regimen including dosage schedule, daily dose (if not indicated by the type), and form of application
- The indication
- The duration

5.4.6 *Adjustments to dosage and delays in administration of nivolumab and ipilimumab in the individual trial subject*

No dose reductions of either nivolumab and ipilimumab are allowed, however, administration of both drugs could be delayed for adverse events (see 5.4.7).

5.4.7 *Dosing delay and discontinuation criteria of study treatment*

It is mandatory to withhold study treatment with both nivolumab and ipilimumab if one of the following AEs occurs on the day of planned treatment:

- Either febrile neutropenia or neutropenia <500 cells/mm³ (use of growth factors allowed)
- Any AE, laboratory abnormality or intercurrent illness which, in the judgment of the investigator, warrants delaying the dose of study medication
- Guidelines for withholding or discontinuing treatment for immune-mediated side effects should closely follow the investigator brochure (IB). The treatment and dosing recommendations are listed in table 6 below. Very detailed algorithms for treatment of side effects are provided in Appendix Management Algorithms of the Investigator brochures.

Table 6 Discontinuation / Delay Criteria

	CTCAE Grade	Treatment	Continuation / Discontinuation of Study Treatment
**Immune mediated pneumonitis**	Grade 3-4	corticosteroids at 1-2 mg/kg/day prednisone equiv. dose, then taper	discontinue permanently
	Grade 2	corticosteroids at 1-2 mg/kg/day prednisone equiv. dose, then taper	withhold dose, if not improving after two weeks or worsening, study treatment is discontinued.
**Immune mediated hepatitis**	Grade 3-4<br>AST/ALT > 5 ULN* or Bili >3 ULN*	corticosteroids at 1-2 mg/kg/day prednisone equiv. dose, then taper	discontinue permanently
	Grade 2<br>AST/ALT >3 - ≤5 ULN or Bili >1.5 - ≤3 ULN *	corticosteroids at 0.5-1 mg/kg/day prednisone equiv. dose, then taper	withhold dose, resume treatment when adverse reaction improves to Grade 0 or 1.
**Immune mediated colitis / diarrhea**	Grade 3-4	corticosteroids at 1-2 mg/kg/day prednisone equiv. dose, then taper	discontinue permanently
	Grade 2	corticosteroids at 0.5-1 mg/kg/day prednisone equiv. dose for colitis of > 5 days duration,<br>if no improvement, increase to 1-2 mg/kg/day, then taper	withhold dose, resume treatment when adverse reaction improves to Grade 0 or 1.<br>If worsening or persisting > 3-5 days with oral steroids study treatment is discontinued

**Hypophysitis**	Grade 4	corticosteroids at 1 mg/kg/day, then taper	discontinue permanently
	Grade 2-3	corticosteroids at 1 mg/kg/day, then taper	withhold dose
**Adrenal insufficiency**	Grade 3-4	corticosteroids at 1-2 mg/kg/day prednisone equiv. dose, then taper	discontinue permanently
	Grade 2		withhold dose, resume treatment when adverse reaction improves to Grade 0 or 1.
**hypothyreoidism**	All Grades	L-Thyroxin substitution	no dose adjustments
**type 1 diabetes**	Grade 4 hyperglycemia	Diabetes treatment	discontinue permanently
	Grade 3 hyperglycemia	Diabetes treatment	withhold dose
**nephritis / renal dysfunction**	Grade 4 (serum creatinine > 6 ULN)	corticosteroids at 1-2 mg/kg/day prednisone equiv. dose, then taper	discontinue permanently
	Grade 2-3 (serum creatinine >1.5 - ≤6 ULN)	corticosteroids at 0.5-1 mg/kg/day prednisone equiv. dose,<br>then taper	withhold dose, resume treatment when adverse reaction improves to Grade 0 or 1.<br>If persisting for longer than 7 days or worsening, study treatment is discontinued.

**skin toxicity**	Grade 4 or confirmed Stevens-Johnson syndrome / toxic epidermal necrolysis<br>Grade 3 pruritus	corticosteroids at 1-2 mg/kg/day prednisone equiv. dose, then taper	discontinue permanently
	Grade 3 or suspected Stevens-Johnson syndrome / toxic epidermal necrolysis	corticosteroids at 1-2 mg/kg/day prednisone equiv. dose, then taper	withhold dose, resume treatment when adverse reaction improves to Grade 0 or 1.
**encephalitis**	Immune mediated encephalitis	if infection ruled out corticosteroids at 1-2 mg/kg/day prednisone equiv. dose, then taper	discontinue permanently
	New-onset moderate to severe neurologic signs or symptoms		withhold dose, resume treatment when adverse reaction improves to Grade 0 or 1.
**motor or sensory neuropathy**	Grade 3-4		discontinue permanently; consider discontinuing ipilimumab only
	Grade 2		The next dose of ipilimumab should be delayed if the event of a Grade 2 sensory or motor neuropathy is considered related to ipilimumab. Upon improvement to Grade 1 or less, treatment with ipilimumab may be resumed.

**other**	Grade 3 adverse reaction<br>first occurrence<br><br>recurrence of same grade 3 AEs		withhold dose, resume treatment when adverse reaction improves to Grade 0 or 1.<br>discontinue permanently
	Life threatening or grade 4		discontinue permanently
	Grade 3 myocarditis		discontinue permanently
	Requirement for ≥10 mg/day prednisone equivalent for > 12 weeks		discontinue permanently
	Persistent Grade 2-3 lasting > 12 weeks		discontinue permanently

* upper limit normal

5.4.8 *Dosing discontinuation criteria requiring patient withdrawal from the study treatment*

Dosing discontinuation criteria requiring subject withdrawal from the study treatment should follow the IB recommendations detailed in Table 6 (section 5.4.7). When an AE falls into the category "discontinue permanently", this requires withdrawal from study treatment.
Nivolumab and ipilimumab are to be permanently discontinued if any of the following criteria are met:

- Grade 3 drug-related uveitis, pneumonitis, bronchospasm, colitis / diarrhoea requires discontinuation of study treatment.
- Grade 3 drug-related endocrinopathies adequately controlled with only physiologic hormone replacement do not necessarily require discontinuation; guidelines for the respective endocrinopathies according to the IB as shown in Table 6 (5.4.7) must be followed.
- In cases where grade 4 drug-related endocrinopathies do not require study treatment discontinuation, study treatment has to be delayed until patients are adequately treated and symptoms have been resolved or are adequately controlled.
- Grade 3 drug-related laboratory abnormalities do not require treatment discontinuation except:
    - Grade 3 drug-related thrombocytopenia >7 days or associated with bleeding requires discontinuation
- Any AE, elevated liver enzymes as listed above in Table 6, laboratory abnormality, or intercurrent illness, which, in the judgment of the investigator, presents a substantial clinical risk to the patient with continued nivolumab or ipilimumab dosing leads to discontinuation of study treatment.
- Any grade 4 drug-related AE or laboratory abnormality, except for the following events, which do not require discontinuation:
    - Grade 4 neutropenia ≤7 days
    - Grade 4 lymphopenia or leukopenia
    - Isolated grade 4 amylase or lipase abnormalities that are not associated with symptoms or clinical manifestations of pancreatitis
    - Isolated grade 4 electrolyte imbalances/abnormalities that are not associated with clinical sequelae and are corrected with supplementation/appropriate management
- Infusion-related reactions: Discontinue study medication for severe and life-threatening infusion-related reactions. Interrupt or slow the rate of infusion in patients with mild or moderate infusion-related reactions.
- Treatment delays (both for drug- and non-drug-related reasons) are permitted for a maximum of 10 weeks (counted from date of the first missed dose). This includes dosing delays to manage drug-related AEs, such as prolonged steroid tapers. However, treatment delays of >4 weeks (counted from date of the first missed dose) require approval by the coordinating investigator (LKP). As the only exception, a delay beyond 10 weeks is permitted in case a trial site is not able to administer study treatment for pandemic reasons.
- Patients with treatment delays exceeding 10 weeks (corresponding to no dosing of nivolumab or nivolumab plus ipilimumab for 12 weeks) are not allowed to continue study treatment.
- If the treating physician is able to clearly relate an appearing AE as Ipilimumab-related, Ipilimumab can be discontinued with Nivolumab still being continued. If the AE is not clearly relatable to one or the other, both drugs have to be discontinued. Ipilimumab continuation without Nivolumab is not permitted.

During and following a subject's participation in the trial, the investigator should ensure that adequate medical care is provided to a subject for any AE including clinically significant laboratory values. The investigator should inform a subject when medical care is needed for intercurrent illness(es) of which the investigator becomes aware of. For treatment of AEs, the investigator should adhere to the recommendations of the Investigator Brochure.

6 DESCRIPTION OF TRIAL VISITS

The study period for an individual subject starts with study inclusion and continues until the end of study visit. There are safety visits on day 30 and day 100 after last study treatment conclusion.

6.1 Screening Visit

- informed consent for study participation
- confirmation of CUP diagnosis (diagnostic tests for primary search at first diagnosis have to be considered and documented, but do not have to be repeated)
- exclusion of favorable CUP subtypes
- TMB analysis
    - Either:

      Ordering of FFPE block (if not already at the MPZ, Institute of Pathology, Heidelberg, if not older ≤ 6 months at Screening
    - or:

      tumor biopsy, if initial FFPE is older than six months or no sufficient material is left for TMB analysis
    - or:

      already present by routinely performed panel sequencing of a FFPE block, which is not older ≤ 6 months at Screening

  In case one attempt to perform TMB analysis on a new specimen has failed due to insufficient tumor cell quantity or insufficient quality in the specimen, or a re-biopsy has failed or cannot be performed for clinical or technical reasons, resorting to a specimen not older ≤24 months is allowed as an exception.
- CT / MRI scan of thorax and abdomen (if clinically indicated also head and neck); if CT / MRI was performed on a routine basis prior to screening, test does not have to be repeated if not older than 28 days at study inclusion)
- Complete medical history (with special diligence towards autoimmune disease and smoking status)
- Physical examination, ECOG status
- Vital signs (height, weight, blood pressure, pulse, body temperature)
- 12-lead ECG
- left ventricular ejection fraction
- Lab draw (conducted always at the trial site):
    - Hematology: WBC count, hemoglobin, platelet count, differential count (neutrophils, monocytes, lymphocytes, other cells)
    - Serum chemistry panel: sodium, potassium, calcium, chloride, glucose, creatinine, total protein, total bilirubin (if elevated direct bilirubin as well), alkaline phosphatase, ALT, AST, uric acid, LDH, CRP

- Coagulation: INR, aPTT
- Viral serology: HIV, hepatitis B surface antigen (HBsAg), total hepatitis B core antibody (HBcAb), hepatitis C virus (HCV) antibody
- Thyroid-function testing: thyroid-stimulating hormone (TSH), free T3, free T4

- Highly sensitive pregnancy test (all women of childbearing potential), maximum three days prior to first IMP dose.
- Quantiferon test (all patients with clinical suspicion of tuberculosis)
- If subject consented to translational research, blood sample suitable for analysis of circulating tumor DNA (cfDNA), whole blood sample, serum sample, as well as saliva and stool sample for microbiome analysis

6.2 Combination therapy Visits

Table 7 Study visits 1st cycle

	Study visit **	Nivolumab 240 mg	Ipilimumab 1 mg/kg
Cycle 1 day 1	X (+ pregnancy test)	X	X
Cycle 1 day 8 (+/-1)	X		
Cycle 1 day 15 (+/-2)*	X	X	
Cycle 1 day 22 (+/-2)	X		
Cycle 1 day 29 (+/-2)*	X (+ pregnancy test)	X	
Cycle 1 day 36 (+/-2)	X		

*Interval between two nivolumab infusions must not be less than 12 days

**The study visit includes:

- AE documentation (Medical history since last visit)
- Physical examination, ECOG status
- Vital signs (weight, blood pressure, pulse)
- Lab draw*** WBC count, hemoglobin, platelet count, differential count (neutrophils, monocytes, lymphocytes, other cells), sodium, potassium, calcium, chloride, glucose, creatinine, total protein, total bilirubin (if elevated direct bilirubin as well), alkaline phosphatase, ALT, AST, uric acid, LDH, CRP, TSH (free T3 and free T4 in case TSH is abnormal) and a monthly highly sensitive serum pregnancy test at day 1 and on day 29 (+/-2) of cycle 1 only. If a highly sensitive serum pregnancy test has been done in the screening phase within 3 days of IMP administration, the pregnancy test on day 1 is not required. In all subsequent treatment cycles the pregnancy test on day 1 (or the day before) is mandatory.
- *** on infusion treatment days blood draw for laboratory values on the previous day is allowed. The blood samples, including cycle 1 day 8, day 22 and day 36, may be taken

by external hospitals or practices not registered as trial sites as long as these facilities carrying out the analyses are appropriately certified and the health of the subject is not endangered. The lab values have to be available and checked prior to administration of study medication. Surveillance for Immune-Related Adverse Events (irAEs) (see below)

Study visits from 2nd cycle on (see table 8):

Table 8 Study visits from 2nd cycle #

	Study visit **	Nivolumab 240 mg	Ipilimumab 1mg/kg
Cycle n day 1	X (pregnancy test)	X	X
Cycle n day 15 (+/-2)*	X	X	
Cycle n day 29 (+/-2)*	X	X	

Check the foot notes of table 7 for further details. In case subjects discontinue only Ipilimumab and continue with Nivolumab as monotherapy, the same study visit schedule applies.

6.3 Surveillance for immune-related Adverse Events (irAEs)

Blocking PD-1 and CTLA-4 function may permit the emergence of immunological side effects, i.e. ranging from susceptibility to infections to auto-reactive T cells and resultant clinical autoimmunity. This phenomenon is well known for immunotherapies utilizing Nivolumab and Ipilimumab. Severe rash, infections without adequate response of increased leukocyte counts or infections with higher frequency than clinically expected, vitiligo, prolonged diarrhea, colitis, uveitis, episcleritis, hepatitis, and hypopituitarism are potential drug-related, presumptive immune events, here now termed irAEs. Respective paragraphs in the investigators' brochure outline the expected immunological side effects of Nivolumab and Ipilimumab.

For the purposes of this study, an irAE is defined as an AE of unknown etiology associated with drug exposure and consistent with an immune phenomenon. Efforts should be made to rule out neoplastic, metabolic, toxic or other etiologic causes prior to labeling an AE an irAE. Serological, immunological, and histological (biopsy) data should be used to support the diagnosis of an immune-mediated toxicity. Suspected IRAEs must be documented on an AE or SAE form.

Subjects should be informed of and carefully monitored for evidence of clinically significant systemic irAE (e.g., opportunistic infections, systemic lupus erythematosus-like diseases, repeated or prolonged infections, liver failure) or organ-specific irAE (e.g. severe rash, colitis, uveitis, hepatitis or thyroid disease). If an irAE is noted, appropriate work-up (including biopsy if possible) should be performed, and medical therapy may be considered if clinically necessary.

It is unknown if systemic corticosteroid therapy has an attenuating effect on PD-1 and/or CTLA-4-antagonist activity. If utilized, corticosteroid therapy should be individualized for each patient. There are specific guidelines in place for immunological side effects of Nivolumab and/or Ipilimumab therapy requiring corticosteroid treatment.

6.4 Staging visits

CT or MRI of chest and abdomen (and head and neck if clinically indicated) have to be performed regularly every 12 weeks (+/- 10 days). If the study treatment is followed according to the protocol, the staging visits are scheduled after every second cycle (cycle 2, 4, 6, 8, 10…). If the study treatment is delayed e.g. due to side effects of nivolumab and/or ipilimumab, the staging visit should not be postponed and routinely performed every 12 weeks (+/- 10 days) to monitor disease progression nevertheless.

Additionally, at these staging visits the following procedures will be performed:

- ECG
- Physical examination, ECOG status
- Vital signs (weight, blood pressure, pulse)
- Concomitant medication
- AE documentation (Medical history since last visit)
- Routine blood sampling*
- Collection of blood samples at the trial site suitable for analysis of circulating tumor DNA (cfDNA), if subject consented to translational research.

* routine blood samples do not have to be collected, if lab results are present from a study treatment visit or external facility, which are not older than 7 days on the date of the staging visit.

6.5 End of Treatment Visit (EoT)

The End of Treatment Visit for a safety follow-up takes place on day 30 (± 7) after the last treatment within the trial. The appointment is to be preponed if the patient starts another anti-cancer therapy.

The following tests should be performed:

- AE documentation (Medical history since last visit)
- Physical examination, ECOG status
- Vital signs (weight, blood pressure, pulse)
- Lab draw (conducted always at the trial site): WBC count, hemoglobin, platelet count, differential count (neutrophils, monocytes, lymphocytes, other cells), sodium, potassium, calcium, chloride, glucose, creatinine, total protein, total bilirubin (if elevated direct bilirubin as well), alkaline phosphatase, ALT, AST, uric acid, LDH, CRP, TSH (free T3 and free T4 in case TSH is abnormal)
- Blood sample suitable for analysis of circulating tumor DNA (cfDNA) (central laboratory Heidelberg), whole blood sample, serum sample, if subject consented to translational research
- CT or MRI of thorax and abdomen (and if clinically indicated head and neck) for determination of remission status in case no CT or MRI for remission status has been performed after the last administration of study drug, to determine the need for subsequent, off-study treatment.

6.6 Safety Phone Call

The Day +100 Safety phone call takes place on day 100 (+/- 7) after the last study treatment within the trial, irrespective of whether new treatment has been initiated or not. AEs are to be recorded until day +100 after the final dose of study treatment.

6.7 Follow-up phase

Thereafter, subjects will be contacted by the physician by phone every 3 (± 1) months for survival follow-up. Subjects who discontinue for reason other than PD but who do not withdraw consent to follow up will be assessed for progression as described during the Treatment Period (including scans) until disease progression (refer to Section 4.5.6). After disease progression, these subjects will continue with survival follow up.

The investigator will continue to observe all subjects (also withdrawals) because of intolerable AEs/ SAEs until the findings have been clarified or became stable.

6.8 Study visits in case of trial site impairment in case of a pandemic

If a trial site is incapable of administering therapy due to a pandemic, the administration of study treatment can be transferred to another trial site after obtaining consent from the coordinating investigator. Documentation in form of a note-to-file is required.

Also, in case of a pandemic, CT/MRI imaging are allowed to be delegated to outside hospitals or practices. However, assessment according to RECIST criteria has to be performed at the center as soon as circumstances allow. Documentation is required. If external facilities are used exceptionally, they have to be certified to perform the analyses accordingly.

In case of a pandemic emergency all study visits not due on treatment days are allowed to be conducted by phone, provided this is justified by the subject´s clinical status. In this case a physical examination has to be skipped. Documentation for the reason to conduct the study visit via phone and the phone visit itself in a note-to-file is mandatory.

7 METHODS OF DATA COLLECTION

7.1 TMB Value analysis

Assessment of tumor mutational burden from FFPE tumor tissue (without normal reference tissue for germline variant subtraction) will be performed by using the TruSight Oncology 500 (TSO500) panel from Illumina (San Diego, USA), which encompasses a TMB target region size of 1.34 Mb. DNA extraction will be performed using the MaxWell DNA LEV purification system (Promega, Mannheim, Germany). In brief, tumor-enriched regions will be manually macrodissected from 2-4 thin FFPE tissue slices and incubated with Proteinase-K containing buffer over night at 65°C. After automated DNA extraction on the Maxwell RSC instrument (Promega), DNA will be eluted in 40µl low-salt buffer. DNA concentration will be measured fluorimetrically using QuBit 4 instrumentation (Thermo Fisher Scientific, Carlsbad, USA) and DNA integrity will be determined by qPCR testing (RNAseP assay, Thermo Fisher Scientific). For TSO500 library preparation, a minimum of 40ng DNA as measured by RNAseP assay (maximum 120ng) will be used. DNA fragmentation will be performed on a Covaris ME220 instrumentation using the following settings: Peak Indicent Power: 450 watts, Duty Factor 30%, Cycles per Burst: 200, Treatment Time: 250 sec, Temperature: 7°C. After end repair of sheared DNA fragments, unique molecular identifiers will be ligated to each single molecule followed by incorporation of sequencing barcode adapters by 15 PCR cycles. After cleanup, two rounds of target capture hybridizations will be performed at 57°C. After purification, the enriched library will be amplified, quality controlled on a Bioanalyzer instrumentation (Agilent, Santa Clara, USA) and quantified using the KAPA LibraryQuant Kit (Roche). Sequencing of up to 8 TSO500 libraries will be performed on a NextSeq 500 (Illumina, San Diego, USA) using High output flow cells and v2 chemistry. Data analysis including raw data processing, alignment to hg19, variant calling and TMB estimation will be performed using a locally installed TSO500 docker image. ≥ 12 mutations / Mb are considered TMB-high (Buchhalter et al., 2018).

The analysis will be performed at the MPZ, Institute of Pathology, University of Heidelberg. Results will be available 10 working days after the sample was received at the MPZ.

Blood based TMB measurement will utilize the bTMB-assay from Illumina on either Highseq 2500 or Novaseq machines (Illumina, Inc). 20 ml of peripheral venous blood collected in Streck-tubes are required (minimum of 4 ml of plasma). To isolate plasma, whole blood is centrifuged (1,600 × g for 10 minutes at 10°C), and the resulting supernatant is clarified by additional centrifugation (3,220 × g for 10 minutes at 10°C). Clarified plasma will be stored at 2°C for immediate further processing or stored at −80°C. 30 ng of extracted cfDNA will be used to prepare sequencing libraries. Enrichment of the specific target regions will be achieved by hybrid capture (Agilent Technologies, Inc.), pooled, and subsequently sequenced by paired-end synthesis. Minimal coverage is 3000x, average coverage is 5000x.

7.2 Microbiome Analysis

Vials for saliva and stool samples will be provided and analysed by the German Cancer Research Center (DKFZ) Heidelberg. Vials including shipment boxes will be handed over to the respective patients and send after sampling by the patient to the processing laboratory:

Dr. Christoph Stein-Thöringer, Abteilung Mikrobiom und Krebs, Deutsches Krebsforschungszentrum, Im Neuenheimer Feld 280, 69120 Heidelberg

7.3 Safety Parameters

7.3.1 *Adverse Events*

For definition see 8.1.1.

Adverse events will be interrogated for at each contact between the responsible investigator and the study subject. Furthermore, all pathological and clinically relevant findings in physical and neurological examinations, vital signs, 12-lead ECGs, clinical chemistry, hematology, and clotting will be documented as adverse events

Wherever possible, adverse events will be reported on the basis of CTCAE v5.0.

Adverse events will be reported with subject ID, start and end date, description, grading, seriousness, relationship, action taken and outcome.

Vital signs:
Vital signs (pulse rate, systolic and diastolic blood pressure and body temperature) determined on predefined study days will be documented as numerical values on appropriate eCRF pages. Furthermore, vital signs may be recorded at any time, if medically imperative for clarification of clinical signs and symptoms. Pathological and clinically relevant findings will be documented as adverse events/ serious adverse events.

12-lead ECG
Only pathological and clinically relevant findings in 12-lead ECG determined on predefined study days will be documented on appropriate eCRF pages. No records of numerical values, such as heart rate, particular times and intervals will be collected.

12-lead ECG may be recorded at any time at discretion of the responsible investigator, if medically imperative for clarification of clinical signs and symptoms. Pathological and clinically relevant findings will be documented as adverse events/ serious adverse events.

Clinical chemistry, hematology and clotting:
The parameters determined on the predefined study days are listed in detail in 6.3.

After collection the samples will immediately be delivered to the central laboratory for respective determinations. All parameters will be documented on appropriate eCRF pages.

Further laboratory parameters may be determined at any time during the study at discretion of the responsible investigator. Pathological and clinically relevant findings will be documented as adverse events/ serious adverse events.

7.4 Efficacy Parameters

7.4.1 *Progression-free Survival (PFS)*

PFS is defined as the time from start of therapy to the first observation of disease progression or death due to any cause. If a subject is lost to follow up, progression-free survival is censored at the time of last documented efficacy.

7.4.2 *Overall Survival (OS)*

OS is defined as the time from start of therapy to death due to any cause. If a subject is lost to follow up, overall survival time is censored at the time of last contact.

8 ADVERSE EVENTS

8.1 Definitions

8.1.1 *Adverse Event*

According to ICH-GCP, an adverse event (AE) is defined as follows: Any untoward medical occurrence in a subject administered a pharmaceutical product and which does not necessarily have a causal relationship with this treatment. An AE can therefore be any unfavourable and unintended sign (including an abnormal laboratory finding), symptom, or disease temporally associated with the use of a medicinal (investigational) product, whether or not related to the medicinal (investigational) product.

An AE may be:

- New symptoms/medical conditions
- New diagnosis
- Changes of laboratory parameters
- Intercurrent diseases and accidents
- Worsening of medical conditions/ diseases existing before clinical start
- Recurrence of disease
- Increase of frequency or intensity of episodic diseases.

A pre-existing disease or symptom will not be considered an adverse event unless there will be an untoward change in its intensity, frequency or quality. This change will be documented by an investigator.

Surgical procedures themselves are not AEs; they are therapeutic measures for conditions that require surgery. The condition for which the surgery is required may be an AE. Planned surgical measures permitted by the clinical trial protocol and the condition(s) leading to these measures are not AEs, if the condition leading to the measure was present prior to inclusion into the trial. In the latter case the condition should be reported as medical history.

AEs are classified as "non-serious" or "serious".

All Adverse events will be reported with start and stop date and grade at occurrence on the eCRF.

Laboratory abnormalities:

All laboratory test results captured as part of the study should be recorded following institutional procedures. Test results that constitute SAEs should be documented and reported as such.

The following laboratory abnormalities should be documented appropriately:

- any laboratory test result that is clinically significant or meets the definition of an SAE
- any laboratory abnormality that required the participant to have study drug discontinued or interrupted
- any laboratory abnormality that required the patient to receive specific corrective therapy.

It is expected that wherever possible, the clinical rather than laboratory term would be used by the reporting investigator (e.g., anemia versus low hemoglobin value).

Laboratory abnormalities that do not meet any of the above criteria for an adverse event should not be reported as adverse events. A grade 3 or higher event as per CTCAE does not automatically indicate a serious adverse event (SAE) unless it meets the definition of seriousness as defined below.

8.1.2 *Serious Adverse Event*

A serious adverse event (SAE) is one that at any dose:

- Results in death
- Is life-threatening (the term life-threatening refers to an event in which the subject was at risk of death at the time of event and not to an event which hypothetically might have caused death if it was more severe)
- Requires hospitalisation or prolongation of existing hospitalisation*
- Results in persistent or significant disability/ incapacity
- Is a congenital anomaly/ birth defect or
- Is otherwise medically relevant

Disease progression of any outcome, including its signs and symptoms, is documented in the eCRF and will be reported together with all other AEs in the Final Study Report. Disease progression of any outcome, including its signs and symptoms does not require immediate reporting, i.e. expedited reporting as SAE, if considered unrelated to study therapy by the investigator.

Moreover, please note that the following cases of hospitalizations are NOT deemed as SAEs:

- Hospitalization aiming exclusively at diagnostic measures or due to technical, practical or social reasons
- Elective or pre-planned treatment for pre-existing conditions that are unrelated to the indication under study
- Social reasons and respite care in the absence of deterioration of symptoms related to the indication under study
- Emergency outpatient treatment (without hospital admission) is not considered an SAE if it does not fulfill any of the above listed definitions

Medical and scientific judgement should be used in deciding whether expedited reporting is appropriate in other situations - such as important medical events that may not be immediately life threatening or result in death or hospitalisation but may jeopardise the subject or may require intervention to prevent one of the other outcomes listed above. These should also usually be considered serious (examples of such events are intensive treatment in an emergency room or at home for allergic bronchospasm; blood dyscrasias or convulsions that do not result in hospitalisation; or development of drug dependency or drug abuse).

In particular the following events have to be handled as SAEs:

Although pregnancy, overdose, potential drug-induced liver injury (DILI), are not always serious by regulatory definition, these events will be handled as SAEs. Suspected transmission of an infectious agent (e.g., pathogenic or nonpathogenic) via the study treatment is an SAE.

Pregnancy

If, following initiation of any IMP, it is subsequently discovered that a subject is pregnant or may have been pregnant at the time of IMP exposure, both IMPs will be permanently discontinued in an appropriate manner e.g., dose tapering if necessary for participant.

The investigator must immediately notify the PV-Department at the KKS Heidelberg of this event via the Pregnancy Form in accordance with SAE reporting procedures.

Protocol-required procedures for study discontinuation and follow-up must be performed for the participant.

Follow-up information regarding the course of the pregnancy, including perinatal and neonatal outcome and, where applicable, offspring information will be reported on a specific Pregnancy Form.

Any pregnancy that occurs in a female partner of a male study participant should be reported to BMS. Information on this pregnancy will be collected on the Pregnancy Form. In order for Sponsor or designee to collect any pregnancy surveillance information from the female partner, the female partner must sign an informed consent form for disclosure of this information.

Overdose and other Safety Considerations

An overdose is defined as the accidental or intentional administration of any dose of a product that is considered both excessive and medically important. All occurrences of overdose must be reported as an SAE.

Other safety considerations include any significant worsening noted during interim or final physical examinations, electrocardiograms, X-rays, and any other potential safety assessments, whether or not these procedures are required by the protocol, should also be recorded as a non- serious or serious AE, as appropriate, and reported accordingly.

Potential Drug Induced Liver Injury (DILI):

All occurrences of potential DILIs will be reported as SAEs.

Potential drug induced liver injury is defined as:

1) AT (ALT or AST) elevation > 3 times upper limit of normal (ULN)

AND

2) Total bilirubin > 2 times ULN, without initial findings of cholestasis (elevated serum alkaline phosphatase)

AND

3) No other immediately apparent possible causes of AT elevation and hyperbilirubinemia, including, but not limited to, viral hepatitis, pre-existing chronic or acute liver disease, or the administration of other drug(s) known to be hepatotoxic.

8.1.3 *Adverse (Drug) Reaction*

All noxious and unintended responses to a medicinal product related to any dose should be considered adverse (drug) reactions.

8.1.4 *Expectedness*

An ‘unexpected’ adverse reaction is one the nature or severity of which is not consistent with the applicable product information, e.g., Investigator’s Brochure (IB) for Nivolumab and Ipilimumab. Furthermore, reports which add significant information on specificity or severity of a known adverse reaction constitute ‘unexpected’ events.

8.1.5 *Suspected Unexpected Serious Adverse Reaction (SUSAR)*

SAEs that are both ‘suspected’, i.e., possibly related to IMP and ‘unexpected’, i.e., the nature and/ or severity of which is not consistent with the applicable product information are to be classified as Suspected Unexpected Serious Adverse Reactions (SUSARs).

In case, either the investigator who primary reported the SAE or the second assessor classify the SAE as ‘suspected’ (i.e. either as (definitely) or probably or possibly related to the use of at least one of the IMPs) and the SAE is ‘unexpected’ (in respect of the IMP, for which a positive causality assessment was provided) it will be categorized as a SUSAR.

All SUSARs are subject to an expedited reporting to the responsible ethics committee, the competent higher federal authority (i.e. PEI) and to all participating investigators. (For details on known possible adverse events see Investigator’s Brochure of Nivolumab and Ipilimumab).

The grading of AEs in this trial will be carried out on the basis of the 5-grade scale defined in the CTCAE v5.0:

Grade 1:	Mild or asymptomatic AE
Grade 2:	Moderate AE
Grade 3:	Severe AE
Grade 4:	Life threatening AE or AE causing disablement
Grade 5:	Death related to AE

The grading of all AEs listed in the CTCAE v5.0 will be based on the information contained therein. The grading of all other AEs, i.e., those which are not listed in the CTCAE v5.0 will be performed by a responsible investigator, based on definitions given above.

8.1.6 *Relationship and Outcome of AEs*

The investigator will evaluate each AE that occurred after administration of the IMP regarding the **relationship** with the administration of the IMP:

Definitely related:	There is a reasonable possibility that the event may have been caused by the IMP. A certain event has a **strong temporal relationship** and an alternative cause is unlikely.
Probably:	An AE that has a reasonable possibility that the event is likely to have been caused by the IMP. The AE has a **timely relationship** and **follows a known pattern of response**, but a potential alternative cause may be present.
Possibly:	An AE that has a reasonable possibility that the event may have been caused by the IMP. The AE has a **timely relationship** to the IMP; **however, the pattern of response is untypical**, and an alternative cause seems more likely, or there is significant uncertainty about the cause of the event.
Unlikely:	Only a remote connection exists between the IMP and the reported adverse event. Other conditions including concurrent illness, progression or expression of the disease state or reaction of the concomitant medication appear to explain the reported adverse event.

Not related:	An AE that does not follow a reasonable temporal sequence related to the IMP and is likely to have been produced by the subject's clinical state, other modes of therapy or other known etiology.
Not assessable:	There is insufficient or incomplete evidence to make a clinical judgement of the causal relationship.

All subjects who have reportable AEs, whether considered associated with the use of the trial medication or not, must be monitored to determine the **outcome**. The clinical course of the AE will be followed up until resolution or normalisation of changed laboratory parameters or until it has changed to a stable condition. This also holds for on-going AEs/SAEs of withdrawn subjects, in case they agreed to be further contacted.

The outcome of an AE at the time of the last observation will be classified as:

Recovered / resolved:	All signs and symptoms of an AE disappeared without any sequels at the time of the last interrogation.
Recovering / resolving:	The intensity of signs and symptoms has been diminishing and / or their clinical pattern has been changing up to the time of the last interrogation in a way typical for its resolution.
Not recovered/not resolved:	Signs and symptoms of an AE are mostly unchanged or worsened at the time of the last interrogation.
Recovered / resolved with sequel:	Actual signs and symptoms of an AE disappeared but there are sequels related to the AE.
Fatal:	Resulting in death. If there are more than one adverse event only the adverse event leading to death (possibly related) will be characterized as 'fatal'.
Unknown	The outcome is unknown or implausible and the information cannot be supplemented or verified.

The action taken with the IMP will be assigned to one of the following categories:

Dose not changed:	No change in the dose of the IMP.
Drug withdrawn:	Discontinuation of the IMP.
Unknown:	The information is unknown or implausible and it cannot be supplemented or verified
Not applicable:	The question is implausible (e.g. the subject is dead).

The term "countermeasures" refers to the specific actions taken to treat or alleviate adverse events or to avoid their sequels. Following categories will be used to categorise the countermeasures to adverse events:

None:	No action taken.
Drug treatment:	Newly-prescribed medication or change in dose of a medication.
Others:	Other countermeasures, e.g. an operative procedure.

8.2 Treatment of AEs / SAEs

Treatment for AEs / SAEs should adhere to the recommendations of the investigator brochure.

8.3 Period of Observation and Documentation

All AEs reported by the subject or detected by the investigator, will be collected during the trial and must be documented on the appropriate pages of the CRF. AEs must also be documented in the subject's medical records.
AEs will be ascertained by the investigators using non-leading questions, noted as spontaneously reported by the subjects to the medical staff or observed during any measurements on all study days. The observation period begins with signature of informed consent and ends 100 days after the last administration of study treatment with either substance, whichever comes last.
All AEs, whether related or not related to study drug, are collected, including those thought to be associated with protocol-specified procedures. The investigator should report any SAE occurring after these aforementioned time periods, which is believed to be related to study drug or protocol-specified procedure.

8.4 Reporting of Serious Adverse Events by Investigator

All subjects who have AEs, whether considered associated with the use of the trial medication or not, must be monitored to determine the outcome. The clinical course of the AE will be followed up until resolution or normalization of changed laboratory parameters or until it has changed to a stable condition.

All SAEs must be reported by the investigator to the PV-Department of KKS Heidelberg within 24 hours after the SAE becomes known using the "Serious Adverse Event" form.

The fax number of the PV-Department of KKS Heidelberg is: **+49 (0)6221-56-33725.**

All SAE reports are forwarded to BMS by the PV-Department of the KKS Heidelberg (Worldwide.Safety@bms.com).

The initial report must be as complete as possible including details of the current illness and (serious) adverse event and an assessment of the causal relationship between the event and the trial medication.

If only limited information is initially available, follow-up reports might be necessary. If an ongoing SAE increases in its intensity or relationship to study drug or if new information becomes available, a follow-up SAE report should be sent within 24 hours to the PV-Department at the KKS Heidelberg using the same procedure used for transmitting the initial SAE report. All SAEs should be followed up to resolution or stabilization.

8.5 Expedited Reporting

All SAE will be subject to a second assessment by a designated person, who will be independent from the reporting investigator and the KKS Heidelberg. The designated person for the present trial, referred to as the second assessor is:

Prof. Dr. Alwin Krämer, Clinical Cooperation Unit Molecular Hematology/Oncology, German Cancer Research Center and Dept. Of Internal Medicine, University of Heidelberg, Heidelberg, Germany

PD Dr. Tilmann Bochtler (deputy), Clinical Cooperation Unit Molecular Hematology/Oncology, German Cancer Research Center and Dept. Of Internal Medicine, University of Heidelberg, Heidelberg, Germany

Dr. Julia Meissner, senior physician, specialist in internal medicine, hematology/oncology, Dept. Of Internal Medicine, University of Heidelberg, Heidelberg, Germany

The second assessor will fill out a 'Second Assessment Form' for each SAE and send it back per fax to the responsible person at the KKS Heidelberg within 48 hours. The fax number of the PV-Department of KKS Heidelberg is: **+49 (0)6221-56-33725**. The 'Second Assessment Form' will contain the following information:

I) assessment of relationship between SAE and IMP

II) assessment of expectedness of SAE (derived from IB)

III) assessment of relationship between SAE and the underlying disease

IV) Statement if the benefit/ risk assessment for the trial did change as a result of SAE.

SUSARs are to be reported to the ethics committee, competent higher federal authority (i.e. PEI) and to all participating investigators within regulative defined timelines, i.e. they are subject to an expedited reporting.

The expedited reporting will be carried out by the PV-Department of KKS Heidelberg.

Details concerning the concerning the SAE management including the reporting of SUSARs will be described in a separate document "Safety Manual".

8.6 Safety management

Emergency equipment has to be held immediately available during and after nivolumab and ipilimumab infusions. Vital signs (blood pressure, pulse, temperature) have to be checked in advance of infusion start. A physician has to be present in the treatment rooms during and after infusion. Patients should remain within the treatment rooms for at least 30 minutes after the last nivolumab or ipilimumab infusion, respectively. The laboratory draws as required at study visits will ensure close monitoring of potential autoimmune related disorders and other side effects. Adequate medical care has to be provided to the patient for any adverse event, including clinically significant laboratory values. If the investigator becomes aware of an intercurrent illness, the investigator / institution has to inform the patient about the needed medical care. All clinical trial information has to be recorded, handled, and stored in a way that allows its accurate reporting, interpretation and verification.

9 STATISTICAL PROCEDURES

9.1 Sample Size Calculation

This is a non-randomized biomarker trial. Tumor mutational burden (TMB) is considered as biomarker. Subjects showing high TMB are considered biomarker-positive. A total of 194 subjects with 191 events are required to detect a hazard ratio of 0.65 for biomarker positive vs biomarker negative subjects with 80% power at the two-sided significance level of 5%. Median progression-free survival in the studied subject population is assumed to be 2.3 months, and 15% of subjects are expected to be biomarker-positive. Biomarker-positive subjects are expected to have a favorable prognosis. Assuming a hazard ratio of 0.65 for biomarker-positive versus biomarker-negative subjects and exponentially distributed survival, median survival times are 2.18 and 3.35 months for biomarker-negative and biomarker-positive subjects, respectively. Subjects will be recruited in a 1:1 ratio, i.e. biomarker-positive subjects will be enriched, which means that approximately 700 subjects need to be assessed for their TMB status. There will be a 24 months recruitment period and a minimal follow-up time of 12 months, i.e., a total trial duration of 36 months, which allows observing the necessary number of events under the above stated assumptions. An interim analysis is planned as described in 9.5. Sample size was calculated with R package gsDesign (Keaven and Anderson, 2016) according to Lachin and Foulkes (1986).

9.2 Analysis Variables

Primary endpoint is progression-free survival (PFS), which is defined as the time from treatment start to the first occurrence of disease progression, as assessed by the investigator according to Response Evaluation Criteria in Solid Tumors, Version 1.1 (RECIST v1.1), or death from any cause, whichever occurs first. Secondary endpoints comprise overall survival (OS), overall response rate (ORR) and duration of clinical benefit (DCB). OS is defined as time from treatment start to death from any cause. Overall response rate (ORR) is defined as the proportion of subjects who exhibit a CR or PR to study treatment on two consecutive occasions ≥ 4 weeks apart. Duration of clinical benefit (DCB) is defined as the time from the first occurrence of a CR, PR or SD after treatment start until disease progression or death from any cause, whichever occurs first. Responses will be determined by the investigator according to RECIST v1.1.

9.3 Definition of Trial Population to be Analyzed

The primary analysis will be performed for the full-analysis set (FAS) analysis set which comprises all subjects with valid biomarker status starting trial medication. The safety set will comprise all registered subjects who have received study medication at least once.

9.4 Statistical Methods

The primary analysis will be performed by testing the null hypothesis of no difference in PFS between both biomarker groups using a log-rank test at a significance level of 5%. Secondary analyses of the primary endpoint comprise a multivariable Cox regression model including relevant prognostic factors. Sensitivity analysis will investigate potential centre effects. The secondary endpoint OS will be analyzed similarly to PFS. Incidence and severity of adverse events will be analyzed for the safety population.

A detailed biometric analysis will be defined in the statistical analysis plan which has to be authorized before database closure by the biometrician, the sponsor, and the PI.

9.5 Interim Analyses

An interim analysis for futility is performed at the time point when approximately 33% of the total number of events are expected, which is 12 months after trial initiation with 63 events being expected to be observed and half of the subjects being expected to be enrolled (Lachin, 2005). Continuation of the trial should be discussed if at the interim analysis the conditional power (CP) for the estimated treatment effect is below 10%, which is equivalent to a hazard ratio of 0.88 at the boundary. In detail, a group sequential design is used to address futility at the interim analysis, using the Hwang-Shih-DeCani spending function with gamma of 1.95 for futility bound. Type II error at interim and final analysis is 11% and 9%, respectively, ensuring an overall type II error of 20%. Calculations have been performed with R package gsDesign (Keaven and Anderson, 2016).

If data at the interim analysis indicates a considerable proportion of patients being lost-to-follow, enrollment of additional patients will be discussed.

9.6 Final Analysis

The final analysis will be performed 36 months after trial initiation, i.e. 12 months after the last patient has been enrolled, at which time point the required number of events is expected to be observed. If the number of events at this time point is substantially lower, the follow-up period of the trial will be prolonged.

10 DATA MANAGEMENT

10.1 Data Collection

In this trial a clinical data management system is used for data collection using an electronic CRF (eCRF) for remote data entry (RDE).

All entries in the eCRF must be verifiable by source documents. In advance exceptions to this rule can be defined by the sponsor. A detailed list will be provided in the Investigator Site File. Regardless, there must be a minimum documentation, which provides information on study participation and includes all medical information necessary for appropriate medical care outside of the clinical trial in the patient record.

In addition, source documents must mention that the subject has been included in an investigational study. Finally, there must be no data that are inconsistent between eCRF and source documents.

All protocol-required information collected during the trial must be entered by the investigator or a designated representative into the eCRF. Patient data will be documented pseudonymously. The investigator or a designated representative should complete the eCRF pages as soon as possible after the information is collected, preferably within 2 weeks after the study visit. Any pending entries must be completed immediately after the final examination. Explanation should be given for all missing data.

The investigator is responsible for ensuring that all sections of the eCRF are completed correctly. Any errors should be corrected in the eCRF and a reason for change has to be entered. The correctness of all entries in eCRF will be confirmed by dated electronic signature of the responsible investigator. The correctness of all entries in the eCRF has to be confirmed by dated electronic signatures of the responsible investigator. The time points and frequency of electronic signatures will be defined in the study specific document 'eCRF specification'.

The following data will not be entered into the eCRF but elsewhere and transferred to the biometrician for analyses, if required:

- genomic data from tumor tissue
- cfDNA derived from blood samples
- Microbiome analysis

10.2 Data Handling

Data entries will undergo an automatical online check for plausibility and consistency, which are to be defined in the trials data validation plan (DVP). In case of implausibilities, 'warnings' will be produced during data entry (edit checks). A responsible investigator or a designated representative will be obliged either to correct the implausible data or to confirm its authenticity and to give appropriate explanation. The responsible data manager will check all explanations and resolves the warnings if the explanation is appropriate. The responsible monitor can generate special questions (monitor query), that will be send back to the responsible investigator. The investigator or a designated representative will have to answer them all. The responsible monitor will check all answers and resolves the monitor queries if the answer is appropriate. Analog queries can be used by the data manager (dm query).

The correctness of all entries in the eCRF has to be confirmed by dated electronic signatures of the responsible investigator. The time points and frequency of electronic signatures will be defined in the study specific document “eCRF specification”.

All missing data or inconsistencies will be reported back to the site and have to be clarified by the responsible investigator prior to database lock. If no further corrections are to be made in the database it will be declared locked and used for statistical analysis.

Details of data handling will be described in the trial data management plan (DMP).

All data management activities will be done according to the current SOPs of the KKS.

10.3 Archiving of Essential Documents and Data

The investigator(s) will archive all trial data (source data and Investigator Site File (ISF) including subject identification list and relevant correspondence according to the section 4.9 of the ICH Consolidated Guideline on GCP (E6) and to local law or regulations.

The sponsor or other owner like investigators of the data shall retain all other documentation pertaining to the trial for at least 10 years according to the §13 of the German GCP-Ordinance. These procedures shall include:

- the protocol including the rationale, objectives and statistical design and methodology of the trial, with conditions under which it is performed and managed, and details of the investigational product used.
- standard operating procedures
- all written opinions on the protocol and procedures,
- final report,
- electronic case report forms in readable format,
- audit certificate(s), if available.
- all other relevant documents of the trial master file, according to the ICH-GCP guideline

Any change of data ownership shall be documented. All data shall be made available if requested by relevant authorities.

11 ETHICAL AND LEGAL ASPECTS

11.1 Good Clinical Practice

The procedures set out in this trial protocol, pertaining to the conduct, evaluation, and documentation of this trial, are designed to ensure that all persons involved in the trial abide by the integrated addeddum of the ICH hamonised tripartite guideline on Good Clinical Practice (ICH-GCP E6 R2) and the ethical principles described in the applicable version of the Declaration of Helsinki. The trial will be carried out in accordance with local legal and regulatory requirements.

11.2 Legal bases

The study has to be conducted in compliance with the protocol, ICH-GCP E6 R2 and the applicable regulatory requirements.

11.2.1 *Declaration of Helsinki*

The study will be carried out in conformity with the “Ethical principles for medical research involving human subjects” of the 18th World Medical Association General Assembly in Helsinki (1964), and amended by the 29th, 35th, 41st, 48th, 52nd and 59th, World Medical Association General Assemblies (Tokyo 1975, Venice 1983, Hong Kong 1989, Somerset West 1996, Edinburgh 2000 and Seoul 2008) and the Note of Clarification on Paragraph 29 added by the World Medical Association General Assembly, Washington 2002 and the Note of Clarification on Paragraph 30 added by the WMA General Assembly, Tokyo 2004, Fortaleza October 2013. The applicable version for the respective country will be taken into consideration.

11.2.2 *Other Legal Bases*

The other legal bases of this clinical trial are as follows:

- ICH Topic E6 R2, Guideline for Good Clinical Practice, current step 4 version, November 2016
- Directive 2001/20/EC (April 4, 2001)
- Commission Directive 2005/28/EC (April 8, 2005)
- National regulatory requirements/guidelines of the participating countries concerning Clinical Trials [e.g. federal drug law (AMG), GCP ordinance (GCP-Verordnung), Medical device law (MPG)]
- General national regulatory requirements, e.g. Bundesdatenschutzgesetz (BDSG)
- General European regulatory requirements, e.g. General Data Protection Regulation (GDPR)

The Coordinating Investigator and all investigators will be given an up-to-date investigator’s brochure containing full details of the status of the pre-clinical and clinical knowledge of the study medication. As soon as new information is obtained, an updated version will be supplied or an amendment added to the existing investigator’s brochure.

11.3 Approval of Trial Protocol and Amendments

Before the start of the trial, the trial protocol, informed consent document, and any other appropriate documents will be submitted to the independent Ethics Committee (EC) as well as to the competent authority (PEI).

A written favourable vote of the EC and an (implicit) approval by the competent authority are a prerequisite for initiation of this clinical trial. The statement of EC should contain the title of the trial, the trial code, the trial site, and a list of reviewed documents. It must mention the date on which the decision was made and must be officially signed by a committee member. This documentation must also include a list of members of the EC present on the applicable EC meeting and a GCP compliance statement.

The investigator *and the KKS Heidelberg* will keep a record of all communication with the EC and the regulatory authorities

Before the first subject is enrolled in the trial, all ethical and legal requirements must be met.

All planned substantial changes (see §10, (1) of German GCP-Regulation) will be submitted to EC and the competent authority in writing as protocol amendments. They have to be signed by the sponsor and biometrician and approved by the EC and the competent authority.

11.4 Notification of Regulatory Authorities

In addition to the approval by the competent authority (see 11.3) the clinical trial must also be notified to the competent authority before recruitment of the first subject (according to AMG §67).

The local regulatory authorities responsible for each particular investigator will be informed before the beginning, during and at the end of the trial according to the applicable regulations. Each investigator is obliged to notify his/ her local regulatory authority whereas the notification of the competent authority is the responsibility of the sponsor. Both responsibilities have been delegated to the KKS.

Substantial Amendments, interruption or premature end of the clinical trial need to be reported, too.

11.5 Subject Information and Informed Consent

Before being admitted to the clinical trial, the subject must consent to participate after being fully informed by the investigator or a designated member of the investigating team about the nature, importance, risks and individual consequences of the clinical trial and their right, to terminate the participation at any time.

The subject should also have the opportunity to consult the investigator, or a physician member of the investigating team about the details of the clinical trial. The informed consent to participate in the clinical trial may be withdrawn by the subject verbally in the presence of, or in written form directed to, the investigator or a physician member of the investigating team at any time during the trial. The subject must not entail any disadvantage therefor or be coerced or unduly influenced to continue to participate. Furthermore, the subject is not obligated to disclose reasons for the withdrawal of the consent.

If the subject has a primary physician, the investigator should inform him or her about the subject’s participation in the trial, provided the subject agrees hereto.

After reading the informed consent document, the subject must give consent in writing. The subject's consent must be confirmed by the personally dated signature of the subject and by the personally dated signature of the physician conducting the informed consent discussion.

If the subject is unable to write, oral presentation and explanation of the content of the informed consent form and of the data protection information must take place in the presence of an impartial witness. The witness and the physician conducting the informed consent discussions must also sign and personally date the consent document. The witness must not be in any way dependent on the sponsor of the trial, the trial site or any member of the investigating team (e. g. an employee at the trial site.).

A copy of the signed informed consent document must be given to the subject; the original will be filed by the investigator. The documents must be in a language understandable to the subject and must specify who informed the subject.

The subjects will be informed as soon as possible if new information may influence his/her decision to participate in the trial. The communication of this information should be documented.

11.6 Insurance

According to § 40 AMG, the sponsor has to subscribe to an insurance policy covering, in its terms and provisions, its legal liability for injuries caused to participating persons and arising out of this research performed strictly in accordance with the scientific protocol as well as with applicable law and professional standards. The insurance was taken out at HDI Global SE (insurance number: 57 010310 03018).

Any impairment of health which might occur in consequence of trial participation must be notified to the insurance company. The subject is responsible for notification. The insured person will agree with all appropriate measures serving for clarification of the cause and the extent of damage as well as the reduction of damage.

During the conduct of the trial, the subject must not undergo other clinical treatment except for cases of emergency. The subject is bound to inform the investigator immediately about any adverse events and additionally drugs taken. The terms and conditions of the insurance should be delivered to the subject.

The insurance company has to be informed about all amendments that could affect subjects' safety.

11.7 Continuous Information to the Ethics Committee and the Competent Authority

Pursuant to the German Drug Law (AMG) and the GCP Ordinance, the responsible EC, the competent authority and all participating investigators will be informed of all suspected unexpected serious adverse reactions (SUSARs) occurring during the trial. Both institutions will be informed in case the risk/ benefit assessment did change or any others new and significant hazards for subjects' safety or welfare did occur. Furthermore, a report on the subjects' safety will be submitted once a year – Development Safety Update Report (DSUR).

The EC and the regulatory authorities must be informed of the end of the trial. They will be provided with a summary of trial results within one year after the end of clinical phase (LSO).

12 QUALITY CONTROL AND QUALITY ASSURANCE

The sponsor, the investigators, and all involved study personnel agree to conduct this clinical trial in accordance with the ICH Guideline for Good Clinical Practice.

12.1 Direct Access to Source Documents According to ICH-GCP

According to ICH-GCP the investigator(s)/institution(s) must provide direct access to source data/documents for trial related monitoring, audits and regulatory inspection. Each subject has consented - via written informed consent - to direct access to his/her original medical records for trial-related monitoring, audit and regulatory inspection. Content of the protocol must be the identification of any data to be recorded directly on the CRFs (i.e., no prior written or electronic record of data), and to be considered to be source data (see 10.1).

In the absence of either an audit-trail or limited access for the monitor the electronic record of data must be printed out.

12.2 Data Protection

The data obtained in the course of the trial will be treated pursuant to the Federal Data Protection Law (Bundesdatenschutzgesetz, BDSG) and GDPR.

During the clinical trial, subjects will be identified solely by means of their individual identification code (subject number). Trial data stored on a computer will be stored in accordance with local data protection law and will be handled in strictest confidence. Distribution of these data to unauthorized persons has to be prevented strictly. The appropriate regulations of local data legislation will be fulfilled in its entirety.

The subject consents in writing to release the investigator from his/her professional discretion in so far as to allow inspection of original data for monitoring purposes by health authorities and authorized persons (inspectors, clinical monitors, auditors). Authorized persons (inspectors, clinical monitors, auditors) may inspect the subject-related data collected during the trial ensuring the data protection law.

The investigator will maintain a subject identification list (subject numbers with the corresponding subject names) to enable records to be identified. Subjects who did not consent to circulate their pseudonymized data will not be included into the trial.

This protocol, the eCRF and other trial-related documents and material must be handled with strict confidentiality and not be disclosed to third parties except with the express prior consent of Sponsor. In particular, it must be ensured that the study medication is kept out of reach of third parties. Staffs of the investigators involved in this study are also bound by this agreement.

12.3 Monitoring

Monitoring will be done by on-site and off-site visits and frequent communication (letters, telephone, fax, e-mail) by a clinical monitor according to SOPs of the KKS. The monitor will ensure that the trial is conducted according to the protocol and regulatory requirements by review of source documents, entries into the eCRFs and essential documents. Therefore, the investigator must allow the monitor to verify these documents (see also 12.1) and must provide support to the monitor at all times. The monitor will document the visits in a report for the sponsor. The trial site will be provided with a follow-up letter of the findings and the necessary actions to be taken.

As the monitoring strategy will consider current aspects of risk-based quality management, frequency of monitoring activities per site will vary depending on recruitment, experience, and general performance, e.g. quality of documentation of the individual trial sites. Details of monitoring will be defined in the monitoring manual.

If there are major findings during monitoring or an audit, the investigational site might be closed by the LKP.

12.4 Inspections and Audits

Regulatory authorities and/ or auditors authorised by the sponsor may request access to all source documents, eCRFs, and other trial documentation. Direct access to these documents must be guaranteed by the investigator who must provide support at all times for these activities.

The investigator will inform the sponsor immediately about a planned inspection.

12.5 Responsibilities of the Investigator

The investigator ensures that all team members are informed adequately about the protocol, all amendments to the protocol, the study procedures und study specific duties and tasks.

The investigator will maintain a list to delegate tasks to the team members.

13 ADMINISTRATIVE AGREEMENTS

13.1 Financing of the Trial

The trial will be financed using funds of Bristol-Myers Squibb.

13.2 Financial Disclosure

Before the start of the trial, the investigator will disclose to the sponsor any proprietary or financial interests he might hold in the funding company, in the investigational product(s) or any commercial organisation being involved in the clinical trial. The investigator has also to confirm that he has not entered into any financial arrangement, whereby the value of compensation paid could affect the outcome of the clinical trial. The investigator agrees to update this information in case of significant changes.

13.3 Reports

The biometrical / statistical report will be performed by Thomas Hielscher, Biostatistics, German Cancer Research Center, Heidelberg, Germany. Reports will be generated two months after closure of database.

Within one year of the completion of the trial, the competent federal authority and the ethics committee will be supplied with a summary of the final report on the clinical trial containing the principle results according to §42 AMG.

13.4 Registration of the Trial

Prior to the beginning of the clinical phase (FSI) the coordinating/ principal investigator/ sponsor will register the trial at Current Controlled Trials (http://www.controlled-trials.com/), http://www.clinicaltrials.gov or http://www.zks.uni-freiburg.de receiving a unique ISRCTN, which is a prerequisite for a publication in a peer-review paper. Alternatively, no further registration is required in case of a EUDRACT database listing, as this is accepted by the ICMJE.

13.5 Publication

All information concerning the trial is confidential before publication. Publication(s) and/or presentation(s) of the study results is encouraged after appropriate time for review and written agreement by the sponsor. The sponsor has to be provided with a draft of the abstract and/or manuscript for review and editorial comments at least 30 days prior to submission and/or presentation. Neither the sponsor nor the Coordinating Investigator has the right to prevent publication, except for patent or copyright purposes.

Study data published or disclosed to third parties must not contain data that allow the identification of a subject.

KKS staff members who gave relevant scientific support to the study design, conductance and/or analysis of results will be included as coauthors, if applicable. A copy of all publications will be sent to the KKS.

15 REFERENCES

Abbruzzese JL, Abbruzzese MC, Lenzi R, et al. Analysis of a diagnostic strategy for subjects with suspected tumors of unknown origin. J Clin Oncol 1995; 13: 2094-2103.

Abrams HL, Spiro R, Goldstein N. Metastases in carcinoma; analysis of 1000 autopsied cases. Cancer 1950; 3: 74-85.

Anderson K. gsDesign: Group Sequential Design. R package version 3.0-1. https://CRAN.R-project.org/package=gsDesign, 2016.

Antonia SJ, López-Martin JA, Bendell J, et al. Nivolumab alone and nivolumab plus ipilimumab in recurrent small-cell lung cancer (CheckMate 032): a multicentre, open-label, phase 1/2 trial. Lancet Oncol 2016; 17: 883-895.

Buchhalter I, Rempel E, Endris V, et al. Size matters: Dissecting key parameters for panel-based tumor mutational burden. Int J Cancer 2018 (epub ahead of print).

Busson P, Daya-Grosjean L, Pavlidis N, et al. The biology of unknown primary tumors: the little we know, the importance of learning more. In: Carcinoma of an unknown primary site. Fizazi K (editor) 2006, New York, Taylor & Francis.

CHECKMATE 817 A Phase IIIb/IV Safety Trial of Flat Dose Nivolumab in Combination with Ipilimumab in Participants with Non-Small Cell Lung Cancer, WCLC 2018

Culine S, Kramar A, Saghatchian M, et al. Development and validation of a prognostic model to predict the length of survival in patients with carcinomas of an unknown primary site. J Clin Oncol 2002; 20: 4679-4683.

Culine S, Ychou M, Fabbro M,, et al. 5-fluorouracil and leukovorin as second-line chemotherapy in carcinomas of unknown primary site. Anticancer Res 2001; 21: 1455-1457.

Didolkar MS, Fanous N, Elias EG, et al. Metastatic carcinomas from occult primary tumors. A study of 254 patients. Ann Surg 1977; 186: 625-630.

Fizazi K, Greco FA, Pavlidis N, et al. Cancers of unknown primary site: ESMO Clinical Practice Guidelines for diagnosis, treatment and follow-up. Ann Oncol 2015; 26 Suppl 5: v133-138.

Forde PM, Chaft JE, Smith KN, et al. Neoadjuvant PD-1 blockade in resectable lung cancer. N Engl J Med 2018; 378: 1976-1986.

Gay LM, Fabrizio D, Frampton GM, et al. Mutational burden of tumors with primary site unknown. J Clin Oncol 2017; 35: 3039.

Golfinopoulos V, Pentheroudakis G, Salanti G, et al. Comparative survival with diverse chemotherapy regimens for cancer of unknown primary site: multiple-treatments meta-analysis. Cancer Treat Rev 2009; 35: 570-573.

Gröschel S, Bommer M, Hutter B, et al. Integration of genomics and histology revises diagnosis and enables effective therapy of refractory cancer of unknown primary with PDL1 amplification. Cold Spring Harb Mol Case Stud 2016; 2: a001180.

Hainsworth JD, Burris HA, Calvert SM, et al. Gemcitabine in the second-line therapy of patients with carcinoma of unknown primary site: A phase II trial of the Minnie Pearl Cancer Res Network. Cancer Invest 2001; 19: 335-339.

Hainsworth JD, Spigel DR, Raefsky EL, et al. Combination chemotherapy with gemcitabine and irinotecan in patients with previously treated carcinoma of unknown primary site: A Minnie Pearl Cancer Res Network phase II trial. Cancer 2005; 104: 1992-1997.

Hainsworth JD, Fizazi K. Treatment for patients with unknown primary cancer and favorable prognostic factors. Semin Oncol 2009; 36: 44-51.

Hellmann MD, Rizvi NA, Goldman JW, et al. Nivolumab plus ipilimumab as first-line treatment for advanced non-small-cell lung cancer (CheckMate012): results of an open-label, phase 1, multicohort study. Lancet Oncol 2017; 18: 31-41.

Hellmann MD, Ciuleanu TE, Pluzanski A, et al. Nivolumab plus ipilimumab in lung cancer with a high tumor mutational burden. N Engl J Med 2018; 378: 2093-2104.

Jemal A, Siegel R, Ward E, et al. Cancer statistics, 2008. CA Cancer J Clin 2008; 58: 71-96.

Jordan WE, Shildt RA. Adenocarcinoma of unknown primary site. The Brooke Army Medical Center experience. Cancer 1985; 55: 857-860.

Krämer A, Losa F, Gay LM, et al. Genomic profiling of carcinomas of unknown primary (CUP) to support clinical decisions. J Clin Oncol 2018; e24162.

Lachin JM, Foulkes MA. Evaluation of sample size and power for analyses of survival with allowance for nonuniform patient entry, losses to follow-up, noncompliance, and stratification. Biometrics 1986; 42: 507-519.

Lachin JM. A review of methods for futility stopping based on conditional power. Stat Med 2005; 24: 2747-2764.

Le DT, Durham JN, Smith KN, et al. Mismatch repair deficiency predicts response of solid tumors to PD-1 blockade. Science 2017; 357: 409-413.

Le Chevalier T, Cvitkovic E, Caille P, et al. Early metastatic cancer of unknown primary origin at presentation. A clinical study of 302 consecutive autopsied subjects. Arch Intern Med 1988; 148: 2035-2039.

Marshall HT, Djamgoz MBA. Immuno-oncology: emerging targets and combination therapies. Front Oncol 2018; 8: 315.

Massard C, Loriot Y, Fizazi K. Carcinomas of an unknown primary origin - diagnosis and treatment. Nat Rev Clin Oncol 2011; 8: 701-710.

Mayordomo JI, Guerra JM, Guijarro C, et al. Neoplasms of unknown primary site: a clinicopathological study of autopsied patients. Tumori 1993; 79: 321-324.

Motzer RJ, Tannir NM, McDermott DF, et al. Nivolumab plus ipilimumab versus sunitinib in advanced renal-cell carcinoma. N Engl J Med 2018; 378: 1288-1290.

Möller AK, Pedersen KD, Abildgaard J, et al. Capecitabine and oxaliplatin as second-line treatment in patients with carcinoma of unknown primary site. Acta Oncol 2010; 49: 431-435.

Oien KA. Pathologic evaluation of unknown primary cancer. Semin Oncol 2009; 36: 8-37.

Overman MJ, McDermott R, Leach JL, et al. Nivolumab in patients with metastatic DNA mismatch repair-deficient or microsatellite instability-high colorectal cancer (CheckMate 142): an open-label, multicentre, phase 2 study. Lancet Oncol 2017; 18:1182-1191.

Pavlidis N, Fizazi K. Carcinoma of unknown primary (CUP). Crit Rev Oncol Hematol 2009; 69: 271-278.

Pavlidis N, Pentheroudakis G, Plataniotis G, et al. Cervical lymph node metastases of squamous cell carcinoma from an unknown primary site: a favourable prognosis subset of patients with CUP. Clin Transl Oncol 2009; 11: 340-348.

Pentheroudakis G, Golfinopoulos V, Pavlidis N. Switching benchmarks in cancer of unknown primary: from autopsy to microarray. Eur J Cancer 2007; 43: 2026-2036.

Pentheroudakis G, Lazaridis G, Pavlidis N. Axillary nodal metastases from carcinoma of unknown primary (CUPAx): a systematic review of published evidence. Breast Cancer Res Treat 2010; 119: 1-11.

Pentheroudakis G, Pavlidis N. Serous papillary peritoneal carcinoma: unknown primary tumour, ovarian cancer counterpart or a distinct entity? A systematic review. Crit Rev Oncol Hematol 2010; 75: 27-42.

Postow MA, Sidlow R, Hellmann MD. Immue-related adverse events associated with immune checkpoint blockade. N Engl J Med 2018; 378: 158-168.

Rizvi NA, Hellmann MD, Snyder A, et al. Cancer immunology. Mutational landscape determines sensitivity to PD-1 blockade in non-small cell lung cancer. Science 2015; 348: 124-128.

Samstein RM, Lee CH, Shoushtari AN, et al. Tumor mutational load predicts survival after immunotherapy across multiple tumor types. Nat Genet 2019; doi: 10.1038/s41588-018-0312-8. [Epub ahead of print].

Spigel DR, Hainsworth JD, Greco FA. Neuroendocrine carcinoma of unknown primary site. Semin Oncol 2009; 36: 52-59.

Varghese AM, Arora A, Capanu-M, et al. Clinical and molecular characterization of patients with cancer of unknown primary in the modern era. Ann Oncol 2017; 28: 3015-3021.

Wolchok JD, Chiarion-Sileni V, Gonzalez R, et al. Overall survival with combined nivolumab and ipilimumab in advanced melanoma. N Engl J Med 2017; 377: 1345-1356.

16 APPENDICES

Appendix 1

Highly-effective contraceptive methods

Highly-effective contraceptive methods according to the recommendations by the Clinical Trial Facilitation Group (CTFG) (this may also apply to the trial subject's partner depending on the investigational product (IMP)).

The following contraceptive methods with a Pearl Index lower than 1 are regarded as highly-effective:

- Combined (estrogen and progestogen containing) hormonal contraception associated with the inhibition of ovulation:
  - oral
  - intravaginal
  - transdermal

  as far as the efficacy is not expected to be impaired during the trial, e.g. with IMPs that cause vomiting and diarrhea or interfere with hormone metabolism, adequate safety cannot be assumed.
- Progestogen-only hormonal contraception associated with the inhibition of ovulation:
  - oral
  - injectable
  - implantable

  as far as the efficacy is not expected to be impaired during the trial, e.g. with IMPs that cause vomiting and diarrhea or interfere with hormone metabolism, adequate safety cannot be assumed.
- Tubal ligation (female sterilisation)
- Vasectomized partner provided that the partner is the sole sexual partner and the procedure was medically assessed as surgical success
- Intrauterine device
- Intrauterine devices that release hormones (hormone spiral)
- Sexual abstinence if consisting with the preferred and usual lifestyle of the subject

This means that the following are not regarded as safe: periodic abstinence (calendar, symptothermal, post-ovulation methods), condom plus spermicide, spermicides only, simple barrier methods (vaginal pessaries, condom, female condoms), the lactational amenorrhea method or the withdrawal method (coitus interruptus). Female condom and male condom should not be used together.

The obligation to ensure effective contraception is based on Guideline ICH E8 Chapter 3.2.2.1 Selection of subjects together with ICH M3 Note 4.

Appendix 2
Response Evaluation Criteria in Solid Tumors, Version 1.1: Excerpt from Original Publication

Selected sections from the Response Evaluation Criteria in Solid Tumors (RECIST), Version 1.1 [1] are presented below, with slight modifications and the addition of explanatory text as needed for clarity.[2]

MEASURABILITY OF TUMOR AT BASELINE

DEFINITIONS

At baseline, tumor lesions/lymph nodes will be categorized measurable or non-measurable as follows:

Measurable Tumor Lesions

Tumor Lesions. Tumor lesions must be accurately measured in at least one dimension (longest diameter in the plane of measurement is to be recorded) with a minimum size of:

- 10 mm by computed tomography (CT) or magnetic resonance imaging (MRI) scan (CT/MRI scan slice thickness/interval no greater than 5 mm)
- 10-mm caliper measurement by clinical examination (lesions that cannot be accurately measured with calipers should be recorded as nonmeasurable)
- 20 mm by chest X-ray

Malignant Lymph Nodes. To be considered pathologically enlarged and measurable, a lymph node must be $\geq$15 mm in the short axis when assessed by CT scan (CT scan slice thickness recommended to be no greater than 5 mm). At baseline and in follow-up, only the short axis will be measured and followed. See also notes below on “Baseline Documentation of Target and Nontarget Lesions” for information on lymph node measurement.

Non-measurable Tumor Lesions

Non-measurable tumor lesions encompass small lesions (longest diameter $<$10 mm or pathological lymph nodes with $\geq$10 to $<$15 mm short axis), as well as truly non-measurable lesions. Lesions considered truly non-measurable include leptomeningeal disease, ascites, pleural or pericardial effusion, inflammatory breast disease, lymphangitic involvement of skin or lung, peritoneal spread, and abdominal masses/abdominal organomegaly identified by physical examination that is not measurable by reproducible imaging techniques.

Special Considerations Regarding Lesion Measurability

Bone lesions, cystic lesions, and lesions previously treated with local therapy require particular comment, as outlined below.

---

1 Eisenhauer EA, Therasse P, Bogaerts J, et al. New response evaluation criteria in solid tumors: Revised RECIST guideline (Version 1.1). Eur J Cancer 2009;45:228–47.

2 For consistency within this document, the section numbers and cross-references to other sections within the article have been deleted and minor formatting changes have been made.

Bone lesions:

- Bone scan, positron emission tomography (PET) scan, or plain films are not considered adequate imaging techniques to measure bone lesions. However, these techniques can be used to confirm the presence or disappearance of bone lesions.
- Lytic bone lesions or mixed lytic-blastic lesions, with identifiable soft tissue components, that can be evaluated by cross-sectional imaging techniques such as CT or MRI can be considered measurable lesions if the soft tissue component meets the definition of measurability described above.
- Blastic bone lesions are non-measurable.

Cystic lesions:

- Lesions that meet the criteria for radiographically defined simple cysts should not be considered malignant lesions (neither measurable nor non-measurable) since they are, by definition, simple cysts.
- Cystic lesions thought to represent cystic metastases can be considered measurable lesions if they meet the definition of measurability described above. However, if non-cystic lesions are present in the same patient, these are preferred for selection as target lesions.

Lesions with prior local treatment:

- Tumor lesions situated in a previously irradiated area, or in an area subjected to other loco-regional therapy, are usually not considered measurable unless there has been demonstrated progression in the lesion. Study protocols should detail the conditions under which such lesions would be considered measurable.

TARGET LESIONS: SPECIFICATIONS BY METHODS OF MEASUREMENTS

Measurement of Lesions

All measurements should be recorded in metric notation, with use of calipers if clinically assessed. All baseline evaluations should be performed as close as possible to the treatment start and never more than 4 weeks before the beginning of the treatment.

Method of Assessment

The same method of assessment and the same technique should be used to characterize each identified and reported lesion at baseline and during study. Imaging-based evaluation should always be the preferred option.

Clinical Lesions. Clinical lesions will only be considered measurable when they are superficial and $\geq$10 mm in diameter as assessed using calipers (e.g., skin nodules). For the case of skin lesions, documentation by color photography, including a ruler to estimate the size of the lesion, is suggested.

Chest X-Ray. Chest CT is preferred over chest X-ray, particularly when progression is an important endpoint, since CT is more sensitive than X-ray, particularly in identifying new lesions. However, lesions on chest X-ray may be considered measurable if they are clearly defined and surrounded by aerated lung.

CT, MRI. CT is the best currently available and reproducible method to measure lesions selected for response assessment. This guideline has defined measurability of lesions on CT scan based on the assumption that CT slice thickness is 5 mm or less. When CT scans have slice thickness greater than 5 mm, the minimum size for a measurable lesion should be twice the slice thickness. MRI is also acceptable.

If prior to enrollment it is known that a patient is unable to undergo CT scans with intravenous (IV) contrast because of allergy or renal insufficiency, the decision as to whether a non-contrast CT or MRI (without IV contrast) will be used to evaluate the patient at baseline and during the study should be guided by the tumor type under investigation and the anatomic location of the disease. For patients who develop contraindications to contrast after baseline contrast CT is done, the decision as to whether non-contrast CT or MRI (enhanced or nonenhanced) will be performed should also be based on the tumor type and the anatomic location of the disease and should be optimized to allow for comparison with the prior studies if possible. Each case should be discussed with the radiologist to determine if substitution of these other approaches is possible and, if not, the patient should be considered not evaluable from that point forward. Care must be taken in measurement of target lesions on a different modality and interpretation of non-target disease or new lesions since the same lesion may appear to have a different size with use of a new modality.

Ultrasound. Ultrasound is not useful in the assessment of lesion size and should not be used as a method of measurement.

Endoscopy, Laparoscopy, Tumor Markers, Cytology, Histology. The utilization of these techniques for objective tumor evaluation cannot generally be advised.

TUMOR RESPONSE EVALUATION

ASSESSMENT OF OVERALL TUMOR BURDEN AND MEASURABLE DISEASE

To assess objective response or future progression, it is necessary to estimate the overall tumor burden at baseline and to use this as a comparator for subsequent measurements. Measurable disease is defined by the presence of at least one measurable lesion, as detailed above.

BASELINE DOCUMENTATION OF TARGET AND NONTARGET LESIONS

When more than one measurable lesion is present at baseline, all lesions up to a maximum of five lesions total (and a maximum of two lesions per organ) representative of all involved organs should be identified as target lesions and will be recorded and measured at baseline. This means in instances where patients have only one or two organ sites involved, a maximum of two lesions (one site) and four lesions (two sites), respectively, will be recorded. Other lesions (albeit measurable) in those organs will be recorded as nonmeasurable lesions (even if the size is $>$10 mm by CT scan).

Target lesions should be selected on the basis of their size (lesions with the longest diameter) and be representative of all involved organs, but additionally, should lend themselves to reproducible repeated measurements. It may be the case that, on occasion, the largest lesion

does not lend itself to reproducible measurement, in which circumstance the next largest lesion that can be measured reproducibly should be selected.

Lymph nodes merit special mention since they are normal anatomical structures that may be visible by imaging even if not involved by tumor. As noted above, pathological nodes that are defined as measurable and may be identified as target lesions must meet the criterion of a short axis of $\geq$15 mm by CT scan. Only the short axis of these nodes will contribute to the baseline sum. The short axis of the node is the diameter normally used by radiologists to judge if a node is involved by solid tumor. Nodal size is normally reported as two dimensions in the plane in which the image is obtained (for CT scan, this is almost always the axial plane; for MRI the plane of acquisition may be axial, sagittal, or coronal). The smaller of these measures is the short axis. For example, an abdominal node that is reported as being 20 mm $\times$ 30 mm has a short axis of 20 mm and qualifies as a malignant, measurable node. In this example, 20 mm should be recorded as the node measurement. All other pathological nodes (those with short axis $\geq$10 mm but $<$15 mm) should be considered nontarget lesions. Nodes that have a short axis $<$10 mm are considered nonpathological and should not be recorded or followed.

A sum of the diameters (longest for non-nodal lesions, short axis for nodal lesions) for all target lesions will be calculated and reported as the baseline sum of diameters. If lymph nodes are to be included in the sum, then, as noted above, only the short axis is added into the sum. The baseline sum of diameters will be used as a reference to further characterize any objective tumor regression in the measurable dimension of the disease.

<u>All other lesions (or sites of disease), including pathological lymph nodes, should be identified as nontarget lesions and should also be recorded at baseline. Measurements are not required and these lesions should be followed as “present,” “absent,” or in rare cases “unequivocal progression.”</u>

In addition, it is possible to record multiple nontarget lesions involving the same organ as a single item on the Case Report Form (CRF) (e.g., “multiple enlarged pelvic lymph nodes” or “multiple liver metastases”).

RESPONSE CRITERIA

<u>Evaluation of Target Lesions</u>

This section provides the definitions of the criteria used to determine objective tumor response for target lesions.

- **Complete response (CR)**: disappearance of all target lesions

  Any pathological lymph nodes (whether target or nontarget) must have reduction in short axis to $<$10 mm.
- **Partial response (PR)**: at least a 30% decrease in the sum of diameters of target lesions, taking as reference the baseline sum of diameters
- **Progressive disease (PD)**: at least a 20% increase in the sum of diameters of target lesions, taking as reference the smallest sum on study (nadir), including baseline

In addition to the relative increase of 20%, the sum must also demonstrate an absolute increase of at least 5 mm.

The appearance of one or more new lesions is also considered progression.

- **Stable disease (SD)**: neither sufficient shrinkage to qualify for PR nor sufficient increase to qualify for PD, taking as reference the smallest sum on study

Special Notes on the Assessment of Target Lesions

Lymph Nodes. Lymph nodes identified as target lesions should always have the actual short axis measurement recorded (measured in the same anatomical plane as the baseline examination), even if the nodes regress to < 10 mm on study. This means that when lymph nodes are included as target lesions, the sum of lesions may not be zero even if CR criteria are met since a normal lymph node is defined as having a short axis < 10 mm.

Target Lesions That Become Too Small to Measure. While on study, all lesions (nodal and non-nodal) recorded at baseline should have their actual measurements recorded at each subsequent evaluation, even when very small (e.g., 2 mm). However, sometimes lesions or lymph nodes that are recorded as target lesions at baseline become so faint on the CT scan that the radiologist may not feel comfortable assigning an exact measure and may report them as being too small to measure. When this occurs, it is important that a value be recorded on the eCRF as follows:

- If it is the opinion of the radiologist that the lesion has likely disappeared, the measurement should be recorded as 0 mm.
- If the lesion is believed to be present and is faintly seen but too small to measure, a default value of 5 mm should be assigned and below measurable limit (BML) should be ticked. (Note: It is less likely that this rule will be used for lymph nodes since they usually have a definable size when normal and are frequently surrounded by fat such as in the retroperitoneum; however, if a lymph node is believed to be present and is faintly seen but too small to measure, a default value of 5 mm should be assigned in this circumstance as well and BML should also be ticked.)

<u>To reiterate, however, if the radiologist is able to provide an actual measure, that should be recorded, even if it is below 5 mm, and, in that case, BML should not be ticked.</u>

Lesions That Split or Coalesce on Treatment. When non-nodal lesions fragment, the longest diameters of the fragmented portions should be added together to calculate the target lesion sum. Similarly, as lesions coalesce, a plane between them may be maintained that would aid in obtaining maximal diameter measurements of each individual lesion. If the lesions have truly coalesced such that they are no longer separable, the vector of the longest diameter in this instance should be the maximal longest diameter for the coalesced lesion.

Evaluation of Nontarget Lesions

This section provides the definitions of the criteria used to determine the tumor response for the group of nontarget lesions. Whereas some non-target lesions may actually be measurable,

they need not be measured and, instead, should be assessed only qualitatively at the time points specified in the protocol.

- **CR**: disappearance of all non-target lesions and (if applicable) normalization of tumor marker level)

  All lymph nodes must be non-pathological in size (< 10 mm short axis).

- **Non-CR/Non-PD**: persistence of one or more non-target lesion(s) and/or (if applicable) maintenance of tumor marker level above the normal limits
- **PD**: unequivocal progression of existing nontarget lesions

  The appearance of one or more new lesions is also considered progression.

Special Notes on Assessment of Progression of Nontarget Disease

When the Patient Also Has Measurable Disease. In this setting, to achieve unequivocal progression on the basis of the nontarget disease, there must be an overall level of substantial worsening in non-target disease in a magnitude that, even in the presence of SD or PR in target disease, the overall tumor burden has increased sufficiently to merit discontinuation of therapy. A modest increase in the size of one or more non-target lesions is usually not sufficient to qualify for unequivocal progression status. The designation of overall progression solely on the basis of change in non-target disease in the face of SD or PR of target disease will therefore be extremely rare.

When the Patient Has Only Non-Measurable Disease. This circumstance arises in some Phase III trials when it is not a criterion of study entry to have measurable disease. The same general concepts apply here as noted above; however, in this instance, there is no measurable disease assessment to factor into the interpretation of an increase in non-measurable disease burden. Because worsening in non-target disease cannot be easily quantified (by definition: if all lesions are truly non-measurable), a useful test that can be applied when assessing patients for unequivocal progression is to consider if the increase in overall disease burden based on the change in non-measurable disease is comparable in magnitude to the increase that would be required to declare PD for measurable disease, that is, an increase in tumor burden representing an additional 73% increase in volume (which is equivalent to a 20% increase in diameter in a measurable lesion). Examples include an increase in a pleural effusion from “trace” to “large” or an increase in lymphangitic disease from localized to widespread or may be described in protocols as “sufficient to require a change in therapy.” If unequivocal progression is seen, the patient should be considered to have had overall PD at that point. Whereas it would be ideal to have objective criteria to apply to non-measurable disease, the very nature of that disease makes it impossible to do so; therefore, the increase must be substantial.

When the patient has bone lesions at baseline. When a bone scan is the sole indicator of progression, progression in bone will be defined as when at least two or more new lesions are seen on bone scan compared with screening. In situations where the scan findings are suggestive of a flare reaction, or apparent new lesion(s) which may represent trauma, these results must be confirmed with other imaging modalities such as MRI or fine-cut CT to constitute

progression. Only a single new bone lesion on bone scan is required for progression if the lesion can be correlated on CT, MRI or plain film.

New Lesions

The appearance of new malignant lesions denotes disease progression; therefore, some comments on detection of new lesions are important. There are no specific criteria for the identification of new radiographic lesions; however, the finding of a new lesion should be unequivocal, that is, not attributable to differences in scanning technique, change in imaging modality, or findings thought to represent something other than tumor (for example, some "new" bone lesions may be simply healing or flare of preexisting lesions). This is particularly important when the patient's baseline lesions show partial or complete response. For example, necrosis of a liver lesion may be reported on a CT scan report as a "new" cystic lesion, which it is not.

A lesion identified during the study in an anatomical location that was not scanned at baseline is considered a new lesion and will indicate disease progression.

If a new lesion is equivocal, for example because of its small size, continued therapy and follow-up evaluation will clarify if it represents truly new disease. If repeat scans confirm there is definitely a new lesion, then progression should be declared using the date of the initial scan.

New osteoblastic bone lesions identified on plain films, CT, or MRI will not be considered progression in an otherwise stable or responding subject, if, in the opinion of the physician, the osteoblastic lesion appears to be healing or a response to therapy.

EVALUATION OF RESPONSE

Timepoint Response (Overall Response)

It is assumed that at each protocol-specified time point, a response assessment occurs. Appendix 2 Table 1 provides a summary of the overall response status calculation at each time point for patients who have measurable disease at baseline.

When patients have non-measurable (therefore non-target) disease only, Appendix 2 Table 2 is to be used.

Appendix 2 Table 1 Timepoint Response: Patients with Target Lesions (with or without Non-target Lesions)

Target Lesions	Nontarget Lesions	New Lesions	Overall Response
CR	CR	No	CR
CR	Non-CR/non-PD	No	PR
CR	Not evaluated	No	PR
PR	Non-PD or not all evaluated	No	PR
SD	Non-PD or not all evaluated	No	SD
Not all evaluated	Non-PD	No	NE
PD	Any	Yes or no	PD
Any	PD	Yes or no	PD
Any	Any	Yes	PD

CR = complete response; NE = not evaluable; PD = progressive disease; PR = partial response; SD = stable disease.

Appendix 2 Table 2 Timepoint Response: Patients with Non-target Lesions Only

Non-target Lesions	New Lesions	Overall Response
CR	No	CR
Non-CR/non-PD	No	Non-CR/non-PD [a]
Not all evaluated	No	NE
Unequivocal PD	Yes or no	PD
Any	Yes	PD

CR = complete response; NE = not evaluable; PD = progressive disease.

[a] "Non-CR/non-PD" is preferred over "stable disease" for non-target disease since stable disease is increasingly used as an endpoint for assessment of efficacy in some trials; thus, assigning "stable disease" when no lesions can be measured is not advised.

Missing Assessments and Not-Evaluable Designation

When no imaging/measurement is done at all at a particular time point, the patient is not evaluable at that time point. If only a subset of lesion measurements is made at an assessment, usually the case is also considered not evaluable at that time point, unless a convincing argument can be made that the contribution of the individual missing lesion(s) would not change the assigned time point response. This would be most likely to happen in the case of PD. For example, if a patient had a baseline sum of 50 mm with three measured lesions and, during the study, only two lesions were assessed, but those gave a sum of 80 mm, the patient will have achieved PD status, regardless of the contribution of the missing lesion.

If one or more target lesions were not assessed either because the scan was not done or the scan could not be assessed because of poor image quality or obstructed view, the response for target lesions should be "unable to assess" since the patient is not evaluable. Similarly, if one or more non-target lesions are not assessed, the response for non-target lesions should be

"unable to assess" except where there is clear progression. Overall response would be "unable to assess" if either the target response or the non-target response is "unable to assess," except where this is clear evidence of progression as this equates with the case being not evaluable at that time point.

Appendix 2 Table 3 Best Overall Response When Confirmation Is Required

Overall Response at First Timepoint	Overall Response at Subsequent Timepoint	Best Overall Response
CR	CR	CR
CR	PR	SD, PD, or PR [a]
CR	SD	SD, provided minimum duration for SD was met; otherwise, PD
CR	PD	SD, provided minimum duration for SD was met; otherwise, PD
CR	NE	SD, provided minimum duration for SD was met; otherwise, NE
PR	CR	PR
PR	PR	PR
PR	SD	SD
PR	PD	SD, provided minimum duration for SD was met; otherwise, PD
PR	NE	SD, provided minimum duration for SD was met; otherwise, NE
NE	NE	NE

CR = complete response; NE = not evaluable; PD = progressive disease; PR = partial response; SD = stable disease.

[a] If a CR is truly met at the first time point, any disease seen at a subsequent time point, even disease meeting PR criteria relative to baseline, qualifies as PD at that point (since disease must have reappeared after CR). Best response would depend on whether the minimum duration for SD was met. However, sometimes CR may be claimed when subsequent scans suggest small lesions were likely still present and in fact the patient had PR, not CR, at the first time point. Under these circumstances, the original CR should be changed to PR and the best response is PR.

NOTE: In this study, stable disease must persist for at least 6 weeks (minimum duration) to be considered a bona fide SD.

Special Notes on Response Assessment

When nodal disease is included in the sum of target lesions and the nodes decrease to "normal" size (< 10 mm), they may still have a measurement reported on scans. This measurement should be recorded even though the nodes are normal in order not to overstate progression should it be based on increase in size of the nodes. As noted earlier, this means that patients with CR may not have a total sum of "zero" on the CRF.

Patients with a global deterioration of health status requiring discontinuation of treatment without objective evidence of disease progression at that time should be reported as

"symptomatic deterioration." Every effort should be made to document objective progression even after discontinuation of treatment. Symptomatic deterioration is not a descriptor of an objective response; it is a reason for stopping study therapy. The objective response status of such patients is to be determined by evaluation of target and nontarget disease as shown in Appendix 2 Table 1, Appendix 2 Table 2, and Appendix 2 Table 3.

For equivocal findings of progression (e.g., very small and uncertain new lesions; cystic changes or necrosis in existing lesions), treatment may continue until the next scheduled assessment. If at the next scheduled assessment progression is confirmed, the date of progression should be the earlier date when progression was suspected.

In studies for which patients with advanced disease are eligible (i.e., primary disease still or partially present), the primary tumor should also be captured as a target or non-target lesion, as appropriate. This is to avoid an incorrect assessment of complete response if the primary tumor is still present but not evaluated as a target or non-target lesion.
