# Supplementary material for: Nivolumab and ipilimumab in recurrent or refractory cancer of unknown primary: a phase II trial
Source: Nat Commun. 2023 Oct 24;14:6761. doi: 10.1038/s41467-023-42400-5 (PMC10598029; doi:10.1038/s41467-023-42400-5)
Supplement: Supplementary file 3 — Description of Additional Supplementary Files [file 41467_2023_42400_MOESM3_ESM.pdf]

Description of Additional Supplementary Files

Supplementary Data 1. Potentially clinically relevant SNVs/indels and CNAs detected in FFPE tissue samples from the CUP cohort.

Supplementary Data 2. Genomic regions targeted by the CheCUP panel (hg19).

Supplementary Data 3. List of CheCUP panel probes.

Supplementary Data 4: Normalized Log Data of the gene expression analysis using the NanoString nCounter technology
