# Supplementary material for: Nivolumab and ipilimumab in recurrent or refractory cancer of unknown primary: a phase II trial
Source: Nat Commun. 2023 Oct 24;14:6761. doi: 10.1038/s41467-023-42400-5 (PMC10598029; doi:10.1038/s41467-023-42400-5)
Supplement: Supplementary file 8 — Reporting Summary [file 41467_2023_42400_MOESM8_ESM.pdf]

nature portfolio

Corresponding author(s): Alwin Krämer

Last updated by author(s): Sep 25, 2023

Reporting Summary

Nature Portfolio wishes to improve the reproducibility of the work that we publish. This form provides structure for consistency and transparency in reporting. For further information on Nature Portfolio policies, see our Editorial Policies and the Editorial Policy Checklist.

Statistics

For all statistical analyses, confirm that the following items are present in the figure legend, table legend, main text, or Methods section.

n/a	Confirmed	
☐	☒	The exact sample size (*n*) for each experimental group/condition, given as a discrete number and unit of measurement
☐	☒	A statement on whether measurements were taken from distinct samples or whether the same sample was measured repeatedly
☐	☒	The statistical test(s) used AND whether they are one- or two-sided<br>*Only common tests should be described solely by name; describe more complex techniques in the Methods section.*
☐	☒	A description of all covariates tested
☐	☒	A description of any assumptions or corrections, such as tests of normality and adjustment for multiple comparisons
☐	☒	A full description of the statistical parameters including central tendency (e.g. means) or other basic estimates (e.g. regression coefficient) AND variation (e.g. standard deviation) or associated estimates of uncertainty (e.g. confidence intervals)
☐	☒	For null hypothesis testing, the test statistic (e.g. *F*, *t*, *r*) with confidence intervals, effect sizes, degrees of freedom and *P* value noted<br>*Give P values as exact values whenever suitable.*
☐	☒	For Bayesian analysis, information on the choice of priors and Markov chain Monte Carlo settings
☒	☐	For hierarchical and complex designs, identification of the appropriate level for tests and full reporting of outcomes
☐	☒	Estimates of effect sizes (e.g. Cohen's *d*, Pearson's *r*), indicating how they were calculated

Our web collection on statistics for biologists contains articles on many of the points above.

Software and code

Policy information about availability of computer code

Data collection	In this trial a clinical data management system provided by the KKS was used for data collection using an electronic CRF (eCRF) for remote data entry (RDE).<br>All entries in the eCRF had to be verifiable by source documents. A detailed list of entries to eCRF was provided in the Investigator Site File. Regardless, there had to be a minimum documentation, which provided information on study participation and included all medical information necessary for appropriate medical care outside of the clinical trial in the patient record.<br>In addition, source documents had to mention that the subject had been included in an investigational study. Finally, there had to be no data that were inconsistent between eCRF and source documents.<br>All protocol-required information collected during the trial had to be entered by the investigator or a designated representative into the eCRF. Patient data was documented pseudonymously. The investigator or a designated representative should complete the eCRF pages as soon as possible after the information was collected, preferably within 2 weeks after the study visit. Any pending entries had to be completed immediately after the final examination. Explanation had to be given for all missing data.<br>The investigator was responsible for ensuring that all sections of the eCRF were completed correctly. Any errors had to be corrected in the eCRF and a reason for change had to be entered. The correctness of all entries in eCRF had to be confirmed by dated electronic signature of the responsible investigator. The time points and frequency of electronic signatures was defined in the study specific document 'eCRF specification.
Data analysis	Statistical analyses and data visualization were performed using R version 4.1.0 (www.r-project.org) or GraphPad Prism version 7.03 (GraphPad Software), and a two-sided p-value <0.05 was considered to be statistically significant. All patients enrolled into the trial had valid TMB assessment and received trial medication, i.e., there is only one analysis population for efficacy and safety. The Kaplan-Meier method was used to estimate PFS and OS. Prognostic impact of genomic and clinical baseline parameters on PFS and OS was assessed using log-rank test and Cox regression models. Secondary analyses of the primary endpoint comprised a multivariable Cox regression model including

relevant prognostic factors. Fisher's exact test was used to compare response rates. Correlation analyses were performed based on Spearman's correlation coefficient. Group and sample comparisons were made using either two-tailed Mann-Whitney U test or Wilcoxon matched-pairs signed rank test. Incidence and severity of adverse events were analyzed for the safety population. Due to the premature discontinuation of the trial, all analyses were exploratory. Copy-number variation analysis from the methylation array data was performed as described by Capper et al using the conumee Bioconductor package v.1.34.0 in R v.4.3.1. The CheCUP panel design for targeted ccfDNA sequencing was designed using the xGen Hyb Panel Design Tool (Integrated DNA Technologies, Inc) using genome build hg19 NCBI Build 37.1/ GRCh37 as reference. For the sequencing analysi of ccfDNA Raw sequencing reads were processed and aligned using the Subread aligner. PCR duplicates and process artifacts were detected and removed by molecular barcoding using the UMI-tools. Genome-wide copy number profiles and tumour fractions (TFx) were estimated from sWGS data using the ichorCNA algorithm (https://github.com/broadinstitute/ichorCNA) in R (version 4.1.0). First, HMMcopy Suite (http://compbio.bccrc.ca/software/hmmcopy/) was used to partition the genome into equally sized bins of 1Mb. The read counts were corrected for GC content and mappability biases using the HMMcopy R package. A Bayesian statistical framework of the hidden Markov model (HMM) and an expectation-maximization (EM) algorithm were used to predict CNAs and estimate TFx. A reference panel of normal samples was generated from the sWGS data of the 19 healthy subjects for CNA analysis. The aneuploidy score (AS) was calculated from the genome-wide copy number profiles as the sum total of altered chromosome arms, as described by Taylor et al.

For manuscripts utilizing custom algorithms or software that are central to the research but not yet described in published literature, software must be made available to editors and reviewers. We strongly encourage code deposition in a community repository (e.g. GitHub). See the Nature Portfolio guidelines for submitting code & software for further information.

Data

Policy information about availability of data

All manuscripts must include a data availability statement. This statement should provide the following information, where applicable:

- Accession codes, unique identifiers, or web links for publicly available datasets
- A description of any restrictions on data availability
- For clinical datasets or third party data, please ensure that the statement adheres to our policy

The raw clinical and imaging data are protected due to patient privacy protected due to patient privacy regulations and are available from the corresponding author upon reasonable request for 10 years. Data are located in controlled access data storage at the University Hospital Heidelberg. De-identified clinical data are available upon request. Sharing such data would require approval of the institutional ethics committees. De-identified data will then be transferred to the inquiring investigator over secure data file transfer. For the CheCUP panel design for targeted ccfDNA sequencing genome build hg19 NCBI Build 37.1/GRCh37 was used as reference. FFPE-DNA, FFPE-RNA and ccfDNA raw sequencing data were deposited into the European Genome-Phenome Archive (EGA) database under Study ID EGAD00001011130 (https://ega-archive.org/datasets/EGAD00001011130). The raw sequencing data are available under controlled access due to privacy policy regulations and in order to ensure that no data are used by for-profit organizations. Data are available upon reasonable request to the Data Access Committee (A. Krämer, A. Stenzinger, D. Kazdal, B. N. Kraft; contact: a.kraemer@dkfz.de). The EGA will then create an EGA account with the relevant permissions on our behalf. The processed sequencing data are available within the Source Data file. The study protocol is available as Supplementary Note. The Informed Consent and the Statistical Analysis Plan are available upon request. All remaining data that support the findings of this study and that are necessary to interpret, verify and extend the research in the article are available within the Article, the Supplementary Information or the Source Data file.

Research involving human participants, their data, or biological material

Policy information about studies with human participants or human data. See also policy information about sex, gender (identity/presentation), and sexual orientation and race, ethnicity and racism.

Reporting on sex and gender	For the purposes of this study, sex as a biological attribute was determined based on self-reporting. There are no study findings that apply to only one sex. Since no specific findings were expected according to gender, this information was not collected. Based on previous observations, sex was not expected to affect survival of the trial participants. For this reason, no sex-based analyses apart from descriptive frequency analysis were performed.
Reporting on race, ethnicity, or other socially relevant groupings	Study participants were not classified according to race, ethnicity or other socially relevant factors.
Population characteristics	The trial population reflected a typical CUP cohort in terms of histology, demographical and clinical characteristics. 15 male and 16 female patients were included. The median age at screening was 64 years (range 33-77). 64.5% of patients had adenocarcinoma, 16.1% squamous cell carcinoma and 12.9% undifferentiated carcinoma, while carcinomas with sarcomatoid differentiation were reported in 6.5% of cases. Patients had been previously treated with a median of two therapy lines (range 1-5), including platinum-based chemotherapy (39% carboplatin, 39% cisplatin, 34% oxaliplatin) as defined by the inclusion criteria. Ten patients (32.3%) had previously undergone radiotherapy to treat bone (16.1%), liver (3.2%) or lymph node metastases (9.7%).
Recruitment	As mentioned in the manuscript, the trial was initially planned to recruit 194 subjects, i.e. 97 subjects with high and intermediate/low TMB, respectively. Recruitment and treatment of subjects was performed in 10 trial sites.<br>As described previously, TMB was expected to be high versus intermediate/low in about 15% and 85% of CUP subjects (Gay et al. 2017; Krämer et al. 2018). As this asymmetric patient distribution between the two groups would lead to an increased sample size of subjects needed to be recruited into the trial in order to demonstrate a statistical significant difference in response to nivolumab plus ipilimumab treatment, a subject enrichment strategy was used to increase the ratio of subjects with TMB-high versus TMB-intermediate/low to 50 : 50. For that, TMB was analyzed by TSO500 panel for all subjects from the diagnostic FFPE tumor tissue sample which was available from initial diagnosis for each subject. If not enough biopsy material was available for genomic profiling, an additional biopsy had to be taken in order to allow for TMB determination before study entry. An interim futility analysis was scheduled after inclusion of 97 subjects in the full analysis set.<br>Since the trial was prematurely terminated, the both groups (TMG high and TMB low) were ultimately unbalanced. All sites are large university hospitals, community hospitals or oncological practices with dedicated, high-volume oncology units and

	large numbers of CUP subjects. In addition, each of the sites had a clinical trial centre and study nurses specifically dedicated to execution and documentation of the CheCUP study. Patients presenting at the participating sites and fitting the trial inclusion criteria were offered trial participation as a possible treatment option. Patients opting for treatment within the CheCUP trial were then screened as per protocol for study inclusion.
Ethics oversight	Before the start of the trial, the trial protocol, informed consent document, and all other appropriate documents were submitted to the independent Ethics Committee (EC) as well as to the competent authority (PEI).<br>A written favourable vote of the EC and an (implicit) approval by the competent authority were a prerequisite for initiation of this clinical trial. The statement of EC contains the title of the trial, the trial code, the trial site, and a list of reviewed documents. It mentions the date on which the decision was made and was officially signed by a committee member. This documentation also included a list of members of the EC present on the applicable EC meeting and a GCP compliance statement. The protocol was approved by the local ethics committees of the participating centers and the leading ethics committee of the Medical Faculty of the University of Heidelberg (date of approval: 27.11.2019) and the competent authorities (date of approval by the Paul Ehrlich Institute: 04.12.2019).

Note that full information on the approval of the study protocol must also be provided in the manuscript.

Field-specific reporting

Please select the one below that is the best fit for your research. If you are not sure, read the appropriate sections before making your selection.

[x] Life sciences [ ] Behavioural & social sciences [ ] Ecological, evolutionary & environmental sciences

For a reference copy of the document with all sections, see nature.com/documents/nr-reporting-summary-flat.pdf

Life sciences study design

All studies must disclose on these points even when the disclosure is negative.

Sample size	This is a non-randomized biomarker trial. Tumor mutational burden (TMB) is considered as biomarker. Subjects showing high TMB are considered biomarker-positive. A total of 194 subjects with 191 events are required to detect a hazard ratio of 0.65 for biomarker positive vs biomarker negative subjects with 80% power at the two-sided significance level of 5%. Median progression-free survival in the studied subject population is assumed to be 2.3 months, and 15% of subjects are expected to be biomarker-positive. Biomarker-positive subjects are expected to have a favorable prognosis. Assuming a hazard ratio of 0.65 for biomarker-positive versus biomarker-negative subjects and exponentially distributed survival, median survival times are 2.18 and 3.35 months for biomarker-negative and biomarker-positive subjects, respectively. Subjects should be recruited in a 1:1 ratio, i.e. biomarker-positive subjects should be enriched, which means that approximately 700 subjects need to be assessed for their TMB status. There should be a 24 months recruitment period and a minimal follow-up time of 12 months, i.e., a total trial duration of 36 months, which allows observing the necessary number of events under the above stated assumptions. An interim analysis was planned. Sample size was calculated with R package gsDesign (Keaven and Anderson, 2016) according to Lachin and Foulkes (1986).
Data exclusions	No data was excluded from this analysis.
Replication	This is a manuscript on a clinical trial without replication of data being feasible.
Randomization	This was not a randomized trial. Patients were stratified into TMB-high and TMB-low groups with a cut-off of 12 mutastions/Mb using the TruSight TSO500 sequencing panel.
Blinding	As this was no randomized trial blinding was not required.

Reporting for specific materials, systems and methods

We require information from authors about some types of materials, experimental systems and methods used in many studies. Here, indicate whether each material, system or method listed is relevant to your study. If you are not sure if a list item applies to your research, read the appropriate section before selecting a response.

Materials & experimental systems

n/a	Involved in the study	
[ ]	[x]	Antibodies
[x]	[ ]	Eukaryotic cell lines
[x]	[ ]	Palaeontology and archaeology
[x]	[ ]	Animals and other organisms
[ ]	[x]	Clinical data
[x]	[ ]	Dual use research of concern
[x]	[ ]	Plants

Methods

n/a	Involved in the study	
[x]	[ ]	ChIP-seq
[x]	[ ]	Flow cytometry
[x]	[ ]	MRI-based neuroimaging

Antibodies

Antibodies used	Ventana PD-L1, clone SP263. 3μm thick paraffin sections were prepared. Deparaffinizytion and tissue staining were performed using a Ventana Benchmark Ultra device (Roche Diagnostics, Mannheim, Germany). Slides were deparaffinized and incubated with cell conditioning solution (Cell Conditioning 1, CC1, Roche Diagnostics) at 96°C for 64 minutes. IHC staining was performed according to standard protocols (Ventana PD-L1 assay, clone SP263, Roche, Mannheim, Germany, Material number: 07208162001; Part number: 740-4907; incubation time: 16 minutes). Hematoxylin was used for counterstaining of cell nuclei. IHC stainings were evaluated by a specialist in pathology and scoring of PD-L1 was performed according to standardized scoring criteria.
Validation	Validation experiments were performed according to the manufacturer´s recommendations (CLSI. Quality assurance for design control and implementation of immunohistochemistry assay: approved guidelines. 2nd edition Wayne, PA, USA, 2011; Anatomic Pathology Checklist. College of American Pathologists. Jul 28, 2015).

Clinical data

Policy information about clinical studies

All manuscripts should comply with the ICMJEguidelines for publication of clinical research and a completedCONSORT checklist must be included with all submissions.

Clinical trial registration	EudraCT No. 2018-004562-33
Study protocol	The full trial protocol has been included in the submission
Data collection	In this trial a clinical data management system provided by the KKS was used for data collection using an electronic CRF (eCRF) for remote data entry (RDE).<br>All entries in the eCRF had to be verifiable by source documents. A detailed list of entries to eCRF was provided in the Investigator Site File. Regardless, there had to be a minimum documentation, which provided information on study participation and included all medical information necessary for appropriate medical care outside of the clinical trial in the patient record.<br>In addition, source documents had to mention that the subject had been included in an investigational study. Finally, there had to be no data that were inconsistent between eCRF and source documents.<br>All protocol-required information collected during the trial had to be entered by the investigator or a designated representative into the eCRF. Patient data was documented pseudonymously. The investigator or a designated representative should complete the eCRF pages as soon as possible after the information was collected, preferably within 2 weeks after the study visit. Any pending entries had to be completed immediately after the final examination. Explanation had to be given for all missing data.<br>The investigator was responsible for ensuring that all sections of the eCRF were completed correctly. Any errors had to be corrected in the eCRF and a reason for change had to be entered. The correctness of all entries in eCRF had to be confirmed by dated electronic signature of the responsible investigator. The time points and frequency of electronic signatures was defined in the study specific document 'eCRF specification´. The primary data collection took place locally at the treating site. Data was collected fro the data of informed consent until the discontinuation of study treatment. Following discontinuation of treatment, subjects were followed up 30 days after the last treatment or at initiation of another anti-cancer therapy (End of Treatment visit). Patients were then contacted by their physician at day 100 for a safety follow-up and date regarding adverse events were recorded until day 100 after the final dose of study treatment. Thereafter, data regarding survial were collected every 3 months. Data was collected between December 12th 2019 (first patient in) and March15th 2022. After the final data check, the database lock took place on June 15th 2022.
Outcomes	The primary analysis was performed by testing the null hypothesis of no difference in PFS between both biomarker groups using a log-rank test at a significance level of 5%. Secondary analyses of the primary endpoint comprised a multivariable Cox regression model including relevant prognostic factors. The secondary endpoint OS was analyzed similarly to PFS. Incidence and severity of adverse events were analyzed for the safety population. A detailed biometric analysis was defined in the statistical analysis plan which was authorized before database closure by the biometrician, the sponsor, and the PI.
